# A chromosome‐level genome of the spider *Trichonephila antipodiana* reveals the genetic basis of its polyphagy and evidence of an ancient whole-genome duplication event

**DOI:** 10.1093/gigascience/giab016

**Published:** 2021-03-19

**Authors:** Zheng Fan, Tao Yuan, Piao Liu, Lu-Yu Wang, Jian-Feng Jin, Feng Zhang, Zhi-Sheng Zhang

**Affiliations:** Key Laboratory of Eco-Environments in Three Gorges Reservoir Region (Ministry of Education), School of Life Sciences, Southwest University, No.2 Tiansheng Road, Beibei District, Chongqing 400715, China; Key Laboratory of Eco-Environments in Three Gorges Reservoir Region (Ministry of Education), School of Life Sciences, Southwest University, No.2 Tiansheng Road, Beibei District, Chongqing 400715, China; Key Laboratory of Eco-Environments in Three Gorges Reservoir Region (Ministry of Education), School of Life Sciences, Southwest University, No.2 Tiansheng Road, Beibei District, Chongqing 400715, China; Key Laboratory of Eco-Environments in Three Gorges Reservoir Region (Ministry of Education), School of Life Sciences, Southwest University, No.2 Tiansheng Road, Beibei District, Chongqing 400715, China; Department of Entomology, College of Plant Protection, Nanjing Agricultural University, No.1 Weigang Road, Nanjing, Jiangsu 210095, China; Department of Entomology, College of Plant Protection, Nanjing Agricultural University, No.1 Weigang Road, Nanjing, Jiangsu 210095, China; Key Laboratory of Eco-Environments in Three Gorges Reservoir Region (Ministry of Education), School of Life Sciences, Southwest University, No.2 Tiansheng Road, Beibei District, Chongqing 400715, China

**Keywords:** Hi-C, high‐quality genome, whole-genome duplication, gene family analysis, cytochrome P450, ABC, CCE, GST; Hox

## Abstract

**Background:**

The spider *Trichonephila antipodiana* (Araneidae), commonly known as the batik golden web spider, preys on arthropods with body sizes ranging from ∼2 mm in length to insects larger than itself (>20‒50 mm), indicating its polyphagy and strong dietary detoxification abilities. Although it has been reported that an ancient whole-genome duplication event occurred in spiders, lack of a high-quality genome has limited characterization of this event.

**Results:**

We present a chromosome‐level *T. antipodiana* genome constructed on the basis of PacBio and Hi-C sequencing. The assembled genome is 2.29 Gb in size with a scaffold N50 of 172.89 Mb. Hi‐C scaffolding assigned 98.5% of the bases to 13 pseudo-chromosomes, and BUSCO completeness analysis revealed that the assembly included 94.8% of the complete arthropod universal single-copy orthologs (n = 1,066). Repetitive elements account for 59.21% of the genome. We predicted 19,001 protein-coding genes, of which 96.78% were supported by transcriptome-based evidence and 96.32% matched protein records in the UniProt database. The genome also shows substantial expansions in several detoxification-associated gene families, including cytochrome P450 mono-oxygenases, carboxyl/cholinesterases, glutathione-*S*-transferases, and ATP-binding cassette transporters, reflecting the possible genomic basis of polyphagy. Further analysis of the *T. antipodiana* genome architecture reveals an ancient whole-genome duplication event, based on 2 lines of evidence: (i) large-scale duplications from inter-chromosome synteny analysis and (ii) duplicated clusters of Hox genes.

**Conclusions:**

The high-quality *T. antipodiana* genome represents a valuable resource for spider research and provides insights into this species’ adaptation to the environment.

## Data Description

### Background

Spiders (Araneae) have a worldwide distribution, have conquered virtually all terrestrial environments, and exhibit considerable species richness. A total of 49,200 spider species have been described to date, classified into 4,208 genera and 128 families [1]. Spiders are notable with respect to their numerous distinctive characteristics, including the production of silk [2] and venom [3], prolonged milk provisioning [4], foraging behavior [5], sexual size dimorphism [6], and whole-genome duplications (WGDs) [7].

To date, the genomes of 11 species of spider have been published or are available in the NCBI database (Table 1), which offer unprecedented insights into the unique biology of these arthropods. For example, complex sets of venom and silk genes have been identified in the genomes of *Stegodyphus mimosarum, Acanthoscurria geniculata*, and *Trichonephila clavipes* (formerly *Nephila clavipes*) [8–10]. The role of DNA methylation in spider gene regulation has been demonstrated in the genome of *Stegodyphus dumicola* [11]. And components of the spider immune system were initially characterized with reference to the genome of *Parasteatoda tepidariorum, S. mimosarum*, and *A. geniculata* [12,13].

**Table 1:** Comparison of the quality of the *Trichonephila antipodiana* genome with that of other published spider genomes

Species	Genome size (Gb)	Scaffold N50 (kb)	Contig N50 (kb)	Accession No.
*Stegodyphus dumicola*	2.55	254.13	254.13	GCA_010614865.1
*Anelosimus studiosus*	2.03	4.79	1.13	GCA_008297655.1
*Pardosa pseudoannulata*	4.21	711.40	23.23	GCA_008065355.1
*Latrodectus hesperus*	1.23	39.47	15.96	GCA_000697925.2
*Dysdera silvatica*	1.36	38.02	25.71	GCA_006491805.1
*Loxosceles reclusa*	3.26	63.24	1.83	GCA_001188405.1
*Trichonephila clavipes*	2.44	62.96	7.99	GCA_002102615.1
*Parasteatoda tepidariorum*	1.45	4,055.36	10.15	GCA_000365465.3
*Stegodyphus mimosarum*	2.74	480.64	40.15	GCA_000611955.2
*Araneus ventricosus*	3.65	59.62	–	BGPR01000001-BGPR01300721 (DDBJ)
*Argiope bruennichi*	1.67	124,236.00	288.40	GCA_015342795.1
*Trichonephila antipodiana*	2.29	172,892.00	1,138.00	–

The spider genomes tend to be difficult to sequence, assemble, and annotate owing to their large size, high heterozygosity, and repeat content. To date, the genomes of only 3 species (*S. dumicola, Dysdera silvatica*, and *Argiope bruennichi*) have been sequenced based on long sequencing reads (PacBio or Nanopore), only 1 of which was assembled to the chromosome level [11,14,15]. Lack of high-quality genome data has severely hampered deep spider research. In this study, we combined Pacific Biosciences (PacBio) and high-throughput chromosome conformation capture (Hi-C) sequencing to produce a high-quality, chromosome-level reference genome for *Trichonephila antipodiana*, and describe the salient features of the *T. antipodiana* genome, focusing on genome assembly, annotation, and evolutionary analyses.

**Figure 1:**
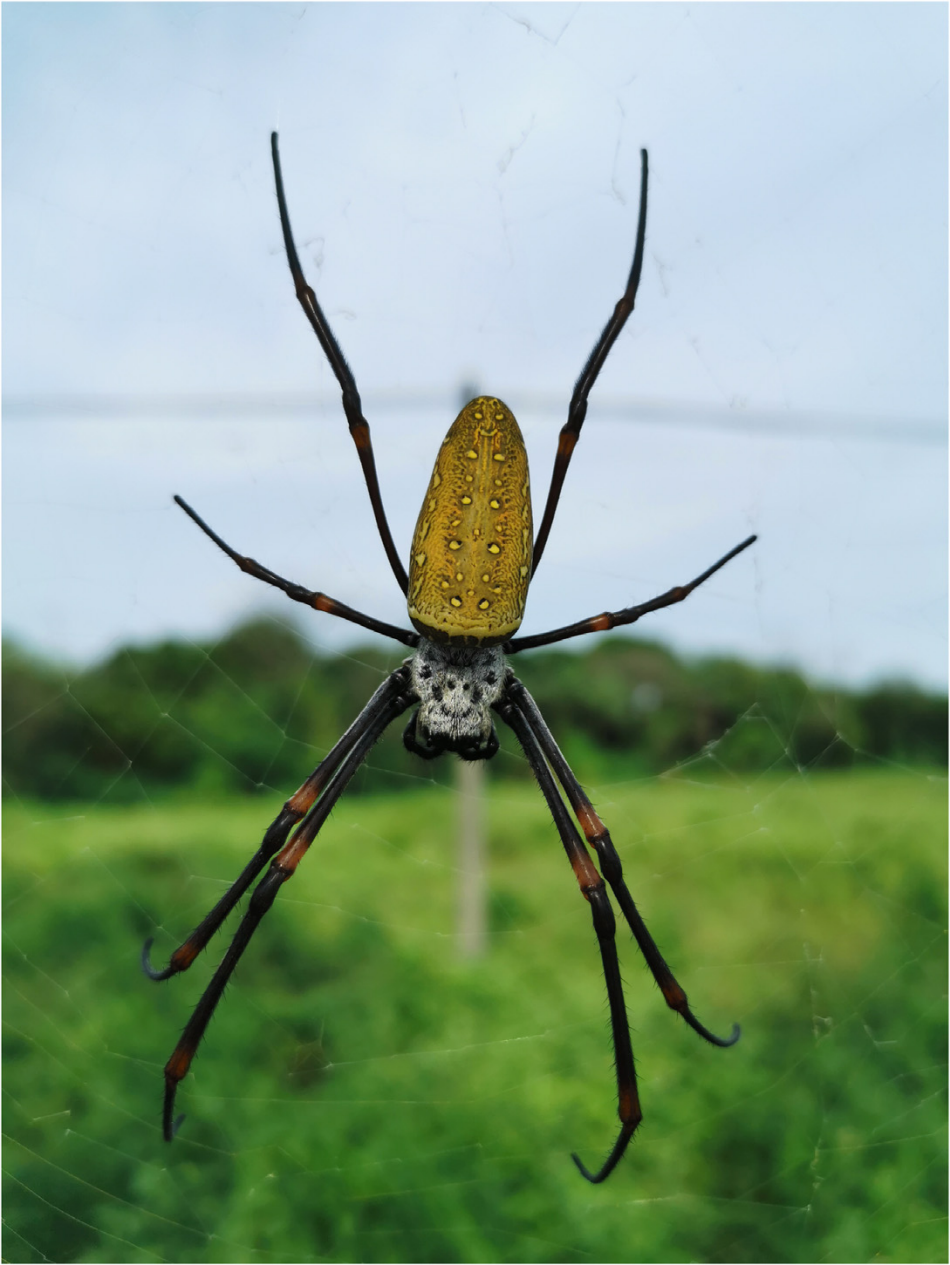
Habitus of *Trichonephila antipodiana*, female.

The batik golden web spider, *T. antipodiana* (Fig. 1), one of the typical Nephilinae species in the family Araneidae, is recorded from a number of countries, including Australia (Queensland), the Solomon Islands, New Guinea, the Philippines, and China (Hainan Island) [1, 16]. Recently, in addition to many taxonomic articles that have provided a clear outline of species in the Nephilinae, numerous studies on this subfamily have focused on their silk characteristics and sexual size dimorphism [6, 17, 18]. The webs constructed by *T. antipodiana* are ∼1.0 m in diameter and can deal with a large size range of any suitable prey, including various species of Araneae, Crustacea, Formicidae, Isoptera, Orthoptera, Diptera, Coleoptera, Lepidoptera, Hymenoptera, Odonata, and even small birds, which thereby indicates their polyphagy and strong detoxification abilities [16]. Furthermore, it has been reported that when recycling their orb webs, these spiders may also feed on adhering pollen grains or fungal spores via extraoral digestion [19].

The process of enzymatic detoxification of xenobiotics in cells converts a lipophilic, non-polar xenobiotic into a more water-soluble and therefore less toxic metabolite, which can then be eliminated more easily from the cell. Cytochrome P450 represents a superfamily of enzymes responsible for the Phase 1 metabolism of drugs and foreign compounds, which are involved in catalyzing the mono-oxygenation of a diverse array of xenobiotic and endogenous compounds [20]. The carboxyl/cholinesterase (CCE) superfamily is composed of functionally diverse proteins that hydrolyze carboxylic esters and also plays an important role in detoxification of exogenous compounds in the diet or in the environment [21]. Glutathione S-transferase (GST) is involved in catalyzing the conjugation of activated xenobiotics to an endogenous water-soluble substrate, such as reduced glutathione, UDP-glucuronic acid, or glycine [22]. The ATP-binding cassette transporters (ABC) protein family is one of the largest transporter families; toxic metabolites can be transported out of the cell via the action of ABC transporters [23]. In insects, the size of xenobiotic detoxification gene families may be associated with the complexity of their diets [24]. For example, in Hymenoptera species, there are relatively few members of these families in the honeybee *Apis mellifera* genome compared with *Nasonia vitripennis*, which is thought to encounter a wider range of potentially toxic xenobiotics in their diet and habitat [25, 26]. To investigate the polyphagy and detoxification of this spider, we analyzed a selection of detoxification-associated gene families, including P450 mono-oxygenases, CCE, GST, and ABC.

WGD is a process of genome doubling that supplies raw genetic material and increases genome complexity. It can provide new genetic material that enables paralogous genes to undergo sub- or neo-functionalization, which can contribute to the rewiring of gene regulatory networks, morphological innovations, and, ultimately, organismal diversification. It has been reported that an ancient WGD event occurred in the common ancestor of spiders and scorpions. In spiders, the first evidence of a duplication event was detected in the genome of the house spider *P. tepidariorum*, as indicated by a high number of duplicated genes, including 2 clusters of Hox genes [7]. In view of the importance of the WGD event in spiders, to gain more evidence in support of the WGD event, we performed synteny and Hox gene analyses in *T. antipodiana*.

The *T. antipodiana* reference genome described herein will lay a foundation for further research on the unique characteristics and functions of spiders.

## Methods

### Sample collection and sequencing

The female specimens of *T. antipodiana* used in this experiment were obtained from Shiwan Township, Hefu County, Beihai City, Guangxi Province, China, and was stored at −80°C prior to DNA extraction. The spider, excluding the abdomen, was prepared for PacBio and Illumina whole-genome sequencing, and leg muscle tissue was used for Illumina transcriptome sequencing.

Genome sequencing was performed by Berry Genomics (Beijing, China). Genome DNA for PacBio and Illumina sequencing was isolated using a Qiagen Blood & Cell Culture DNA Mini Kit. PacBio Sequel II libraries for PacBio sequencing were constructed with insert sizes of 20 kb using a SMRTbell™ Template Prep Kit 1.0-SPv3. Two short paired-end insert libraries containing 350-bp sequences were constructed for survey analysis using a Truseq DNA PCR-free kit and sequenced using the NovaSeq 6000 platform.

For the purposes of Hi-C sequencing, the muscle tissues of the single female specimen were fixed with formaldehyde and lysed, and the cross-linked DNA was subsequently digested overnight with MboI. Sticky ends were biotinylated and proximity-ligated to form chimeric junctions that were enriched for and then physically sheared to a size of 350  bp. Chimeric fragments representing the original cross-linked long-distance physical interactions were then processed into paired-end sequencing libraries, and 150-bp paired-end reads were generated using the Illumina HiSeq PE150 platform.

Muscle RNA was extracted using TRIzol (Invitrogen) according to the manufacturer's instructions.

### Genome survey and assembly

Quality control of the raw Illumina data was performed using BBTools suite v38.67 (Bestus Bioinformaticus Tools, RRID:SCR_016968) [27]. The duplicates were removed using “clumpify.sh,” and then “bbduk.sh” was used to trim the reads' ends to Q20 with reads shorter than 15 bp or with >5 Ns. The poly-A/G/C tails of ≥10 bp were trimmed, and the overlapping paired reads were corrected using “bduk.sh.”All filtered reads were used to estimate genome size and other characteristics. In addition, a 21-mer was selected for *k*-mer analysis and the *k*-mer distribution was estimated using “khist.sh” (BBTools). The 21-mer depth frequency distribution was calculated using GenomeScope v1.0.0 (GenomeScope, RRID:SCR_017014) [28], and the maximum *k*-mer coverage cut-off was set to 10,000.

For the long reads generated using the PacBio Sequel platform, contig assembly of the *T. antipodiana* genome was conducted using Flye v2.5 (Flye, RRID:SCR_017016) [29] with a single round of polishing and the minimum overlap between reads was set to 3,000. Heterozygous regions of the assembly were removed using Purge Haplotigs v1.1.0 [30], with a 50% cut-off for identifying contigs as haplotigs. Illumina reads were used to polish the assembly using NextPolish v1.0.5 [31] over 2 rounds. During all the Flye and NextPolish polishing steps, Minimap2 v2.12 (Minimap2, RRID:SCR_018550) [32] was used as the read aligner.

The Hi-C reads were used to generate a chromosome-level assembly of the genome, and 3 software packages were used for analysis. The reads were initially subjected to quality control to remove the duplicates and then aligned to the genome using Juicer v1.6.2 (Juicer, RRID:SCR_017226) [33]. The resulting alignment BAM file was then transformed to a BED format and fed to SALSA v2.2 [34] to correct the obvious misjoin errors between contigs. The alignment BAM file was also mapped to the cleaned assembly data using Minimap2. Finally, the data were fed to Allhic v0.9.13 [35] to anchor contigs to chromosomes.

Potential contaminant sequences were inspected using HS-BLASTN [36] and BLAST+ (blastn) v2.7.1 [37] against the NCBI nucleotide (nt) and UniVec databases.

Genome completeness was assessed using the BUSCO v3.0.2 pipeline (BUSCO, RRID:SCR_015008) [38] against an arthropod reference gene set using the arthropoda_odb 9 database of the genome (n = 1,066). To evaluate the mapping rate, the clean reads of the Illumina or PacBio sequences were mapped to the reference genome using Minimap2.

### Genome annotation

Genome annotation essentially encompasses 4 aspects: repeat, protein-coding gene, non-coding RNA (ncRNA), and gene function annotations.

We searched for repetitive elements in the assembled genome by means of a combination of *ab initio* and homology-based searching. Initially, we constructed a specific repeat database using RepeatModeler v2.0.1 (RepeatModeler, RRID:SCR_015027) [39] and thereafter combined the *ab initio* database and known repeat library (Repbase) [40] as the reference repeat database. To identify repetitive elements, we used RepeatMasker (RepeatMasker, RRID:SCR_012954) [41] to search against the reference repeat database. The ncRNAs were identified using Infernal v1.1.2 (Infernal, RRID:SCR_011809) [42] and tRNAscan-SE v2.0.6 (tRNAscan-SE, RRID:SCR_010835) [43], and transfer RNAs (tRNAs) of high confidence were confirmed using the tRNAscan-SE script “EukHighConfidenceFilter.”

Using the repeat-masked genome, we used Maker v2.31.10 (Maker, RRID:SCR_005309) for genome annotation by integrating *ab initio*, transcriptome-based, and protein homology–based evidence [44]. Augustus v3.3.2 (AUGUSTUS, RRID:SCR_008417) [45] and GeneMark-ES/ET/EP v4.48_3.60_lic [46] were used for *ab initio* gene prediction. To accurately model the sequence properties, both gene finders were initially trained using the BRAKER v2.1.5 pipeline (BRAKER, RRID:SCR_018964) [47], which makes use of the mapped transcriptome sequence data. Previously, RNA-seq data were mapped to our genome assembly using HISAT2 v 2.2.0 (HiSat2, RRID:SCR_015530) [48]. BRAKER was then run with default parameters. The RNA-seq data were further assembled into transcripts using Stringtie v2.1.3 [49], with the assembled genome used as a reference. The resulting transcripts were provided as input for Maker via the “est” option. The protein sequences of *Drosophila melanogaster* (GCA_000001215.4), *Ixodes scapularis* (GCA_002892825.2), *S. mimosarum* (GCA_000611955.2), *T. clavipes* (GCA_002102615.1), *P. tepidariorum* (GCA_000365465.3), *Strigamia maritima* (GCA_000239455.1), and *Daphnia pulex* (GCA_900092285.2) were downloaded from the NCBI database as protein homology–based evidence required by Maker.

The functions of the predicted protein sequences were assigned against the UniProtKB/Swissprot database using Diamond v0.9.24 (Diamond, RRID:SCR_016071) [50] with a more sensitive mode, 1 maximum number of target sequences, to report alignments with an e-value threshold of 1e−5.

Annotation of the protein domains was based on Gene Ontology (GO) and Reactome pathways of the predicted protein-coding genes, with InterProScan v5.41–78.0 (InterProScan, RRID:SCR_005829) [51] being used to screen proteins against the following 5 databases: Pfam [52], Panther [53], Gene3D [54], Superfamily [55], and Conserved Domain Database (CDD) [56].

Using eggNOG-mapper v2.0 [57], the eggNOG v5.0 database [58] was used for GO, expression coherence (EC), KEGG pathways, KEGG orthologous groups (KOs), and clusters of orthologous groups (COG) functional category annotation of the predicted protein-coding genes.

To assess the completeness of the *T. antipodiana* protein annotation, we used the protein mode of the BUSCO v3.0.2 (BUSCO, RRID:SCR_015008) pipeline and the arthropod reference set of arthropoda_odb 9 (n = 1,066) [38].

### Phylogenetic analyses and GO/KEGG enrichment analyses

Orthologous gene clusters were classified using OrthoFinder v2.3.8 (OrthoFinder, RRID:SCR_017118) [59] across the well-annotated and well-assembled genomes of 10 species covering representative Chelicerata lineages along with *T. antipodiana*: 1 Scorpiones (*Centruroides sculpturatus*, GCA_000671375.2), 5 Acari (*Dermatophagoides pteronyssinus*, GCA_001901225.2; *Galendromus occidentalis*, GCA_000255335.1; *Tetranychus urticae*, GCA_000239435.1; *Varroa destructor*, GCA_002443255.1; *I. scapularis*, GCA_002892825.2), 3 Araneae (*P. tepidariorum*, GCA_000365465.3; *S. mimosarum*, GCA_000611955.2; *T. clavipes*, GCA_002102615.1), and 1 Xiphosura (*Tachypleus tridentatus*). With the exception of *T. tridentatus* [60], most protein sequences were downloaded from the NCBI database.

To infer the phylogeny of these species, the protein sequences of 236 single-copy genes were separately aligned using MAFFT v7.394 (MAFFT, RRID:SCR_011811) [61] based on the L-INS-I strategy. The resulting alignments were trimmed using trimAl v1.4.1 (trimAl, RRID:SCR_017334) [62] to remove sites of unclear homology using the heuristic method “automated1.” The resulting alignments were concatenated using FASconCAT-G v1.04 [63]. Genes that violated the models were removed prior to tree inference. Finally, maximum likelihood reconstructions were performed using IQ-TREE v2.0.7 (IQ-TREE, RRID:SCR_017254) [64] with extended model selection followed by tree inference, model set by LG, with the number of partition pairs for the rcluster algorithm, replicates for ultrafast bootstrap, and Shimodaira–Hasegawa approximate likelihood ratio tests being 1,000, 10, and 1,000, respectively.

The divergence time was estimated with MCMCTree within the package PAML v4.9j (PAML, RRID:SCR_014932) [65] using parameters with independent clock rates; BDparas-related birth, death, and sampling rates of 1, 10, and 0.1, respectively; kappa_gamma of 62; alpha_gamma of 11; rgene_gamma of 2,201; and sigma2_gamma of 1,101. Fossil records were derived from the paleobiodb database [66] and the recently described fossils *Eramoscorpius brucensis* [67] and *Parioscorpio venator* [68], with Chelicerata (genus *Paleomerus*, 516‒541 million years ago [Mya]), Parasitiformes (*Deinocroton draculin*, 93.5‒145.5 Mya), and Arachnopulmonata (*E. brucensis* and *P. venator*, 435‒439 Mya).

Café v4.2.1 (CAFÉ, RRID:SCR_005983) [69] was used to identify the likelihood of gene family expansion and contraction using the single birth–death parameter λ and a *P*-value threshold of 0.01. GO and KEGG functional enrichment of the significantly expanded families was assessed using Tbtools v1.045 [70].

### Annotation of dietary detoxification-related gene families

To manually annotate the genes of detoxification-related enzymes (P450s, CCEs, GSTs, and ABCs), we initially downloaded the amino acid sequences of the P450s, CCEs, GSTs, and ABCs predicted from the *D. melanogaster, Bombyx mori*, and *T. urticae* sequences obtained from NCBI.

For cytochrome P450 proteins, we performed a blastp-like search using MMsesqs2 v11 [71] with 4 rounds of iteration because the identity between 2 proteins can be as low as 25%. Using the Pfam database, Interproscan v5.41–78.0 (Interproscan, RRID:SCR_005829) [72] was used to confirm specific conserved domains of the P450 sequences. And every P450 protein was checked for structure characteristics including 4-helix bundles (D, E, I, and L), helices J and K, 2 sets of β sheets, and a coil referred to as the “meander.” The regions comprise a heme-binding loop, a strictly conserved Glu-X-X-Arg motif in helix K, and a consensus sequence (Ala/Gly-Gly-X-Asp/Glu-Thr-Thr/Ser) in the central part of helix I [73]. We deleted the invalid matches of the proteins using MMsesqs2 with a tblatn-like search, and each protein was also examined to identify intron/exon boundaries.

Members of the other 3 detoxification enzyme gene families (CCEs, GSTs, and ABCs) of *T. antipodiana* were identified using MMsesqs2 v11 using a blastp-like search with 4 rounds of iteration and an e-value of 0.001. Interproscan v5.41–78.0 (Interproscan, RRID:SCR_005829) was used to confirm the specific conserved domains of genes using the Pfam database. Classification and functional categories of the resulting HMMER-Pfam below were further checked using an online NCBI BLASTP of the non-redundant (nr) GenBank protein database. Each protein was assessed for intron/exon boundaries, and extremely short or long sequences were removed. Finally, the multi-hits were reduced to the same gene region and we deleted the invalid matches of the proteins using MMsesqs2 with a tblatn-like search.

We also conducted an analysis of the sequence evolution of the specific gene families such as cytochrome P450, CCE, GST, and ABC. Initially, the proteins were aligned using MAFFT v7.450 with common parameters, after which the resulting alignments were trimmed using trimAl v1.4.1 to remove the sites with unclear homology based on the heuristic method “automated1.” Finally, gene trees were constructed using IQ-TREE v2.0.7 with an LG model and 1,000 ultrafast bootstrap replicates.

To obtain the P450 gene expression in the whole body of *T. antipodiana*, we count the number of P450 genes from the RNA data using FeatureCounts [74] software. RNA-seq data were mapped to our genome assembly using HISAT2 v 2.2.0 previously.

### WGD analyses

It has been reported that an ancient WGD event occurred in the common ancestor of spiders and scorpions, and in an attempt to confirm the occurrence of this event, we examined 2 possible lines of evidence.

We conducted an intra-specific analysis of the synteny between *T. antipodiana* chromosomes. *T. antipodiana* proteins were searched against themselves with MMsesqs2 v11 using a blastp-like search with 3 rounds of iteration and an e-value of 0.001. The blast results and gene annotation GFF3 file were fed to MCScanX [75] with an e-value threshold of 1e−8. A collinear block was defined by a homologous region shared by 4 or more gene sequences with no rearrangements.

In arthropods, 10 highly conserved Hox genes that are inferred to occur in the common ancestor of Panarthropoda play important roles [76]. In the present study, we manually annotated the Hox genes of *T. antipodiana*, using the Hox protein amino acid sequences predicted for *Daphnia magna, P. tepidariorum, C. sculpturatus, I. scapularis*, and *D. melanogaster* downloaded from the NCBI database. MMsesqs2 v11 was used to perform a blastp-like search for 4 rounds of iteration with an e-value of 0.001. The Hox gene clusters classification and functional categories of the resulting BLAST below were further assessed using the HomeoDB database [77].

The locations of the Hox genes were further confirmed on the basis of genome annotation, and Hox gene clusters and synteny blocks were plotted across chromosomes using Tbtools.

## Results and Discussion

### A high-quality genome among Araneae

In this study, we constructed a chromosome‐level *T. antipodiana* genome based on PacBio and Hi-C sequencing.

Sequencing yielded 767.07 Gb of clean data, comprising 305.96 Gb Illumina (133×), 235.79 Gb PacBio (103×), 215.05 Gb Hi-C (94×), and 10.27 Gb transcriptome reads. The long PacBio subreads had mean N50 lengths of 14.81 and 21.19 kb, respectively. The detailed sequencing data are summarized in Table 2.

**Table 2:** Statistics of the DNA sequence data used for genome assembly

Paired-end libraries	Clean data (Gb)	Sequencing coverage (×)	Insert sizes (bp)
Illumina reads	305.96	133	300
PacBio reads	235.79	103	20
Hi-C	215.05	94	300
RNA	10.27		300
Total	767.07		

A *k*-mer analysis indicated that the number of unique *k*-mers peaked at 21 and predicted a genome assembly size of 2.15 Gb (Supplementary Fig. S1), which is in general agreement with the recent draft genome of *T. clavipes* (2.44 Gb).

Using the Flye assembler, we obtained an initial 2.38 Gb genome assembly with a contig N50 of 1.17 Mb. To enhance the draft assemblies, haplotigs and contig overlaps were removed from the genome. The total length of the assembly was 2.31 Gb, with a contig N50 of 1.23 Mb. Finally, Hi-C data were used for genome scaffolding with a mapping rate of 89.16%, and a high-quality chromosome-level genome assembly of *T. antipodiana* was accordingly obtained with a total length of 2.29 Gb, a contig N50 of 1.14 Mb, and a scaffold N50 of 172.89 Mb (Table 3). The genome of *T. antipodiana* is 1 of the 2 chromosome-level genomes obtained for spiders to date, the other being that of *A. bruennichi* [15]. A comparison of the genome assembly obtained in the present study with that of the congeneric species *T. clavipes* indicated the superior quality of the *T. antipodiana* assembly, with a scaffold N50 of 172 Mb compared with that of 62.96 kb obtained for *T. clavipes* (Table 1).

**Table 3:** Summary of each step in construction of the *Trichonephila antipodiana* genome assembly

Assembly	Total length	No. scaffolds (chromosome)	N50 length	Longest scaffold	GC (%)	BUSCO (n = 1,066) (%)			
						C	D	F	M
Flye	2.38 Gb	16,680	1.21 Mb	11.071 Mb	31.8	95.2	5.2	0.9	3.9
Purge Dups	2.31 Gb	10,670	1.26 Mb	11.071 Mb	31.8	95.3	4.0	0.8	3.9
Pilon	2.31 Gb	10,670	1.26 Mb	11.082 Mb	31.7	95.3	4.3	0.7	4.0
Hi-C	2.29 Gb	377 (13)	137.66 Mb	230.27 Mb	31.7	94.8	4.1	1.0	4.2
Final genome assembly	2.29 Gb	377 (13)	137.66 Mb	230.17 Mb	31.7	94.8	4.1	1.0	4.2
Transcript assembly	69.29 Mb	30,586	3.43 kb	43.99 kb	34.3	97.2	33.4	1.1	1.7

C: complete; D: duplicated; F; fragmented; M: missing.

BUSCO is a tool used to assess the completeness of genome/transcriptome assemblies and annotated proteins based on single-copy orthologs, and the BUSCO results obtained in the present study indicated that 1,011(94.8%) of the 1,066 orthologs in a reference arthropod data set (arthropoda_odb9) were labeled as complete in our assembly, which is similar to the value obtained for *T. clavipes* (94.85%). The results of BUSCO analysis at all steps in the assembly of the *T. antipodiana* genome are reported in Table 3.

The mapping rate, which is defined as the proportion of high-throughput sequencing reads that are uniquely mapped to a reference genome, reflects the accuracy of the assembly, and in the present study, we obtained mapping rates of 96.78%, 97.23%, and 97.61% for the RNA-seq, Illumina, and PacBio reads, respectively.

### Gene annotation

**Figure 2:**
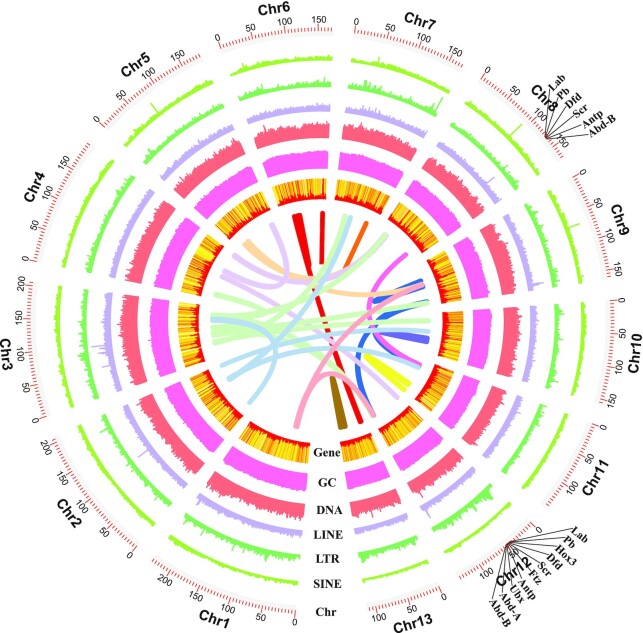
Schematic representation of the genomic characteristics of *Trichonephila antipodiana*. The inner ring of the circle is based on the findings of inter-chromosome synteny analysis; the outer rings of the circle represent the distribution of genes, GC content, DNA elements, long interspersed nuclear elements (LINEs), long terminal repeats (LTRs), short interspersed nuclear elements (SINEs), and chromosomes. The location of Hox genes is marked on the outer ring of the chromosome circle.

The *T. antipodiana* genome comprises 59.21% repetitive elements, including 57.12% transposable elements (TEs), 0.72% small RNAs, 0.13% satellites, 1.08% simple repeats, and 0.19% low-complexity regions (Table 4). The TEs are predominantly represented by 5 categories of abundant repeats, unclassified (22.08%), DNA transposon elements (22.42%), long interspersed nuclear elements (LINEs, 3.61%), long terminal repeats (LTRs, 3.45%), and short interspersed nuclear elements (SINEs, 1.10%). An analysis of the distribution of repetitive elements in the *T. antipodiana* genome revealed that DNA transposon elements are highly distributed in the genome regions (Fig. 2), with TcMar-Tc1 and hAT-Charlie being identified as the most common DNA transposon elements, accounting for 7.18% and 6.19%, respectively. We found that the percentage of DNA transposon elements in *T. antipodiana* is higher than that in some other species of spider, including *Argiope bruennichi* (6.27%), *Trichonephila clavipes* (13.71%), *Araneus ventricosus* (14.45%), *Dysdera silvatica* (19.58%), *Stegodyphus dumicola* (16.17%), *S. mimosarum* (18.77%), *Pardosa pseudoannulata* (16.55%), *Loxosceles reclusa* (10.23%), *Anelosimus studiosus* (7.94%), *Latrodectus hesperus* (7.03%), and *P. tepidariorum* (6.9%) [15].

**Table 4:** Statistics of the repetitive sequences identified in *Trichonephila antipodiana*

Type	No.	Length (bp)	% of genome
SINEs	106,507	25,417,898	1.11
tRNA-Deu	44,262	10,710,146	0.47
MIR	28,198	6,417,754	0.28
tRNA-Core	19,575	5,140,899	0.22
tRNA	3,964	507,398	0.02
LINEs	197,390	83,281,087	3.63
Penelope	49,982	30,623,444	1.33
I	56,156	19,510,142	0.85
I-Jockey	27,196	13,846,368	0.60
R1	14,033	5,652,471	0.25
LTR elements	101,690	79,698,444	3.47
Gypsy	53,122	53,035,139	2.31
Pao	26,368	19,084,383	0.83
Copia	15,295	6,965,923	0.30
ERV1	4,052	178,070	0.01
DNA elements	1,393,742	518,114,026	22.58
TcMar-Tc1	332,152	164,809,170	7.18
hAT-Charlie	399,282	142,099,848	6.19
TcMar-Mariner	89,418	39,370,728	1.72
Kolobok-Hydra	37,030	30,581,663	1.33
Unclassified	1,961,792	508,599,211	22.17
Total interspersed repeats		1,215,110,666	52.96
Small RNA	72,066	16,577,914	0.72
Satellites	7,513	2,910,802	0.13
Simple repeats	450,644	24,805,223	1.08
Low complexity	84,430	4,336,839	0.19

**Figure 3:**
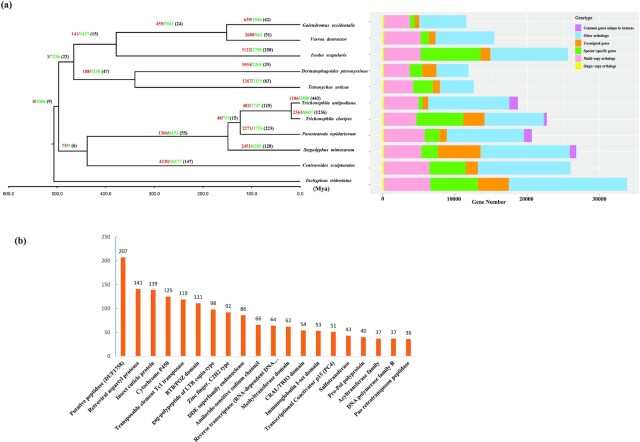
Phylogenetic and comparative gene family analyses of *Trichonephila antipodiana* and other Arachnida species. The estimated species divergence times (millions of years ago [MYA]) are indicated at each branch point. Node values indicate gene families showing expansion (red), contraction (green), and rapid evolution (black in parentheses). The bar chart indicates the number of genes classified into 6 groups (single-copy, multi-copy, species-specific, unassigned, other, and common genes unique to Araneae).

Using the MAKER2 genome annotation tool, we identified 19,001 protein-coding genes in the *T. antipodiana* genome, with a mean number of 7.24 exons and 6.12 introns per gene, and mean exon and intron lengths of 247.46 bp and 3.73 kb, respectively. On the basis of BUSCO analysis, we identified 1,027 (96.3%) complete, 60 (5.6%) duplicated, 14 (1.3%) fragmented, and 25 (2.4%) missing orthologs. Furthermore, we found that a total of 18,303 (96.33%) genes had ≥1 record in the SwissProt or TrEMBL databases. InterProScan and EggOG analyses identified the protein domains for 14,705 (77.39%) genes, 12,226 GO terms, 9,465 KEGG ko terms, 5,788 KEGG pathways, 14,325 COG categories, and 3,183 Enzyme Codes. Comparatively, 22,689 protein-coding genes have been identified in the *T. clavipes* genome, which is approximately comparable to the number in *T. antipodiana* (Fig. 3a).

We identified 4,452 ncRNA-associated loci in the squid sequencing data and found that all the essential and well-conserved metazoan ncRNAs are also present in the *T. antipodiana* genome: 3,653 tRNAs, 160 ribosomal RNAs (5S, 5.8S, small subunit, and large subunit), 2 RNase P, 1 RNase MRP, 22 SRP, 216 major spliceosomal small nuclear RNAs (U1, U2, U4, U5, U6), 26 minor spliceosomal small nuclear RNAs (U11, U12, U4atac, and U6atac), and 6 CD-boxes.

### Gene orthology and comparative analysis with other genomes

Identifying homologous relationships among the sequences of different species plays a pivotal role in enhancing our understanding of evolution and diversity. In this regard, we compared the protein-coding genes of *T. antipodiana* with those of 10 representative Arachnida species, including 3 species of spider (*P. tepidariorum, S. mimosarum*, and *T. clavipes*), 1 Scorpiones (*C. sculpturatus*), and 5 Acari (*D. pteronyssinus, G. occidentalis, T. urticae, V. destructor*, and *I. scapularis*) to identify orthologous groups, with *T. tridentatus* being used as an outgroup. Using OrthoFinder, we obtained a total of 203,348 genes among the 11 species, which were clustered into 20,785 orthogroups. We also count the genes of single-copy and multi-copy orthologs, common genes unique to Araneae, species-specific genes, and other unassigned orthologous genes among the 11 species (Fig. 3a). Gene family analysis also revealed that among these species, 152 gene families and 590 genes were unique to *T. antipodiana*.

To gain an understanding of Arachnida genomic evolution, we reconstructed a phylogenomic tree of the 11 assessed species based on 236 single-copy orthologous genes, which were calibrated using 4 fossil records. The phylogenomic tree obtained indicated that Scorpiones (*C. sculpturatus*) show a close relationship with spiders, and we estimated that *T. antipodiana* and *T. clavipes* diverged ∼16.15–19.62 Mya (Fig. 3a).

### Gene family evolution and GO/KEGG enrichment analyses

Within the *T. antipodiana* genome, we identified 1,186 expanded and 2,480 contracted gene families (*P* ≤ 0.01), among which 300 and 143 families have undergone significant expansions and contractions (*P* < 0.001), respectively (Fig. 3a). In Fig. 3b, we show the 20 families that have undergone the largest expansions.

Among the gene families showing varying degrees of expansion, there are a number that play vital roles in spiders’ survival, including those related to immunity, dietary digestion, and detoxification. The expansion of immunity-related gene families, such as putative peptidases, immunoglobulin I-set domain, and retroviral aspartyl proteases, reflects the powerful innate immune response of spiders [12, 13], whereas certain digestion- and detoxification-related gene families, such as cytochrome P450s, peptidases, and proteases, may reflect mechanisms underlying the wide dietary repertoire of the spider *T. antipodiana*. For example, members of the cytochrome P450 family play important roles in digestion and detoxification by contributing to xenobiotic metabolism and insecticide resistance [70]. Given its large webs and diverse range of prey items, it is essential for *T. antipodiana* to have effective digestion and detoxification systems, and GO and KEGG pathway enrichment analyses of these expanded genes further confirmed this hypothesis.

**Figure 4:**
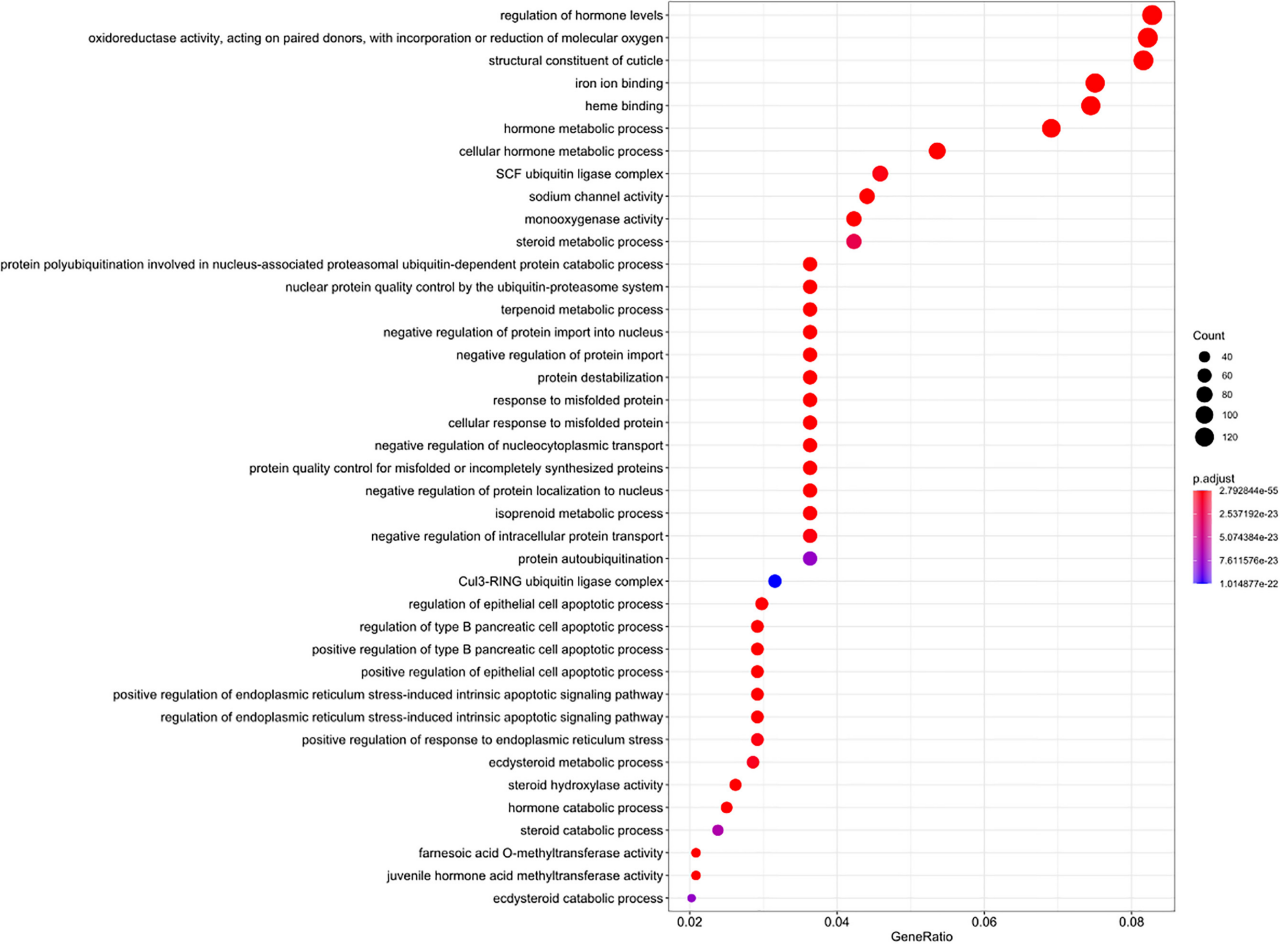
GO annotation of the expanded gene families.

Among the GO enrichment results, we noted certain important functions associated with the regulation of hormone levels, oxidoreductase activity, structural constituent of the cuticle, and metabolic and catabolic processes (including hormone, steroid, isoprenoid, and ecdysteroid metabolic processes). The enrichment of these metabolic and catabolic processes is again consistent with the strong detoxification ability of *T. antipodiana* (Fig. 4).

**Figure 5:**
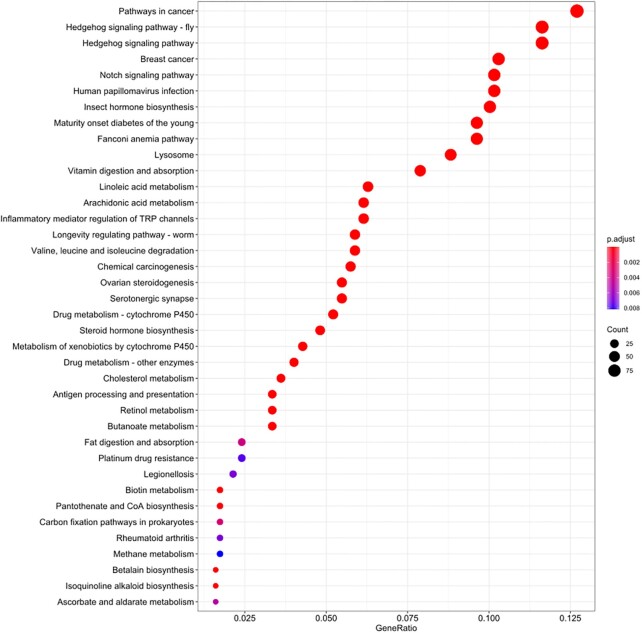
KEGG annotation of the expanded gene families.

Among the KEGG enrichment results (Fig. 5), we identified a number of important functions, including cell proliferation and differentiation (such as cancer-related, hedgehog signaling, and notch signaling pathways), biosynthesis, and metabolism (such as linoleic, arachidonic, and drugs) that are consistent with the GO enrichment results. We also detected strong enrichment of drug and xenobiotic metabolism by cytochrome P450.

### Analysis of detoxification-related gene families in *T. antipodiana*

Numerous families of genes, including P450s, GSTs, ABCs, and CCEs, play roles in the detoxification of toxic compounds, and these genes have most likely evolved in relation to polyphagous species (Table 5). In the polyphagous species (e.g., spider mite *T. urticae, Spodoptera frugiperda, Tribolium castaneum, Spodoptera litura, Helicoverpa armigera*, and *Trialeurodes vaporariorum*), the number of these genes showed a great expansion [78–84], while in monophagous or oligophagous species (e.g., *B. mori, Pediculus humanus humanus*), the expansions of these gene families are rarely observed [78, 84–86]. For further analysis of the detoxification ability of *T. antipodiana*, we manually annotated the genes of detoxification-related enzymes (P450s, CCEs, GSTs, and ABCs).

**Table 5:** Counts of proteins associated with detoxification enzymes in *Trichonephila antipodiana* and other Arthropods

Species	Type	P450s	ABCs	CCEs	GSTs	Reference
*Trichonephila antipodiana*	Polyphagous	167	48	48	22	This study
*Spodoptera frugiperda*	Polyphagous	425	58	NA	29	[81]
*Tribolium castaneum*	Polyphagous	128	73	60	35	[82]
*Spodoptera litura*	Polyphagous	138	54	NA	47	[83]
*Helicoverpa armigera*	Polyphagous	114	54	97	42	[84]
*Tetranycbus urticae*	Polyphagous	81	103	71	31	[78,79]
*Trialeurodes vaporariorum*	Polyphagous	80	46	31	26	[82]
*Manduca sexta*	Oligophagous	103	54	96	31	[84]
*Bombyx mori*	Monophagous	83	51	69	26	[78, 85]
*Pediculus humanus humanus*	Monophagous	37	40	NA	13	[86]

NA: lack of data or no reference.

From the perspective of xenobiotic metabolism, P450s are the most important superfamily of enzymes in arthropods [87]. In the genome of *T. antipodiana*, we identified 167 CYP genes, comprising 4 major classes: CYP2 (57 genes), mitochondrial P450 (19), CYP3 (43), and CYP4 (48). Among insects, the numbers of P450 genes to some extent reflect adaptation and pesticide resistance (Table 5). For example, in some polyphagous species such as the red flour beetle, *Tribolium castaneum* (Coleoptera), and 3 moths, *S. litura, S. frugiperda*, and *H. armigera* (Lepidoptera), the number of P450 genes shows a great expansion, with 143, 138, 425, and 114 genes identified, respectively. In contrast, in some monophagous or oligophagous species, these expansions are rarely observed, such as in*B. mori* (Lepidoptera) and *P. humanus humanus*for the number of P450 genes of 83 and 37.

**Figure 6:**
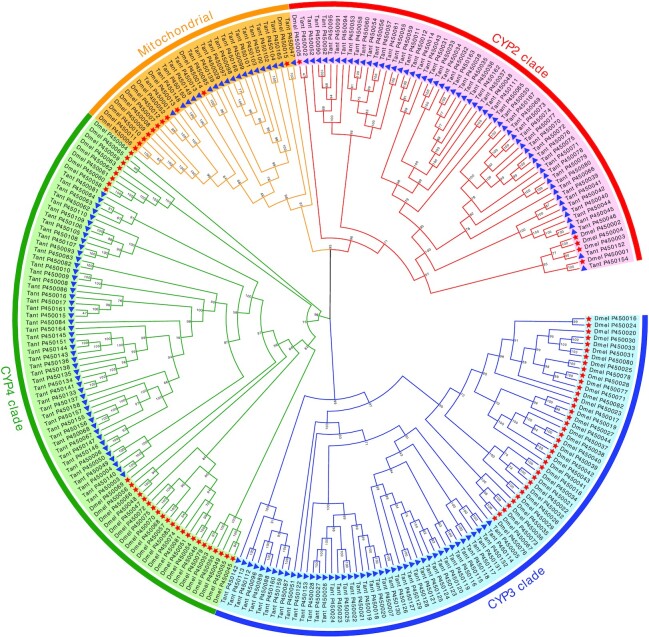
Expansion of the P450 gene family in *Trichonephila antipodiana*. The phylogenetic tree shows the orthologous and paralogous relationships of all P450 genes from *T. antipodiana* and *Drosophila melanogaster*. Bootstrap values are indicated on the nodes.

Compared with other arthropods, the number of genes of every class in commonly used model species, such as *D. melanogaster*, shows varying degrees of increase (Fig. 6). We can see that among the CYP genes of *T. antipodiana*, CYP2 clade genes showed a remarkable expansion. CYP2 enzymes are associated with detoxification and/or bioactivation of certain foreign chemicals [87]. Similar results have been obtained for the polyphagous species *T. urticae*, revealing 81 CYP genes with a notable lineage-specific expansion of duplicated intron-less CYP2 clade genes [79]. With regards to *T. antipodiana*, it is conceivable that the expansion of the CYP2 clade may be associated with its polyphagous habit.

In these polyphagous species of Coleoptera and Lepidoptera, the CYP3 and CYP4 clade genes of P450 showed expansion (Table 5). In addition, the number of genes in the CYP3 and CYP4 clades in *T. antipodiana* also showed a great expansion. The CYP3 clade genes have been found to be associated with xenobiotic metabolism and insecticide resistance when induced by phenobarbital, pesticides, or natural products, whereas certain clade CYP4 genes, the least studied among the insect CYP genes, can be induced by xenobiotics as metabolizers, and others are linked to odorant or pheromone metabolism. In insects, it has been reported that the mitochondrial P450 clade is associated with insecticide resistance [87]; e.g., the CYP12A1 gene of the housefly has been shown to play a role in the metabolism of xenobiotics, although not insect ecdysteroids. Moreover, it has been reported that exposure to cadmium increases expression of cytochrome P450-encoding genes in the wolf spider *Pirata subpiraticus* [88].

**Figure 7:**
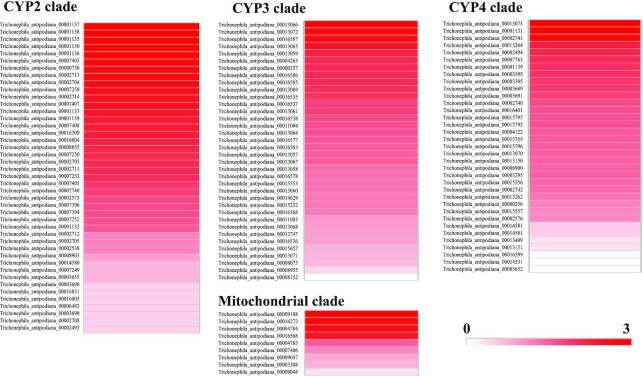
The heat map of P450 gene expression in the female *T. antipodiana* by RNA-Seq.

In addition, inducing changes in the expression of detoxification-related genes provides polyphagous arthropods greater fitness on a specific host. For example, if *T. urticae* changes from its optimal host (bean) to a challenging host (tomato), transcriptional responses increase with widespread changes [89]. We also analyzed P450 gene expression in the female *T. antipodiana* by means of RNA-Seq, and the expression patterns are shown in Fig. 7.

The CCE superfamily comprises a functionally diverse group of proteins that hydrolyze carboxylicesters [21]. CCEs not only regulate endogenous compounds (such as hormones, pheromones, and acetylcholine) but also detoxify exogenous compounds derived from dietary or environmental sources. These genes have been categorized into 3 main phylogenetic classes, namely, hormone/semiochemical processing, dietary/detoxification, and neuro/developmental functions. Within the *T. antipodiana* genome, we identified 48 CCE genes, among which almost all (47) belong to the neuro/developmental class, with the single remaining gene belonging to the hormone/semiochemical class (Supplementary Fig. S2). Notably, whereas in the fruit fly *D. melanogaster*, the number of CCEs in the neuro/developmental class is relatively conserved, we detected a clear expansion in the *T. antipodiana* genome (Supplementary Fig. S2), thereby reflecting the difference between spiders and insects.

GSTs play roles in cellular detoxification by catalyzing nucleophilic attack of the tripeptide glutathione in the electrophilic centers of xenobiotic and endobiotic compounds [90]. Within the *T. antipodiana* genome, we identified 22 GST genes, and phylogenetic analyses of the cytosolic *T. antipodiana* GSTs revealed 5 different classes of these genes (Supplementary Fig. S3), namely, Delta/Epsilon (2 genes), Mu (15), Theta (1), Sigma (2), and Zeta (2), among which the Mu class is the largest and shows considerable expansion in *T. antipodiana*. Functionally, the Mu GSTs have been reported to participate in the oxidative stress response–associated pesticide resistance in *T. urticae* [91].

The ABCs can act directly on toxicants as primary-active transporters, thereby protecting cells or organisms [23]. The genome of *T. antipodiana* was found to contain 48 ABC genes belonging to 8 different classes (Supplementary Fig. S4): ABCA (11 genes), ABCB (12), ABCC (11), ABCD (3), ABCE (1), ABCF (3), ABCG (6), and ABCH (1). Among the annotated genomes of arthropod species that have been studied in detail, that of *T. urticae* has been found to contain the largest number of ABC genes (103), followed by those of *T. castaneum* (73) and *D. pulex* (65), whereas the genome of *A. mellifera* has only 41 ABC genes.

### Analysis of the *T. antipodiana* genome provides evidence in support of a WGD event

On the basis of our analysis of the *T. antipodiana* genome, we provide 2 lines of evidence in support of the hypothesis that an ancient WGD probably occurred after the divergence of the common ancestor of spiders and scorpions from other arachnid lineages (mites, ticks, and harvestmen) prior to 430 Mya [92–94], which occurred independently of the apparent WGD that is evident in all extant horseshoe crabs [95, 96].

First, synteny analysis revealed the occurrence of certain segmental duplications, the signatures of which are suggestive of a WGD. These signatures were observed in multiple chromosomes, such as chromosomes 2, 3, 9, and 10 (Fig. 1). These results are comparable with the findings of a similar analysis of the *P. tepidariorum* genome [97]. The conservation of synteny within the genome of *T. antipodiana* supports the hypothesis of a WGD event.

In addition, we detected 2 clusters of Hox genes. Variation in the number of Hox gene clusters is considered to be consistent with the occurrence of WGD events during the course of evolution [68]. In the present study, we identified Hox genes of the following classes in the *T. antipodiana* genome: *lab, pb, Hox3, Dfd, Scr, ftz, Antp, Ubx, abdA*, and *AbdB*. One complete HOX cluster copy was identified on chromosome 12, whereas a further HOX cluster detected on in chromosome 8 was found to be lacking copies of *Hox3, ftz, ubx*, and *Abd-a* genes (Fig. 2). Notably, however, we detected 2 copies of nearly all the Hox genes in the *T. antipodiana* genome, thereby indicating that entire Hox clusters have been duplicated. The results are consistent with those obtained in a previous study on the house spider *P. tepidariorum* [7].

## Conclusion

A high-quality chromosome-level genome for the spider *Trichonephila antipodiana* was assembled, which is the second chromosome-level spider genome to date. The polyphagy of this species is highly related to the P450 gene families. The large-scale inter-chromosomal duplications and duplicated clusters of Hox genes highlight the WGD event during the evolution of spiders. The high-quality genome assembled here provides more useful data for studies on the evolutionary adaptations of spiders and species-specific functions.

## Availability of Supporting Data and Materials

All raw sequencing data and the genome assembly of *T. antipodiana* underlying this article are available at the NCBI and can be accessed with Bioproject ID PRJNA627506. Other data supporting this work are openly available in the *GigaScience* repository, GigaDB [98].

## Additional Files

**Figure S1**.*k*-mer distribution of the *Trichonephila antipodiana* genome.

**Figure S2**. Expansion of the CCE gene family in *Trichonephila antipodiana*. The phylogenetic tree shows the orthologous and paralogous relationships of all CCE genes from *T. antipodiana* and *Drosophila melanogaster*. Bootstrap values are indicated on the nodes.

**Figure S3**. Expansion of the GST gene family in *Trichonephila antipodiana*. The phylogenetic tree shows the orthologous and paralogous relationships of all GST genes from *T. antipodiana* and *Drosophila melanogaster*. Bootstrap values are indicated on the nodes.

**Figure S4**. Expansion of the ABC gene family in *Trichonephila antipodiana*. The phylogenetic tree shows the orthologous and paralogous relationships of all ABC genes from *T. antipodiana* and *D. melanogaster*. Bootstrap values are indicated on the nodes.

## Abbreviations

ABC: ATP-binding cassette transporters; ATP: adenosine triphosphate; bp: base pairs; BLAST: Basic Local Alignment Search Tool; BUSCO: Benchmarking Universal Single-Copy Orthologs; CCE: carboxyl/cholinesterases; COG: clusters of orthologous groups; EC: expression coherence; Gb: gigabase pairs; GO: Gene Ontology; GST: glutathione-*S*-transferases; Hi-C: high-throughput chromosome conformation capture; Hox genes: homeotic genes; kb: kilobase pairs; KDE: kernel density estimate; KEGG: Kyoto Encyclopedia of Genes and Genomes; Kos: KEGG orthologous groups; Ks: pairwise synonymous substitution rates; LINE: long interspersed nuclear element; LTR: long terminal repeat; MAFFT: Multiple Alignment using Fast Fourier Transform; Mb: megabase pairs; mya: million years ago; ncRNA: non-coding RNA; P450: P450 mono-oxygenase; PacBio: Pacific Biosciences; SINE: short interspersed nuclear element; TE: transposable element; tRNA: transfer RNA; WGD: whole-genome duplication.

## Competing Interests

The authors declare that they have no competing interests.

## Authors’ Contributions

Z.F. performed the major part of data analysis and drafted the manuscript. L.Y.W., T.Y., and P.L. contributed to sample collection. J.F.J. and F.Z. contributed to data analysis and edits to the manuscript. Z.S.Z. contributed to research design and final edits to the manuscript. All authors read and approved the final manuscript.

## Supplementary Material

giab016_GIGA-D-20-00316_Original_Submission

giab016_GIGA-D-20-00316_Revision_1

giab016_Response_to_Reviewer_Comments_Original_Submission

giab016_Reviewer_1_Report_Original_Submission Prashant Sharma -- 12/7/2020 Reviewed

giab016_Reviewer_1_Report_Revision_1 Prashant Sharma -- 2/1/2021 Reviewed

giab016_Reviewer_2_Report_Original_Submission Jesper Bechsgaard -- 12/10/2020 Reviewed

giab016_Reviewer_2_Report_Revision_1 Jesper Bechsgaard -- 2/1/2021 Reviewed

giab016_Supplemental_Files
