# Supplementary material for: A chromosome‐level genome of the spider Trichonephila antipodiana reveals the genetic basis of its polyphagy and evidence of an ancient whole-genome duplication event
Source: Gigascience. 2021 Mar 19;10(3):giab016. doi: 10.1093/gigascience/giab016 (PMC7976613; doi:10.1093/gigascience/giab016)
Supplement: giab016_GIGA-D-20-00316_Original_Submission [file giab016_giga-d-20-00316_original_submission.pdf]

GigaScience

A chromosome-level genome of the spider Trichonephila antipodiana reveals the genetic basis of its polyphagy and evidence of an ancient whole-genome duplication event

--Manuscript Draft--

Manuscript Number:	GIGA-D-20-00316
Full Title:	A chromosome-level genome of the spider Trichonephila antipodiana reveals the genetic basis of its polyphagy and evidence of an ancient whole-genome duplication event
Article Type:	Data Note
Funding Information:	the key Natural Science Foundation of Chongqing (cstc2019jcyj-zdxmX0006) — Mr Zhisheng Zhang<br>the Investigation Project of Basic Science and Technology (2018FY100305) — Not applicable
Abstract:	Background : The spider Trichonephila antipodiana (Araneidae), commonly known as the batik golden web spider, preys on arthropods with body sizes ranging from approximately 2 mm in length to insects larger than itself (over 20 – 50 mm), indicating its polyphagy and strong dietary detoxification abilities. Although it has been reported that an ancient whole-genome duplication event occurred in spiders, lack of a high-quality genome has limited characterization of this event.<br>Results: We present a chromosome-level T. antipodiana genome constructed based on PacBio and Hi-C sequencing. The assembled genome is 2.29 Gb in size with a scaffold N50 of 172.89 Mb. Hi-C scaffolding assigned 98.5% of the bases to 13 pseudo-chromosomes, and BUSCO completeness analysis revealed that the assembly included 94.8% of the complete arthropod universal single-copy orthologs (n=1,066). Repetitive elements account for 59.21% of the genome. We predicted 19,001 protein-coding genes, of which 96.78% were supported by transcriptome-based evidence and 96.32% matched protein records in the UniProt database. The genome also shows substantial expansions in several detoxification -associated gene families, including cytochrome P450 monooxygenases, carboxyl/cholinesterases, glutathione- S -transferases, and ATP-binding cassette transporters, reflecting the possible genomic basis of polyphagy. Further analysis of the T. antipodiana genome architecture revealed an ancien t whole-genome duplication event, based on three lines of evidence: (1) large-scale duplications from inter-chromosome synteny analysis; (2) unimodal distribution between 1.5 and 2.0 from synonymous substitution rate distribution analysis; and (3) duplicated clusters of Hox genes.<br>Conclusions: The high-quality T. antipodiana genome represents a valuable resource for spider research and provides insights into this species' adaptation to the environment.
Corresponding Author:	Zhisheng Zhang, Ph.D<br>Southwest University<br>Chongqing, CHINA
Corresponding Author Secondary Information:	
Corresponding Author's Institution:	Southwest University
Corresponding Author's Secondary Institution:	
First Author:	Zheng Fan
First Author Secondary Information:	
Order of Authors:	Zheng Fan
	Tao Yuan
	Piao Liu
	Luyu Wang

Powered by Editorial Manager® and ProduXion Manager® from Aries Systems Corporation

	Jianfeng Jin
	Feng Zhang
	Zhisheng Zhang, Ph.D
**Order of Authors Secondary Information:**	
**Additional Information:**	
**Question**	**Response**
Are you submitting this manuscript to a special series or article collection?	No
**Experimental design and statistics**<br><br>Full details of the experimental design and statistical methods used should be given in the Methods section, as detailed in our Minimum Standards Reporting Checklist. Information essential to interpreting the data presented should be made available in the figure legends.<br><br>Have you included all the information requested in your manuscript?	Yes
**Resources**<br><br>A description of all resources used, including antibodies, cell lines, animals and software tools, with enough information to allow them to be uniquely identified, should be included in the Methods section. Authors are strongly encouraged to cite Research Resource Identifiers (RRIDs) for antibodies, model organisms and tools, where possible.<br><br>Have you included the information requested as detailed in our Minimum Standards Reporting Checklist?	Yes
**Availability of data and materials**<br><br>All datasets and code on which the conclusions of the paper rely must be either included in your submission or deposited in publicly available repositories (where available and ethically	Yes

appropriate), referencing such data using a unique identifier in the references and in the “Availability of Data and Materials” section of your manuscript.<br><br>Have you have met the above requirement as detailed in our Minimum Standards Reporting Checklist?	

Manuscript

Click here to access/download;Manuscript;renamed_e8843.docx

A chromosome- level genome of the spider *Trichonephila antipodiana* reveals the genetic basis of its polyphagy and evidence of an ancient whole-genome duplication event

Zheng Fan[1], Tao Yuan[1], Piao Liu[1], Luyu Wang[1], Jianfeng Jin[2], Feng Zhang[2], Zhisheng Zhang[1*]

1. School of Life Sciences, Southwest University, Chongqing 400700, China.
2. Department of Entomology, College of Plant Protection, Nanjing Agricultural University, Nanjing, Jiangsu 210000, China

* Correspondence should be addressed to Z.Z. (zhangzs327@qq.com)

Abstract

Background: The spider *Trichonephila antipodiana* (Araneidae), commonly known as the batik golden web spider, preys on arthropods with body sizes ranging from approximately 2 mm in length to insects larger than itself (over 20–50 mm), indicating its polyphagy and strong dietary detoxification abilities. Although it has been reported that an ancient whole-genome duplication event occurred in spiders, lack of a high-quality genome has limited characterization of this event.

Results: We present a chromosome- level *T. antipodiana* genome constructed based on PacBio and Hi-C sequencing. The assembled genome is 2.29 Gb in size with a scaffold N50 of 172.89 Mb. Hi- C scaffolding assigned 98.5% of the bases to 13 pseudo-chromosomes, and BUSCO completeness analysis revealed that the assembly included 94.8% of the complete arthropod universal single-copy orthologs (n=1,066). Repetitive elements account for 59.21% of the genome. We predicted 19,001 protein-coding genes, of which 96.78% were supported by transcriptome-based evidence and 96.32% matched protein records in the UniProt database.

The genome also shows substantial expansions in several detoxification-associated gene families, including cytochrome P450 monooxygenases, carboxyl/cholinesterases, glutathione-*S*-transferases, and ATP-binding cassette transporters, reflecting the possible genomic basis of polyphagy. Further analysis of the *T. antipodiana* genome architecture revealed an ancient whole-genome duplication event, based on three lines of evidence: (1) large-scale duplications from inter-chromosome synteny analysis; (2) unimodal distribution between 1.5 and 2.0 from synonymous substitution rate distribution analysis; and (3) duplicated clusters of Hox genes.

Conclusions: The high-quality *T. antipodiana* genome represents a valuable resource for spider research and provides insights into this species' adaptation to the environment.

Keywords Hi-C, high- quality genome, whole-genome duplication, gene family analysis, cytochrome P450, ABC, CCE, GST, Hox

Data Description

Background

Spiders (Araneae) have a worldwide distribution, have conquered virtually all ecological environments, and exhibit considerable species richness. A total of 48,683 spider species have been described to date, classified into 4,175 genera and 128 families [1]. Spiders are notable with respect to their numerous distinctive characteristics, including the production of silk [2] and venom [3], prolonged milk provisioning [4], foraging behavior [5], sexual size dimorphism [6], and whole-genome duplications (WGDs) [7]. To date, the genomes of 11 species of spider have been published or are available in the NCBI database (Table 1), which offer unprecedented

insights into the unique biology of these arthropods [8]. Complex sets of venom and silk genes have been identified in the genomes of *Stegodyphus mimosarum*, *Acanthoscurria geniculate*, and *Trichonephila clavipes* (formerly *Nephila clavipes*) [9,10]. Furthermore, the role of DNA methylation in spider gene regulation has been demonstrated in the genome of *Stegodyphus dumicola* [11], whereas components of the spider immune system were initially characterized with reference to the genome of *Parasteatoda tepidariorum* [12]. WGD is a process of genome doubling that supplies raw genetic material and increases genome complexity. It can provide new genetic material that enables paralogous genes to undergo sub- or neo-functionalization, which can contribute to the rewiring of gene regulatory networks, morphological innovations, and, ultimately, organismal diversification. It has been reported that a WGD event occurred in the ancestor of scorpions and spiders. In spiders, the first evidence of a duplication event was detected in the genome of the house spider *P. tepidariorum*, as indicated by a high number of duplicated genes, including two clusters of Hox genes [13]. However, spider genomes tend to be difficult to sequence, assemble, and annotate owing to their large size and high heterozygosity and repeat content. To date, the genomes of only two species (*Dysdera silvatica* and *Argiope bruennichi*) have been sequenced based on long sequencing reads (PaciBio or Nanopore), only one of which was assembled to the chromosome level [14,15].

In this study, we sequenced the whole genome of the batik golden web spider, *Trichonephila antipodiana*, one of the typical Nephilinae species in the family Araneidae, which is recorded from a number of countries, including Australia (Queensland), the Solomon Islands, New Guinea, The Philippines, and China (Hainan Island) [1, 16]. Recently, in addition to many taxonomic papers that have provided a clear outline of species in the Nephilinae,

numerous studies on this subfamily have focused on their silk characteristics and sexual size dimorphism [6, 17, 18]. The webs constructed by *T. antipodiana* are approximately 1.0 m in diameter and can deal with a large size range of any suitable prey, including various species of Araneae, Crustacea, Formicidae, Isoptera, Orthoptera, Diptera, Coleoptera, Lepidoptera, Hymenoptera, Odonata, and even small birds, which thereby indicates their polyphagy and strong detoxification abilities [16]. Furthermore, it has been reported that when recycling their orb webs, these spiders may also feed on adhering pollen grains or fungal spores via extraoral digestion [19].

In this study, we combined Pacific Biosciences (PacBio) and high-throughput chromosome conformation capture (Hi-C) sequencing to produce a high-quality, chromosome-level reference genome for *T. antipodiana*. We describe the salient features of the *T. antipodiana* genome, focusing on genome assembly, annotation, and evolutionary analyses. To investigate the polyphagy and detoxification of this spider, we analyzed a selection of detoxification-associated gene families, including P450 monooxygenases, carboxyl/cholinesterases (CCE), glutathione-*S*-transferases (GST), and ATP-binding cassette transporters (ABC). Furthermore, to gain evidence in support of a spider WGD event, we performed synteny, synonymous substitution rate distribution, and Hox gene analyses.

The *T. antipodiana* reference genome described herein will lay a foundation for further research on the unique characteristics and functions of spiders.

Methods

Sample collection and sequencing

The female specimen of *T. antipodiana* used in this experiment was obtained from Beihai (Guangxi Province, China), which was stored at -80°C prior to DNA extraction. The spider, excluding the abdomen, was prepared for PacBio and Illumina whole-genome sequencing, and leg muscle tissue was used for Illumina transcriptome sequencing.

Genome sequencing was performed by Berry Genomics (Beijing, China). Genome DNA for PacBio and Illumina sequencing was isolated using a Qiagen Blood & Cell Culture DNA Mini Kit. PacBio Sequel II libraries for PacBio sequencing were constructed with insert sizes of 20 kb using a SMRTbell™ Template Prep Kit 1.0-SPv3. Two short paired-end insert libraries containing 350-bp sequences were constructed for survey analysis using a Truseq DNA PCR-free kit and sequenced using the NovaSeq 6000 platform.

For the purposes of Hi-C sequencing, the muscle tissues of the single female specimen were fixed with formaldehyde and lysed, and the cross-linked DNA was subsequently digested overnight with *Mbo*I. Sticky ends were biotinylated and proximity-ligated to form chimeric junctions that were enriched for and then physically sheared to a size of 350 bp. Chimeric fragments representing the original cross-linked long-distance physical interactions were then processed into paired-end sequencing libraries, and 150-bp paired-end reads were generated using the Illumina HiSeq PE150 platform.

Muscle RNA was extracted using TRIzol (Invitrogen) according to the manufacturer's instructions.

Genome survey and assembly

Quality control of the raw Illumina data was performed using BBTools suite v38.67 (Bestus

Bioinformaticus Tools, RRID:SCR_016968) [20]. The duplicates were removed using "clumpify.sh," and then "bbduk.sh" was used to trim the reads ends to Q20 with reads shorter than 15 bp or with >5 Ns. The poly-A/G/C tails of at last 10 bp were trimmed, and the overlapping paired reads were corrected using "bbduk.sh." All filtered reads were used to estimate genome size and other characteristics. In addition, a 21-mer was selected for k-mer analysis and the k-mer distribution was estimated using "khist.sh" (BBTool). The 21-mer depth frequency distribution was calculated using GenomeScope v1.0.0 (GenomeScope, RRID:SCR_017014) [21], and the maximum k-mer coverage cutoff was set to 10,000.

For the long reads generated using the PacBio Sequel platform, contig assembly of the *T. antipodiana* genome was conducted using Flye v2.5 (Flye, RRID:SCR_017016) [22] with a single round of polishing and the minimum overlap between reads was set to 3,000. Heterozygous regions of the assembly were removed using Purge Haplotigs v1.1.0 [23], with a 50% cutoff for identifying contigs as haplotigs. Illumina reads were used to polish the assembly using NextPolish v1.0.5 [24] over two rounds. During all the Flye and NextPolish polishing steps, Minimap2 v2.12 (Minimap2, RRID:SCR_018550) [25] was used as the read aligner.

The Hi-C reads were used to generate a chromosome-level assembly of the genome, and three software packages were used for analysis. The reads were initially subjected to quality control to removing the duplicates and then aligned to the genome using Juicer v1.6.2 (Juicer, RRID:SCR_017226) [26]. The resulting alignment BAM file was then transformed to a BED format and fed to SALSA v2.2 [27] to correct the obvious misjoin errors between contigs. The alignment BAM file was also mapped to the cleaned assembly data using Minimap2. Finally, the data were fed to Allhic v0.9.13 [28] to anchor contigs to chromosomes.

Potential contaminant sequences were inspected using HS-BLASTN [29] and BLAST+ (blastn) v2.7.1 [30] against the NCBI nucleotide (nt) and UniVec databases.

Genome completeness was assessed using the Benchmarking Universal Single-Copy Orthologs (BUSCO) v3.0.2 pipeline (BUSCO v3.0, RRID: SCR_015008) [31] against an arthropod reference gene set using the arthropoda_odb 9 database of the genome (n = 1,066). To evaluate the mapping rate, the clean reads of the Illumina or PacBio sequences were mapped to the reference genome using Minimap2.

Genome annotation

Genome annotation essentially encompasses four aspects: repeat, protein-coding gene, non-coding RNA, and gene function annotations.

We searched for repetitive elements in the assembled genome by a combination of ab-initio and homology-based searching. Initially, we constructed a specific repeat database using RepeatModeler v2.0.1 (RepeatModeler, RRID:SCR_015027)) [32], and thereafter combined the an-initio database and known repeat library (Repbase) [33] as the reference repeat database. To identify repetitive elements, we used RepeatMasker (RepeatMasker, RRID:SCR_012954) [34] to search against the reference repeat database. Non-coding RNAs were identified using Infernal v1.1.2 (Infernal, RRID:SCR_011809) [35] and tRNAscan-SE v2.0.6 (tRNAscan-SE, RRID:SCR_010835) [36], and tRNAs of high confidence were confirmed using the tRNAscan-SE script “EukHighConfidenceFilter”.

Using the repeat-masked genome, we used Maker v2.31.10 (Maker, RRID:SCR_005309) for genome annotation by integrating ab initio, transcriptome-based, and protein homology-

based evidence [37]. Augustus v3.3.2 (AUGUSTUS, RRID:SCR_008417) [38] and GeneMark-ES/ET/EP v4.48_3.60_lic [39] were used for ab-initio gene prediction. To accurately model the sequence properties, both gene finders were initially trained using the BRAKER v2.1.5 pipeline (BRAKER, RRID:SCR_018964) [40], which makes use of the mapped transcriptome sequence data. Previously, RNA-seq data were mapped to our genome assembly using HISAT2 v 2.2.0 (HiSat2, RRID:SCR_015530) [41]. BRAKER was then run with default parameters. The RNA-seq data were further assembled into transcripts using Stringtie v2.1.3 [42], with the assembled genome used as a reference. The resulting transcripts were provided as input for Maker via the "est" option. The protein sequences of *Drosophila melanogaster* (GCA_000001215.4), *Ixodes scapularis* (GCA_002892825.2), *Stegodyphus mimosarum* (GCA_000611955.2), *Trichonephila clavipes* (GCA_002102615.1), *Parasteatoda tepidariorum* (GCA_000365465.3), *Strigamia maritima* (GCA_000239455.1), and *Daphnia pulex* (GCA_900092285.2) were downloaded from the NCBI database as protein homology-based evidence required by Maker.

The functions of the predicted protein sequences were assigned against the UniProtKB/Swissprot database using Diamond v0.9.24 (Diamond, RRID:SCR_016071) [43] with a more sensitive mode, one maximum number of target sequences, to report alignments with an e-value threshold of 1e-5.

Annotation of the protein domains was based on Gene Ontology (GO) and Reactome pathways of the predicted protein-coding genes, with InterProScan v5.41-78.0 (InterProScan, RRID: SCR_005829) [44] being used to screen proteins against the following five databases: Pfam [45], Panther [46], Gene3D [47], Superfamily [48], and CDD [49].

Using eggNOG-mapper v2.0 [50], the eggNOG v5.0 database [51] was used for GO, EC (expression coherence), KEGG (Kyoto Encyclopedia of Genes and Genomes) pathways, KEGG orthologous groups (KOs), and COG (clusters of orthologous groups) functional category annotation of the predicted protein-coding genes.

To assess the completeness of the *T. antipodiana* protein annotation, we used the protein mode of the BUSCO v3.0.2 ((RRID:SCR_015008) pipeline and the arthropod reference set of arthropoda_odb 9 (n = 1,066) [31].

Phylogenetic analyses and GO/KEGG enrichment analyses

Orthologous gene clusters were classified using OrthoFinder v2.3.8 (OrthoFinder, RRID:SCR 017118) [52] across the well-annotated and well-assembled genomes of 10 species covering representative Chelicerata lineages along with *T. antipodiana*: one Scorpiones (*Centruroides sculpturatus*, GCA_000671375.2); five Acari (*Dermatophagoides pteronyssinus*, GCA_001901225.2; *Galendromus occidentalis*, GCA_000255335.1; *Tetranychus urticae*, GCA_000239435.1; *Varroa destructor* GCA_002443255.1; *I. scapularis*, GCA_002892825.2); three Araneae (*Parasteatoda tepidariorum*, GCA_000365465.3; *Stegodyphus mimosarum*, GCA_000611955.2; *T. clavipes* GCA_002102615.1); and one Xiphosura (*Tachypleus tridentatus*). With the exception of *T. tridentatus* (doi:10.5061/dryad.68pk1rv), most protein sequences were downloaded from the NCBI database.

To infer the phylogeny of these species, the protein sequences of 236 single-copy genes were separately aligned using MAFFT v7.394 (MAFFT, RRID:SCR_011811) [53] based on the L-INS-I strategy. The resulting alignments were trimmed using trimAl v1.4.1 (trimAl,

RRID:SCR_017334) [54] to remove sites of unclear homology using the heuristic method "automated1." The resulting alignments were concatenated using FASconCAT-G v1.04 [55]. Genes that violated the models were removed prior to tree inference. Finally, ML reconstructions were performed using IQ-TREE v2.0.7 (IQ-TREE, RRID:SCR_017254) [56] with extended model selection followed by tree inference, model set by LG, with the number of partition pairs for the rcluster algorithm, replicates for ultrafast bootstrap, and SH approximate likelihood ratio tests being 1000, 10, and 1000, respectively.

The divergence time was estimated with MCMCTree within the package PAML v4.9j (PAML, RRID:SCR_014932) [57] using parameters with independent clock rates; BDparas-related birth, death, and sampling rates of 1, 10, and 0.1, respectively; kappa_gamma of 62; alpha_gamma of 11; rgene_gamma of 2201; and sigma2_gamma of 1101. Fossil records were derived from the paleobiodb database (https://paleobiodb.org/), with Chelicerata (genus *Paleomerus*, 516–541 Mya), Araneae (genus *Arthrolycosa*, 315.2–323.2 Mya), and Arachnida (Acariformes, *Pseudoprotacarus scoticus*, 407.6–419.2 Mya) being used for time calibration.

Café v4.2.1 (CAFÉ, RRID:SCR_005983) [58] was employed to identify the likelihood of gene family expansion and contraction using the single birth–death parameter lambda and a P-value threshold of 0.01. GO and KEGG functional enrichment of the significantly expanded families was assessed using Tbtools v1.045 [59].

Annotation of dietary detoxification-related gene families

To manually annotate the genes of detoxification-related enzymes (P450s, CCEs, GSTs, and ABCs), we initially downloaded the amino acid sequences of the P450s, CCEs, GSTs, and

ABCs predicted from the *D. melanogaster*, *Bombyx mori*, and *T. urticae* sequences obtained from NCBI.

For cytochrome P450 proteins, we performed a blastp-like search using MMsesqs2 v11 [60] with four rounds of iteration, as the identity between two proteins can be as low as 25%. Using the Pfam database, Interproscan v5.41-78.0 (Interproscan, RRID:SCR_005829) [61] was used to confirm specific conserved domains of the P450 sequences. And every P450 protein was checked the struction including four-helix bundles (D, E, I and L), helices J and K, two sets of β sheets, and a coil referred to as the “meander”. The regions comprise a heme-binding loop, a strictly conserved Glu-X-X-Arg motif in helix K, and a consensus sequence (Ala/Gly-Gly-X-Asp/Glu-Thr-Thr/Ser) in the central part of helix I [62]. We deleted the invalid matches of the proteins using mmseq2 with a tblatn-like search, and each protein was also examined to identify intron/exon boundaries.

Members of the other three detoxification enzyme gene families (CCEs, GSTs, and ABCs) of *T. antipodiana* were identified using MMsesqs2 v11 [60] using a blastp-like search with four rounds of iteration and an e-value of 0.001. Interproscan v5.41-78.0 (Interproscan, RRID:SCR_005829) [61] was used to confirm the specific conserved domains of genes using the Pfam database. Classification and functional categories of the resulting HMMER-Pfam bellow were further checked using an online NCBI BLASTP of the non-redundant (nr) GenBank protein database. Each protein was assessed for intron/exon boundaries, and extremely short or long sequences were removed. Finally, the multi-hits were reduced to the same gene region and we deleted the invalid matches of the proteins using mmseq2 with a tblatn-like search.

We also conducted an analysis of the sequence evolution of the specific gene families cytochrome P450, CCE, GST, and ABC. Initially, the proteins were aligned using MAFFT v7.450 (MAFFT, RRID:SCR_011811) [53] with common parameters, after which the resulting alignments were trimmed using trimAl v1.4.1 (trimAl, RRID:SCR_017334) [54] to remove the sites with unclear homology based on the heuristic method "automated1." Finally, gene trees were constructed using IQ-TREE v2.0.7 (IQ-TREE, RRID:SCR_017254) [56] with an LG model and 1000 ultrafast bootstrap replicates.

WGD analyses

It has been reported that an ancient WGD event occurred in the spider lineage, and in an attempt to confirm the occurrence of this event, we examined three possible lines of evidence.

First, we conducted an intra-specific analysis of the synteny between *T. antipodiana* chromosomes. *T. antipodiana* proteins were searched against themselves with MMsesqs2 v11 [60] using a blastp-like search with three rounds of iteration and an e-value of 0.001. The blast results and gene annotation GFF3 file were fed to MCScanX [63] with an e-value threshold of 1e-8. A collinear block was defined by a homologous region shared by four or more gene sequences with no rearrangements.

K_S is the synonymous distance or the estimated number of synonymous substitutions per synonymous site, and the whole paranome K_S distribution can reveal ancient WGDs using genomic data [64]. We analyzed the K_S distribution of the *T. antipodiana* genome using the wgd pipeline with the "wgd ksd" sub-command. Analysis of downstream K_S distributions was performed using the "wgd kde" sub-command for fitting kernel density estimates

(KDEs) and the "wgd mix" subcommand for fitting mixtures of log-normal components. Finally, "wgd viz" was used for the visualization of histograms and KDEs.

In metazoans, the 10 highly conserved Hox genes play important roles in fundamental developmental processes [65]. In the present study, we manually annotated the Hox genes of *T. antipodiana*, using the Hox protein amino acid sequences predicted for *Daphnia magna*, *Parasteatoda tepidariorum, Centruroides sculpturatus*, *I. scapularis*, and *D. melanogaster* downloaded from the NCBI database. MMsesqs2 v11 was used to perform a blastp-like search for four rounds of iteration with an e-value of 0.001. The Hox gene clusters classification and functional categories of the resulting BLAST bellow were further assessed using the HomeoDB database [66]. The locations of the Hox genes were further confirmed based on genome annotation, and Hox gene clusters and synteny blocks were plotted across chromosomes using Tbtools [59].

Results and Discussion

A high-quality genome among Araneae

In this study, we constructed a chromosome- level *T. antipodiana* genome based on PacBio and Hi-C sequencing.

Sequencing yielded 767.07 Gb of clean data, comprising 305.96 Gb Illumina (133×), 235.79 Gb PacBio (103×), 215.05 Gb Hi-C (94×), and 10.27 Gb transcriptome reads. The long PacBio subreads had mean N50 lengths of 14.81 kb and 21.19 kb, respectively. The detailed sequencing data are summarized in Table 2.

K-mer analysis indicated that the number of unique k-mers peaked at 21 and predicted a genome assembly size of 2.15 Gb (Figure S1), which is in general agreement with the recent draft genome of *T. clavipes* (2.44 Gb).

Using the Flye assembler, we obtained an initial 2.38 Gb genome assembly with a contig N50 of 1.17 Mb. To enhance the draft assemblies, haplotigs and contig overlaps were removed from the genome. The total length of the assembly was 2.31 Gb, with a contig N50 of 1.23 Mb. Finally, Hi-C data were used for genome scaffolding with a mapping rate of 89.16%, and a high-quality chromosome-level genome assembly of *T. antipodiana* was accordingly obtained with a total length of 2.29 Gb, a contig N50 of 1.14 Mb, and a scaffold N50 of 172.89 Mb (Table 3). The genome of *T. antipodiana* is one of the two chromosome-level genomes obtained for spiders to date, the other being that of *A. bruennichi* [15]. A comparison of the genome assembly obtained in the present study with that of the congeneric species *T. clavipes*, indicated the superior quality of the *T. antipodiana* assembly, with a scaffold N50 of 172 Mb compared with that of 62.96 kb obtained for *T. clavipes* (Table 4).

BUSCO is a tool used to assess the completeness of genome/transcriptome assemblies and annotated proteins based on single-copy orthologs, and the BUSCO results obtained in the present study indicated that 967 (94.8%) of the 1,066 orthologs in a reference arthropod data set (arthropoda_odb9) were labeled as complete in our assembly, which is similar to the value obtained for *T. clavipes* (94.85%). The results of BUSCO analysis at all steps in the assembly of the *T. antipodiana* are shown in Table 3.

The mapping rate, which is defined as the proportion of high-throughput sequencing reads that are uniquely mapped to a reference genome, reflects the accuracy of the assembly, and in

the present study, we obtained mapping rates of 96.78%, 97.23%, and 97.61% for the RNA-seq, Illumina, and PacBio reads, respectively.

Gene annotation

The *T. antipodiana* genome comprises 59.21% repetitive elements, including 57.12% transposable elements (TEs), 0.72% small RNAs, 0.13% satellites, 1.08% simple repeats, and 0.19% low-complexity regions (Table 5). The TEs are predominantly represented by five categories of abundant repeats, unclassified (22.08%), DNA transposon elements (22.42%), long interspersed elements (LINEs, 3.61%), long terminal repeats (LTRs, 3.45%), and short interspersed elements (SINEs, 1.10%). An analysis of the distribution of repetitive elements in the *T. antipodiana* genome, revealed that DNA transposon elements are highly distributed in the genome regions (Figure 1), with TcMar-Tc1 and hAT-Charlie being identified as the most common DNA transposons elements, accounting for 7.18% and 6.19%, respectively. We found that the percentage of DNA transposons elements in *T. antipodiana* is higher than that in some other species of spider, including *Argiope bruennichi* (6.27%), *Trichonephila clavipes* (13.71%), *Araneus ventricosus* (14.45%), *Dysdera silvatica* (19.58%), *Stegodyphus dumicola* (16.17%), *Stegodyphus mimosarum* (18.77%), *Pardosa pseudoannulata* (16.55%), *Loxosceles reclusa* (10.23%), *Anelosimus studiosus* (7.94%), *Latrodectus hesperus* (7.03%), and *Parasteatoda tepidariorum* (6.9%) [15].

Using the MAKER2 genome annotation tool, we identified 19,001 protein-coding genes in the *T. antipodiana* genome, with a mean number of 137,611 exons and 116,383 introns per gene, and mean exon and intron lengths of 247.46 bp and 3.73 kb, respectively. On the basis of

BUSCO analysis, we identified 1,027 (96.3%) complete, 60 (5.6%) duplicated, 14 (1.3%) fragmented, and 25 (2.4%) missing orthologs. Furthermore, we found that a total of 18,303 (96.33%) genes had at least one record in the SwissProt or TrEMBL databases. InterProScan and EggOG analyses identified the protein domains for 14,705 (77.39%) genes, 12,226 GO terms, 9,465 KEGG ko terms, 5,788 KEGG pathways, 14,325 COG categories, and 3,183 Enzyme Codes. Comparatively, 22,689 protein-coding genes have been identified in the *T. clavipes* genome, which approximately comparable to the number in *T. antipodiana* (Figure 2a).

We identified 4,452 ncRNA-associated loci in the squid sequencing data, and found that all the essential and well-conserved metazoan ncRNAs are also present in the *T. antipodiana* genome: 3,653 tRNAs, 160 ribosomal RNAs (rRNAs) (5S, 5.8S, SSU, and LSU), 2 RNase P, 1 RNase MRP, 22 SRP, 216 major spliceosomal snRNAs (U1, U2, U4, U5, U6), 26 minor spliceosomal snRNAs (U11, U12, U4atac, and U6atac), and 6 CD-boxes.

Gene orthology and comparative analysis with other genomes

Identifying homologous relationships among the sequences of different species plays a pivotal role in enhancing our understanding of evolution and diversity. In this regard, we compared the protein-coding genes of *T. antipodiana* with those of 10 representative Arachnida species, including three species of spider (*Parasteatoda tepidariorum*, *Stegodyphus mimosarum*, and *T. clavipes*), one Scorpiones (*Centruroides sculpturatus*), and five Acari (*Dermatophagoides pteronyssinus*, *Galendromus occidentalis*, *Tetranychus urticae*, *Varroa destructor*, and *Ixodes scapularis*) to identify orthologous groups, with *Tachypleus tridentatus* being used as an outgroup. Using OrthoFinder [52], we obtained a total of 203,348 genes among the 11 species,

which were clustered into 20,785 orthogroups. We also identified the genes of single-copy and multi-copy orthologs, common genes unique to Araneae, species-specific genes, and other unassigned orthologous genes among the 11 species (Figure 2a). Gene family analysis also revealed that among these species, 152 gene families and 590 genes were unique to *T. antipodiana*.

To gain an understanding of Arachnida genomic evolution, we reconstructed a phylogenomic tree of the 11 assessed species based on 236 single-copy orthologous genes, which were calibrated using two fossil records. The phylogenomic tree obtained indicated that Scorpiones (*C. sculpturatus*) show a close relationship with spiders, with an estimated time of divergence of the two lineages being between approximately 423.79 and 465.10 million years ago (Mya). Comparatively, using mitochondrial data, it has previously been estimated that spiders and scorpions diverged around 397 ± 23 Mya [67]. Furthermore, we estimated that *T. antipodiana* and *T. clavipes* diverged approximately 16.15 to 19.62 Mya (Figure 2a).

Gene family evolution and GO/KEGG enrichment analyses

Within the *T. antipodiana* genome, we identified 1,186 expanded and 2,480 contracted gene families ($p \leq 0.01$), among which 300 and 143 families have undergone significant expansions and contractions ($p < 0.001$), respectively (Figure 2a). In Figure 2b, we show the 20 families that have undergone the largest expansions.

Among the gene families showing varying degrees of expansion, there are a number that play vital roles in spiders' survival, including those related to immunity, dietary digestion, and detoxification. The expansion of immunity-related gene families, such as putative peptidases,

immunoglobulin I-set domain, and retroviral aspartyl proteases, reflects the powerful innate immune response of spiders [12], whereas certain digestion- and detoxification-related gene families, such as cytochrome P450s, peptidases, and proteases, may reflect mechanisms underlying the wide dietary repertoire of the spider *T. antipodiana*. For example, members of the cytochrome P450 family play important roles in digestion and detoxification by contributing to xenobiotic metabolism and insecticide resistance [68]. Given its large webs and diverse range of prey items, it is essential for *T. antipodiana* to have effective digestion and detoxification systems, and gene ontology (GO) and KEGG pathway enrichment analyses of these expanded genes further confirmed this hypothesis.

Among the GO enrichment results, we noted certain important functions associated with the regulation of hormone levels, oxidoreductase activity, structural constituent of the cuticle, and metabolic and catabolic processes (including hormone, steroid, isoprenoid, and ecdysteroid metabolic processes). The enrichment of these metabolic and catabolic processes is again consistent with the strong detoxification ability of *T. antipodiana* (Figure 3).

Among the KEGG enrichment results (Figure 4), we identified a number of important functions, including cell proliferation and differentiation (such as cancer-related, hedgehog signaling, and notch signaling pathways), biosynthesis, and metabolism (such as linoleic, arachidonic, and drugs) that are consistent with the GO enrichment results. We also detected strong enrichment of drug and xenobiotic metabolism by cytochrome P450.

Analysis of detoxification-related gene families in *T. antipodiana*

Numerous families of genes, including P450s, GSTs, ABCs, and CCEs, play roles in the

detoxification of toxic compounds. When toxin molecules enter the cell, enzymes such as P450s and CCEs render these molecules more reactive and water soluble. These modified toxin molecules are subsequently conjugated by the activities of enzymes such as GSTs, and are eventually transported out of the cell via the action of ABC transporters [69]. For further analysis of the detoxification ability of *T. antipodiana*, we manually annotated the genes of detoxification-related enzymes (P450s, CCEs, GSTs, and ABCs).

From the perspective of xenobiotic metabolism, P450s are the most important superfamily of enzymes in arthropods [68]. In the genome of *T. antipodiana*, we identified 167 CYP genes, comprising four major classes: CYP2 (57 genes), mitochondrial P450 (19), CYP3 (43), and CYP4 (48). Among insects, 46, 81, 85, and 143 P450 genes have been identified in Hymenoptera (*Apis mellifera*), Lepidoptera (*Bombyx mori*), Diptera (*D. melanogaster*), and Coleoptera (*Tribolium castaneum*), respectively [68]. Compared with other arthropods, the number of genes of every class in commonly used model species, such as *D. melanogaster*, show varying degrees of increase (Figure 5). CYP2 enzymes are associated with detoxification and/or bioactivation of certain foreign chemicals [70]. Similar results have been obtained for *T. urticae*, revealing 81 CYP genes with a notable lineage-specific expansion of duplicated intron-less CYP2 clade genes [71]. This expansion of the CYP2 clade in *T. urticae* reflects a strong xenobiotic (acaricide) metabolic capacity and the ability to consume different hosts [71]. With regards to *T. antipodiana*, it is conceivable that the expansion of the CYP2 clade may be associated with its polyphagous habit. In insects, it has been reported that the mitochondrial P450 clade is associated with insecticide resistance [68]; for example, the CYP12A1 gene of the housefly been shown to play a role in the metabolism of xenobiotics, although not insect

ecdysteroids. Furthermore, the CYP3 clade genes have been found to be associated with xenobiotic metabolism and insecticide resistance when induced by phenobarbital, pesticides, or natural products, whereas certain clade CYP4 genes, the least studied among the insect CYP genes, can be induced by xenobiotics as metabolizers, and other are linked to odorant or pheromone metabolism. Moreover, it has been reported that exposure to cadmium toxicity increases expression of cytochrome P450-encoding genes in the wolf spider *Pirata subpiraticus* [72].

The CCE superfamily comprises a functionally diverse group of proteins that hydrolyze carboxylicesters [73]. CCEs not only regulate endogenous compounds (such as hormones, pheromones, and acetylcholine) but also detoxify exogenous compounds derived from dietary or environmental sources. These genes have been categorized into three main phylogenetic classes, namely, hormone/semiochemical processing, dietary/detoxification, and neuro/developmental functions. Within the *T. antipodiana* genome, we identified 48 CCE genes, among which, the overwhelming majority (47) belong to neuro/developmental class, with the single remaining gene belonging to the hormone/semiochemical class (Figure S2). Notably, whereas in the fruit fly *D. melanogaster*, the number of CCEs in the neuro/developmental class is relatively conserved, we detected a clear expansion in the *T. antipodiana* genome (Figure S2), thereby reflecting the difference between spiders and insects [74].

GSTs play roles in cellular detoxification by catalyzing nucleophilic attack of the tripeptide glutathione (GSH) in the electrophilic centers of xenobiotic and endobiotic compounds [75]. Within the *T. antipodiana* genome, we identified 22 GST genes, and phylogenetic analyses of the cytosolic *T. antipodiana* GSTs revealed five different classes of these genes (Figure S3),

namely, Delta/Epsilon (2 genes), Mu (15), Theta (1), Sigma (2), and Zeta (2), among which the Mu class is the largest and shows considerable expansion in *T. antipodiana*. Functionally, the Mu GSTs have been reported to participate in the oxidative stress response associated pesticide resistance in *T. urticae* [76].

The ABCs can act directly on toxicants as primary-active transporters, thereby protecting cells or organisms [69]. The genome of *T. antipodiana* was found to contain 47 ABC genes belonging to seven different classes (Figure S4): ABCA (10 genes), ABCB (12), ABCC (11), ABCD (3), ABCE (1), ABCF (3), and ABCG (7). Among the annotated genomes of arthropod species that have been studies in detail [69], that of *T. urticae* has been found to contain the largest number of ABC genes (103), followed by that of *T. castaneum* (73) and *D. pulex* (65), whereas the genome of *A. mellifera* has only 41 ABC genes.

Analysis of the *T. antipodiana* genome provides evidence in supports of a WGD event

On the basis of our analysis of the *T. antipodiana* genome, we provide three line of evidence in support of the assumption that an ancient WGD probably occurred after the divergence of the common ancestor of spiders and scorpions from other arachnid lineages (mites, ticks, and harvestmen) prior to 430 Mya, which occurred independently of the apparent WGD that is evident in all extant horseshoe crabs [77, 78].

First, synteny analysis revealed the occurrence of certain segmental duplications, the signatures of which are suggestive of a WGD. These signatures were observed in multiple chromosomes, such as chromosomes 2, 3, 9 and 10 (Figure 1). These results are comparable with the findings of a similar analysis of the *P. tepidariorum* genome [79]. The conservation of

synteny within the genome of *T. antipodiana* supports the hypothesis of a WGD event.

Second, we found that the synonymous substitution rate showed a low unimodal distribution, which is indicative of an ancient WGD event. We performed distribution analysis of pairwise synonymous substitution rates (Ks) among the corresponding duplicated genes in the *T. antipodiana* genome, which revealed that the means of the duplicated gene K distributions have a unimodal distribution with a single weak peak between 1.5 and 2.0 (median of 1.75) when plotted together in a single histogram (Figure 6). The peak was weak may because the WGD ancient was too old that the WGD event occurred in the ancestor of scorpions and spiders 450 MYA ago [77]. However, given that the software used for this analysis may lead to the appearance of artificial peaks, owing to the correction for boundary effects [64], we cannot consider these findings as reliable support.

Third, we detected two clusters of Hox genes. Variation in the number of Hox gene clusters is considered to be consistent the occurrence of WGD events during the course of evolution [80]. In the present study, we identified Hox genes of the following classes in the *T. antipodiana* genome: *lab*, *pb*, *Hox3*, *Dfd*, *Scr*, *ftz*, *Antp*, *Ubx*, *abdA*, and *AbdB*. One complete HOX cluster copy was identified on chromosome 12, whereas a further HOX cluster detected on in chromosome was found to be lacking copies of *Hox3*, *ftz*, *ubx*, and *Abd-a* genes (Figure 1). Notably, however, we detected two copies of nearly all the Hox genes in the *T. antipodiana* genome, thereby indicating that entire Hox clusters have been duplicated. The results are consistent with those obtained in a previous study on the house spider *P. tepidariorum* [77].

Conclusion

In this study, we assembled a high-quality chromosome-level genome for the spider *Trichonephila antipodiana*. The assembled genome is 2.29 Gb in size with a contig N50 of 1.172 Mb and a scaffold N50 of 172.89 Mb. Hi- C scaffolding assigned 98.5% of the bases to one of the 13 chromosomes. On the basis of universal single- copy ortholog analysis (BUSCO), the genome assembly was estimated to be 95.2% complete and was predicted to encompass 19,001 protein-coding genes. Analysis of the *T. antipodiana* genome also revealed the expansion and radiation of certain important detoxification-related gene families, including the P450s, CCEs, GSTs, and ABCs, which reflects the unique detoxification activities of this spider. Furthermore, we provide three pieces of evidence in support of the assumption that an ancient WGD event has occurred during the course of spider evolution. The *T. antipodiana* genome reported herein can serve as a valuable reference genome, not only for research on the biological functions of spiders but also for gaining insights on the putative WGD event. Moreover, the chromosomal assembly of *T. antipodiana* will provide useful data for studies on the evolutionary adaptations of spiders and species-specific functions.

Figure Legends

Figure 1. Schematic representation of the genomic characteristics of *Trichonephila. antipodiana*. The inner ring of the circle is based on the findings of inter-chromosome synteny analysis; The outer ring of the circle represents the distribution of genes, GC content, DNA elements, long interspersed elements (LINEs), long terminal repeats (LTRs), short interspersed elements (SINEs), and chromosomes. The location of Hox genes is marked on the outer ring of the chromosome circle.

Figure 2. Phylogenetic and comparative gene family analyses of *Trichonephila antipodiana* and other Arachnida species. The estimated species divergence times (millions of years ago; MYA) are indicated at each branch point. Node values indicate gene families showing expansion (red), contraction (green), and rapid evolution (black in bracket). The bar chart indicates the number of genes classified into six groups (single-copy, multi-copy, species-specific, unassigned, other, and common genes unique to Araneae).

Figure 3. GO annotation of the top 20 expanded gene families.

Figure 4. KEGG annotation of the top 20 expanded gene families.

Figure 5. Expansion of the P450 gene family in *Trichonephila. antipodiana*. The phylogenetic tree shows the orthologous and paralogous relationships of all P450 genes from *T. antipodiana* and *Drosophila melanogaster*. Bootstrap values are indicated on the nodes.

Figure 6. Whole-genome duplications in the *Trichonephila antipodiana* genome

Availability of Supporting Data and Materials

All raw sequencing data and the genome assembly of *T. antipodiana* are available at the National Center for Biotechnology Information (NCBI) under the Bioproject ID PRJNA627506. Other data supporting this work are openly available in the GigaScience repository, GigaDB.

Additional Files

Figure S1. k-mer distribution of the *Trichonephila antipodiana* genome.

Figure S2. Expansion of the CCE gene family in *Trichonephila antipodiana*. The phylogenetic tree shows the orthologous and paralogous relationships of all CCE genes from *T. antipodiana*

and *Drosophila melanogaster*. Bootstrap values are indicated on the nodes.

Figure S3. Expansion of the GST gene family in *Trichonephila antipodiana*. The phylogenetic tree shows the orthologous and paralogous relationships of all GST genes from *T. antipodiana* and *Drosophila melanogaster*. Bootstrap values are indicated on the nodes.

Figure S4. Expansion of the ABC gene family in *Trichonephila antipodiana*. The phylogenetic tree shows the orthologous and paralogous relationships of all ABC genes from *T. antipodiana* and *D. melanogaster*. Bootstrap values are indicated on the nodes.

Abbreviations

WGD: whole genome duplications; BUSCO: Benchmarking Universal Single-Copy Orthologs; Hi-C: High-throughput chromosome conformation capture; PacBio: Pacific Biosciences; P450s: P450 monooxygenases; CCE: carboxyl/cholinesterases; GST: glutathione-*S*-transferases; ABC: ATP-binding cassette transporters; TEs: transposable elements; LINEs: long interspersed elements; LTRs: long terminal repeats; SINEs: short interspersed elements; KEGG: : Kyoto Encyclopedia of Genes and Genomes; GO: Gene Ontology; ECs: expression coherence; Kos: KEGG orthologous groups; NCBI: National Center for Biotechnology Information; KDEs: kernel density estimates; Ks: pairwise synonymous substitution rates; Hox genes: homeotic genes.

Competing Interests

The authors declare that they have no competing interests.

Authors' Contributions

Z.F. performed the major part of data analysis and drafted the manuscript. L.W. T.Y. and P.L. contributed to sample collection. J.J. and F.Z. contributed to data analysis and edits to the manuscript. Z.Z. contributed to research design and final edits to the manuscript. All authors read and approved the final manuscript.

Acknowledgements

This research is funded by the key Natural Science Foundation of Chongqing (No. cstc2019jcyj-zdxmX0006), the Investigation Project of Basic Science and Technology (No. 2018FY100305).

Reference

1 World Spider Catalog (2020). World Spider Catalog. Version 21.5. Natural History Museum Bern, online at http://wsc.nmbe.ch, accessed on {July 26, 2020}. doi: 10.24436/2.

2 Kiseleva AP, Krivoshapkin PV, Krivoshapkina EF. Recent Advances in Development of Functional Spider Silk-Based Hybrid Materials. Front Chem. 2020; **8**: 554.

3 Saez NJ, Herzig V. Versatile spider venom peptides and their medical and agricultural applications. Toxicon. 2019; **158**: 109-26.

4 Chen ZQ, Corlett RT, Jiao XG, et al. Prolonged milk provisioning in a jumping spider. Science. 2018; **362**:1052–5.

5 Joel AC, Weissbach M. Same Principles but Different Purposes: Passive Fluid Handling throughout the Animal Kingdom. Integr Comp Biol. 2019; **59**(6):1673-80.

6 Kuntner M, Coddington JA. Sexual Size Dimorphism: Evolution and Perils of Extreme Phenotypes in Spiders. Annu Rev Entomol. 2020; **65**: 57-80.

7 Harper A, Baudouin Gonzalez L, Schönauer A, et al. Widespread retention of ohnologs in key developmental gene families following whole genome duplication in arachnopulmonates. bioRxiv. 2020. doi: 10.1101/2020.07.10.177725.

8 Yu N, Li J, Liu M, et al. Genome sequencing and neurotoxin diversity of a wandering. bioRxiv. 2019. doi: 10.1101/747147.

9 Sanggaard KW, Bechsgaard JS, Fang X, et al. Spider genomes provide insight into composition and evolution of venom and silk. Nature communications. 2014; **5**: 3765.

10 Babb PL, Lahens NF, Correa-Garhwal SM, et al. The *Nephila clavipes* genome highlights the diversity of spider silk genes and their complex expression. Nature genetics. 2017;

49(6): 895-903.

11 Liu S, Aageaard A, Bechsgaard J, Bilde T. DNA Methylation Patterns in the Social Spider, *Stegodyphus dumicola*. Genes (Basel). 2019; **10**(2).

12 Palmer WJ and Jiggins FM. Comparative Genomics Reveals the Origins and Diversity of Arthropod Immune Systems. Molecular biology and evolution. 2015; **32**(8): 2111-29.

13 Gendreau KL, Haney RA, Schwager EE, et al. House spider genome uncovers evolutionary shifts in the diversity and expression of black widow venom proteins associated with extreme toxicity. BMC genomics. 2017; **18**(1):178.

14 Sanchez Herrero JF, Frias Lopez C, Escuer P, et al. The draft genome sequence of the spider *Dysdera silvatica* (Araneae, Dysderidae): A valuable resource for functional and evolutionary genomic studies in chelicerates. GigaScience. 2019; **8**(8).

15 Sheffer MM, Hoppe A, Krehenwinkel H, et al. Chromosome-level reference genome of the European wasp spider *Argiope bruennichi*: a resource for studies on range expansion and evolutionary adaptation. bioRxiv. 2020. doi: 10.1101/2020.05.21.103564.

16 Harvey MS, Austin AD, Adams M. The systematics and biology of the spider genus Nephila (Araneae:Nephilidae) in the Australasian region. Invertebrate Systematics. 2007; **21**(5).

17 Hawes, TC. A spider that decorates its web perpendicular to the web plane. Tropical zoology. 2019; 32(4): 202-211.

18 Kuntner M, Hamilton CA, Cheng RC, et al. Golden Orbweavers Ignore Biological Rules: Phylogenomic and Comparative Analyses Unravel a Complex Evolution of Sexual Size Dimorphism. Syst Biol. 2019; **68**(4): 555-72.

19 Eggs B, Sander D. Herbivory in Spiders: The Importance of Pollen for OrbWeavers. Plos One. 2013; 8(11): e82637.

20 Bushnell, B. BBtools. 2014. Retrieved from https://sourceforge.net/projects/bbmap/

21 Vurture GW, Sedlazeck FJ, Nattestad M, et al. GenomeScope: fast reference-free genome profling from short reads. Bioinformatics, 2017; **33**(14), 2202–2204.

22 Kolmogorov M, Yuan J, Lin Y, Pevzner PA. Assembly of Long Error-Prone Reads Using Repeat Graphs. bioRxiv 2018.

23 Roach MJ, Schmidt SA, Borneman AR. Purge Haplotigs: allelic contig reassignment for third-gen diploid genome assemblies. BMC Bioinformatics. 2018; **19**(1): 460.

24 Hu J, Fan J, Sun Z, et al. NextPolish: a fast and efficient genome polishing tool for long-read assembly. Bioinformatics. 2020; **36**(7): 2253-5.

25 Li H. Minimap2: pairwise alignment for nucleotide sequences. Bioinformatics. 2018; **34**(18): 3094–3100.

26 Durand NC, Shamim MS, Machol I, et al. Juicer provides a one-click system for analyzing loop-resolution Hi-C experiments. Cell Systems. 2016; 3(1): P95-98.

27 Ghurye J, Pop M, Koren S, et al. Scaffolding of long read assemblies using long range contact information. BMC genomics. 2017; **18**(1): 527.

28 Zhang X, Zhang S, Zhao Q, et al. Assembly of allele-aware, chromosomal-scale autopolyploid genomes based on Hi-C data. Nat Plants. 2019; **5**(8): 833-45.

29 Chen Y, Ye W, Zhang Y, et al. High speed BLASTN: an accelerated MegaBLAST search tool. Nucleic Acids Res. 2015; **43**(16): 7762-8.

30 Camacho C, George C, Vahram A, et al. 2009. BLAST+: architecture and applications.

BMC Bioinformatics. 2009; 10: 421.

31 Waterhouse RM, Seppey M, Simao FA, et al. BUSCO applications from quality assessments to gene prediction and phylogenomics. Mol Biol Evol 2018; **35**(3): 543–8.

32 Flynn PM, Hubley P, Goubert P, et al. RepeatModeler2 for automated genomic discovery of transposable element families. PNAS. 2020; **117** (17): 9451-9457.

33 Bao W, Kojima KK and Kohany O. Repbase Update, a database of repetitive elements in eukaryotic genomes. Mobile DNA. 2015; 6: 11.

34 Smit AFA, Hubley R, Green P. RepeatMasker Open-4.0. 2013-2015. Retrieved from http://www.repeatmasker.org.

35 Nawrocki EP and Eddy SR. Infernal 1.1: 100-fold faster RNA homology searches. Bioinformatics. 2013; 29(22): 2933-5.

36 Chan PP and Lowe TM. tRNAscan-SE: Searching for tRNA Genes in Genomic Sequences. Methods in Molecular Biology. 2019; 1962: 1–14.

37 Holt C and Yandell M. MAKER2: an annotation pipeline and genome-database management tool for second-generation genome projects. BMC Bioinformatics. 2011; 12: 491.

38 Stanke M, Steinkamp R, Waack S, et al. AUGUSTUS: a web server for gene finding in eukaryotes. Nucleic Acids Research. 2004; 32: W309–W312.

39 Brůna T, Lomsadze A and Borodovsky M. GeneMark-EP+: eukaryotic gene prediction with self-training in the space of genes and proteins. NAR Genom Bioinform. 2020; **2**(2): lqaa026.

40 Hoff KJ, Lange S, Lomsadze A, et al. BRAKER1: Unsupervised RNA-Seq-Based

Genome Annotation with GeneMark-ET and AUGUSTUS. Bioinformatics. 2016; 32: 767–769.

41 Kim D, Landmead B, Salzberg SL. HISAT: a fast spliced aligner with low memory requirements. Nat Methods. 2015; **12**(4): 357-U121.

42 Kovaka S, Zimin AV, Pertea GM, et al. Transcriptome assembly from long-read RNA-seq alignments with StringTie2, Genome Biology. 2019. 16; **20**(1):278.

43 Buchfink B, Xie C, and Huson DH. Fast and sensitive protein alignment using DIAMOND. Nature Methods. 2015; **12**(1):59-60.

44 Finn RD, Attwood TK., Babbitt PC, et al. InterPro in 2017-beyond protein family and domain annotations. Nucleic Acids Research. 2017; 45: D190–D199.

45 El-Gebali S, Mistry J, Bateman A, et al. The Pfam protein families database in 2019. Nucleic Acids Res. 2019; 47: D427–D432.

46 Mi HY and Thomas P. PANTHER Pathway: an ontology-based pathway database coupled with data analysis tools. Methods Mol Biol. 2009; 563: 123–140.

47 Lewis TE, Sillitoe I, Dawson N, et al. Gene3D: extensive prediction of globular domains in proteins. Nucleic Acids Research. 2018; 46: D435–D439.

48 Wilson D, Pethica R, Zhou Y, et al. SUPERFAMILY—sophisticated comparative genomics, data mining, visualization and phylogeny. Nucleic Acids Research. 2009; 37: D380–D386.

49 Marchler-Bauer A, Bo Y, Han L, et al. CDD/SPARCLE: functional classification of proteins via subfamily domain architectures. Nucleic Acids Research. 2017; 45: D200–D203.

50 Huerta-Cepas J, Forslund K, Coelho PL, et al. Fast genome-wide functional annotation through orthology assignment by eggNOG-mapper. Mol Biol Evol. 2017; 34(8): 2115–2122.

51 Huerta-Cepas J, Szklarczyk D, Heller D, et al. eggNOG 5.0: a hierarchical, functionally and phylogenetically annotated orthology resource based on 5090 organisms and 2502 viruses. Nucleic Acids Res. 2019; 47: D309–D314.

52 Emms DM, Kelly S. OrthoFinder: phylogenetic orthology inference for comparative genomics. Genome Biol. 2019; 20(1): 238.

53 Katoh K, and Standley DM. MAFFT multiple sequence alignment software version 7: Improvements in performance and usability. Molecular Biology and Evolution. 2013; 30: 772–780.

54 Capella Gutierrez S, Silla Martinez JM, Gabaldon T. TrimAl: a tool for automated alignment trimming in large-scale phylogenetic analyses. Bioinformatics. 2009; **25**(15): 1972-3.

55 Kück P, and Longo GC. FASconCAT-G: extensive functions for multiple sequence alignment preparations concerning phylogenetic studies. Frontiers in Zoology. 2014; **11**(1):81.

56 Minh BQ, Schmidt HA, Chernomor O. IQ-TREE 2: New Models and Efficient Methods for Phylogenetic Inference in the Genomic Era. Mol Biol Evol. 2020; 37(5): 1530-1534.

57 Yang Z. PAML 4: phylogenetic analysis by maximum likelihood. Mol Biol Evol. 2007; **24**(8): 1586– 91.

58 Han MV, Thomas GW, Lugo-Martinez J, Hahn MW. 2013. Estimating gene gain and loss

rates in the presence of error in genome assembly and annotation using CAFE 3. Mol Biol Evol. 30(8):1987–1997.

59 Chen CJ, Chen H, Zhang Y, et al. TBtools: An Integrative Toolkit Developed for Interactive Analyses of Big Biological Data. Molecular Plant. 2020; **13**(8): 1194-1202.

60 Steinegger M, Soding J. MMseqs2 enables sensitive protein sequence searching for the analysis of massive data sets. Nature biotechnology. 2017; **35**(11): 1026-8.

61 Mulder N, Apweiler R. InterPro and InterProScan: tools for protein sequence classification and comparison. Methods Mol Biol. 2007; 396: 59-70.

62 Werck-Reichhart, D. & Feyereisen, R. Cytochromes P450: a success story. Genome Biol. 2000; 1: 1-9.

63 Wang YP, Tang HB, Jeremy DD, et al. MCScanX: a toolkit for detection and evolutionary analysis of gene synteny and collinearity. Nucleic Acids Res. 2012; **40**(7): e49.

64 Zwaenepoel A, Van de Peer Y, Hancock J. wgd—simple command line tools for the analysis of ancient whole-genome duplications. Bioinformatics. 2019; **35**(12): 2153-5.

65 Pace RM, Grbic M, and Nagy LM. Composition and genomic organization of arthropod Hox clusters. Evodevo. 2016, 7:11.

66 Zhong YF, Holland PW. HomeoDB2: functional expansion of a comparative homeobox gene database for evolutionary developmental biology. Evol Dev. 2011; **13**(6): 567-8.

67 Jeyaprakash A, Hoy MA. First divergence time estimate of spiders, scorpions, mites and ticks (subphylum: Chelicerata) inferred from mitochondrial phylogeny. Experimental & Applied Acarology. 2009, **47**(1):1.

68 Feyereisen R. Evolution of insect P450. Biochem Soc Trans. 2006; **34**(6): 1252-5.

69 Dermauwa W and Leeuwen TV. The ABC gene family in arthropods: Comparative genomics and role in insecticide transport and resistance. Insect Biochemistry and Molecular Biology. 2014; 45: 89-110.

70 Kubota A, Stegeman JJ, Goldstone JV, et al. Goldstone Cytochrome P450 CYP2 genes in the common cormorant: evolutionary relationships with 130 diapsid CYP2 clan sequences and chemical effects on their expression. Comp Biochem Physiol C Toxicol Pharmacol. 2011; **153**(3): 280–289.

71 Leeuwen TV, Dermauw W. The Molecular Evolution of Xenobiotic Metabolism and Resistance in Chelicerate Mites. Annu Rev Entomol 2016; 61: 475-98.

72 Lv B, Wang J, Zhuo JZ, Yang HL, Yang SF, Wang Z, Song QS. Transcriptome sequencing reveals the effects of cadmium toxicity on the cold tolerance of the wolf spider Pirata subpiraticus. Chemosphere. 2020; 254.

73 Tsubota T, Shiotsuki T. Genomic and phylogenetic analysis of insect carboxyl/cholinesterase genes. Journal of Pesticide Science. 2010; **35**(3): 310-4.

74 Yan LZ, Yang PC, Jiang F, et al. The expansion of this genes is reflected in the enormous diversity in habitats and food sources utilized, which provide an insight into its evolution of specific and amazing adaptations. BMC Genomics. 2012; **13**:609.

75 Fang SM. Insect glutathione S-transferase: a review of comparative genomic studies and response to xenobiotics. B Insectol. 2012;65(2):265-71.

76 Pavlidi N, Tseliou V, Riga M, et al. Functional characterization of glutathione S-transferases associated with insecticide resistance in *Tetranychus urticae*. Pestic Biochem Physiol. 2015; 121:53–60.

77 Schwager EE, Sharma PP, Clarke T, et al. The house spider genome reveals an ancient whole-genome duplication during arachnid evolution. BMC biology. 2017; **15**(1): 62.

78 Pace RM, Grbic M, Nagy LM. Composition and genomic organization of arthropod Hox clusters. Evodevo. 2016; 7 :11.

79 Aury JM, Jaillon O, Duret L, et al. Global trends of whole-genome duplications revealed by the ciliate *Paramecium tetraurelia*. Nature. 2006; **444**(7116): 171-8.

80 Hrycaj SM, Wellik DM. Hox genes and evolution. F1000Res. 2016; **5**.

Table 1. Comparison of the quality of the *Trichonephila antipodiana* genome with that of other published spider genomes

Species	Genome size (Gb)	Scaffold N50 (kbp)	Contig N50 (kbp)	Accession number
Stegodyphus dumicola	2.55	254.13	254.13	GCA_010614865.1
Anelosimus studiosus	2.03	4.79	1.13	GCA_008297655.1
Pardosa pseudoannulata	4.21	711.40	23.23	GCA_008065355.1
Latrodectus Hesperus	1.23	39.47	15.96	GCA_000697925.2
Dysdera silvatica	1.36	38.02	25.71	GCA_006491805.1
Loxosceles reclusa	3.26	63.24	1.83	GCA_001188405.1
Trichonephila clavipes	2.44	62.96	7.99	GCA_002102615.1
Parasteatoda tepidariorum	1.45	4,055.36	10.15	GCA_000365465.3
Stegodyphus mimosarum	2.74	480.64	40.15	GCA_000611955.2
Araneus ventricosus	3.65	59.62	-	BGPR01000001-BGPR01300721 (DDBJ)
Argiope bruennichi	1.67	124,236.00	288.40	unpublicized
Trichonephila antipodiana	2.29	172,892.00	1,138.00	-

Table 2. Statistics of the DNA sequence data used for genome assembly

Pair-end libraries	Clean data (Gb)	Sequencing coverage (×)	Insert sizes
Illumina reads	305.96	133	300 bp
PacBio reads	235.79	103	20 Kb
Hi-C	215.05	94	300 bp
RNA	10.27	-	300 bp
Total	767.07	-	-

Table 3. Summary of each step in construction of the *Trichonephila antipodiana* genome assembly

Assembly	Total length (Gb)	No. scaffolds (chromosome)	N50 length (Mb)	Longest scaffold (MB)	GC (%)	BUSCO (n = 1066) (%)			
						C	D	F	M

Flye	2.38	16,680	1.21	11.071	31.8	95.2	5.2	0.9	3.9
Purge Dups	2.31	10,670	1.26	11.071	31.8	95.3	4.0	0.8	3.9
Pilon	2.31	10,670	1.26	11.082	31.7	95.3	4.3	0.7	4.0
Hi-C	2.29	377(13)	137.66	230.27	31.7	94.8	4.1	1.0	4.2
Final genome assembly	2.29	377(13)	137.66	230.17	31.7	94.8	4.1	1.0	4.2
Transcript assembly	69.29 Mb	30,586	3.43 Kb	43.99 Kb	34.3	97.2	33.4	1.1	1.7

Table 4. Statistics of the repetitive sequences identified in *Trichonephila antipodiana*

Type	Number	Length (bp)	% of genome
**SINEs:**	**106,507**	**25,417,898**	**1.11**
tRNA-Deu	44,262	10,710,146	0.47
MIR	28,198	6,417,754	0.28
tRNA-Core	19,575	5,140,899	0.22
tRNA	3,964	507,398	0.02
**LINEs**	**197,390**	**83,281,087**	**3.63**
Penelope	49,982	30,623,444	1.33
I	56,156	19,510,142	0.85
I-Jockey	27,196	13,846,368	0.60
R1	14,033	5,652,471	0.25
**LTR elements**	**101,690**	**79,698,444**	**3.47**
Gypsy	53,122	53,035,139	2.31
Pao	26,368	19,084,383	0.83
Copia	15,295	6,965,923	0.30
ERV1	4,052	178,070	0.01
**DNA elements**	**1,393,742**	**518,114,026**	**22.58**
TcMar-Tc1	332,152	164,809,170	7.18
hAT-Charlie	399,282	142,099,848	6.19
TcMar-Mariner	89,418	39,370,728	1.72
Kolobok-Hydra	37,030	30,581,663	1.33
**Unclassified**	**1,961,792**	**508,599,211**	**22.17**
**Total interspersed repeats**		**1,215,110,666**	**52.96**
**Small RNA**	**72,066**	**16,577,914**	**0.72**
**Satellites**	**7,513**	**2,910,802**	**0.13**
**Simple repeats**	**450,644**	**24,805,223**	**1.08**
**Low complexity**	**84,430**	**4,336,839**	**0.19**

Figure1

Click here to access/download;Figure;Figure1.pdf

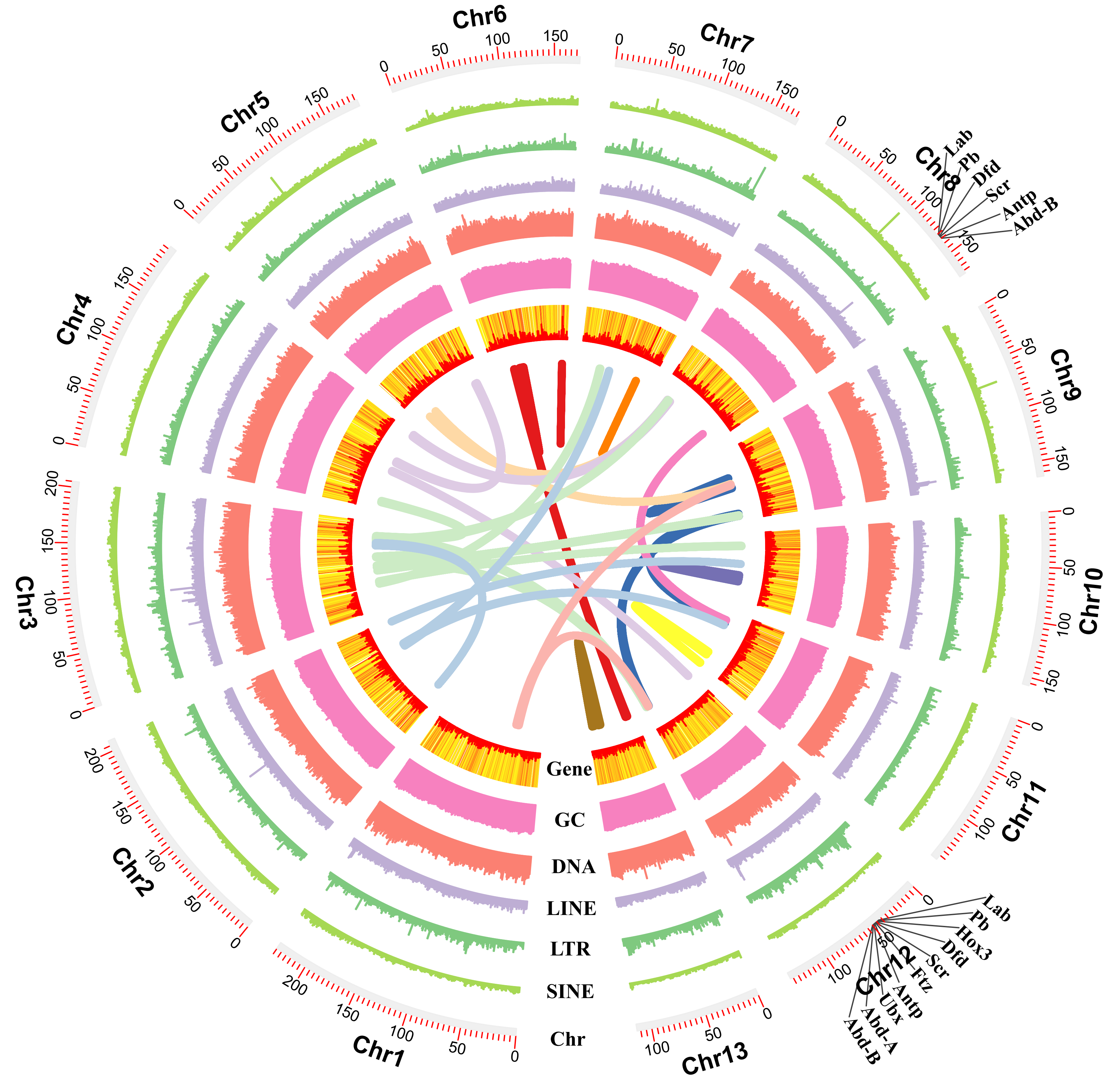

Figure2

Click here to access/download;Figure;Figure2.pdf

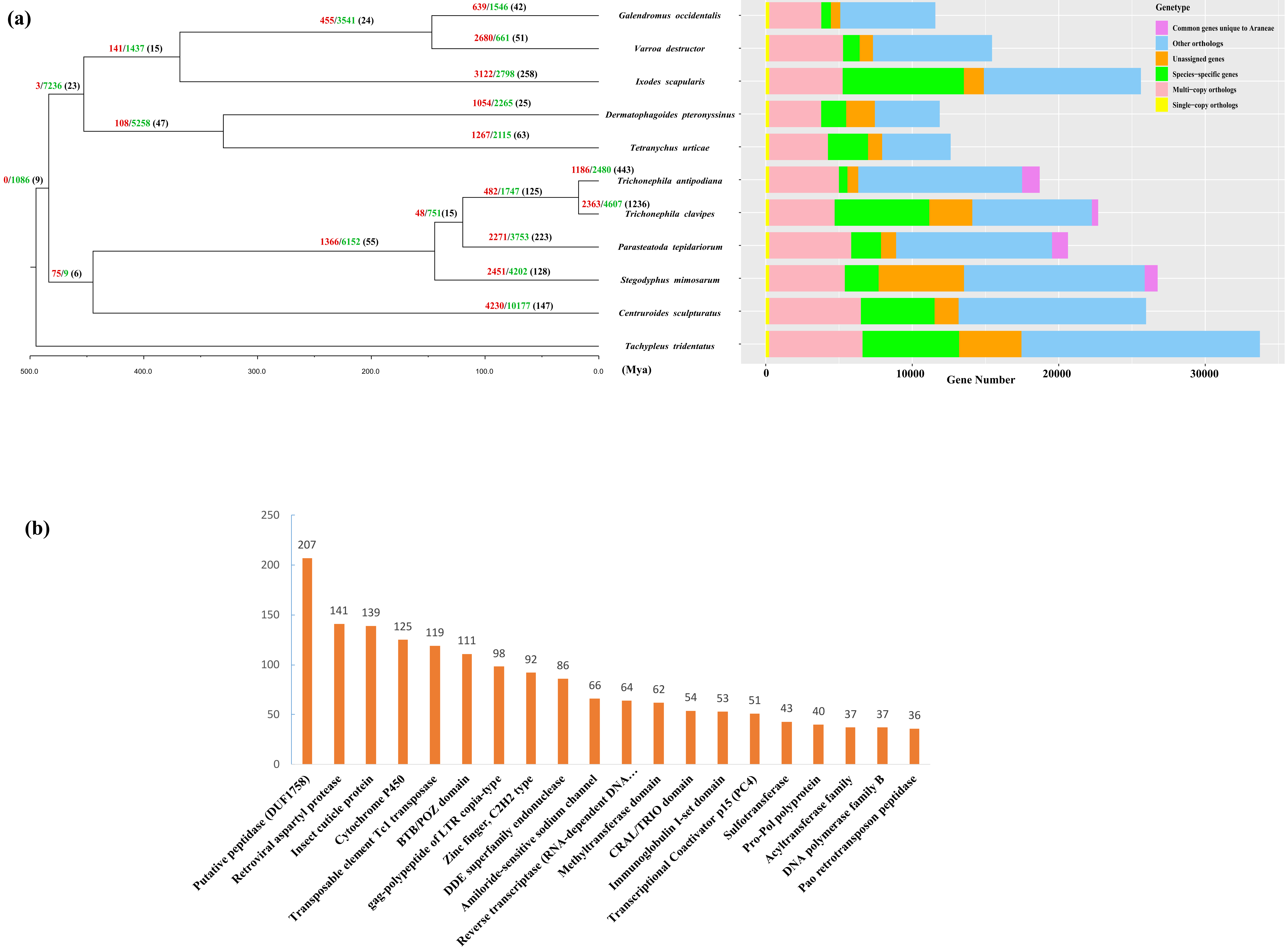

Figure3

Click here to access/download;Figure;Figure3.pdf

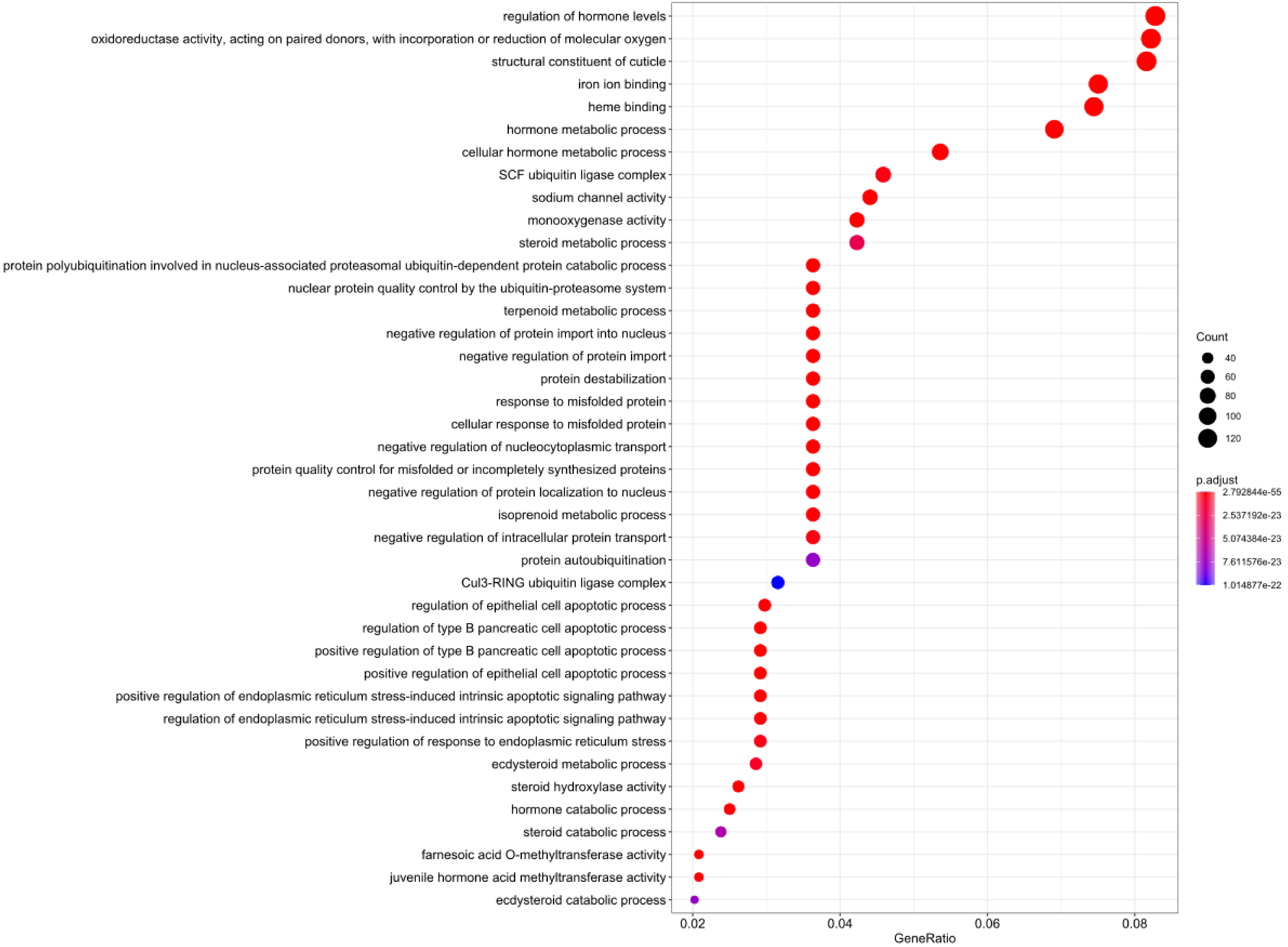

Click here to access/download;Figure;Figure4.pdf

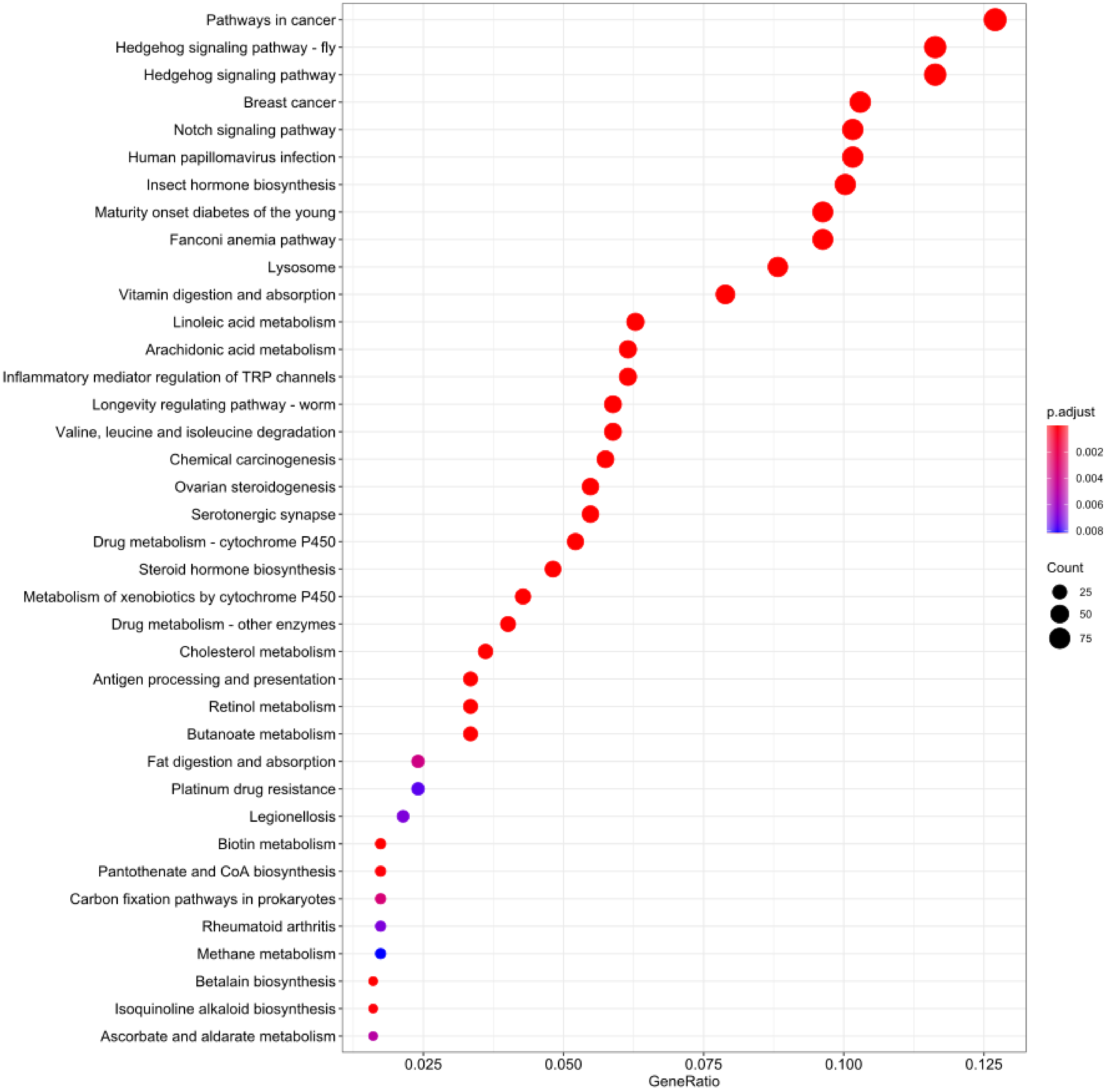

Click here to access/download;Figure;Figure5.pdf

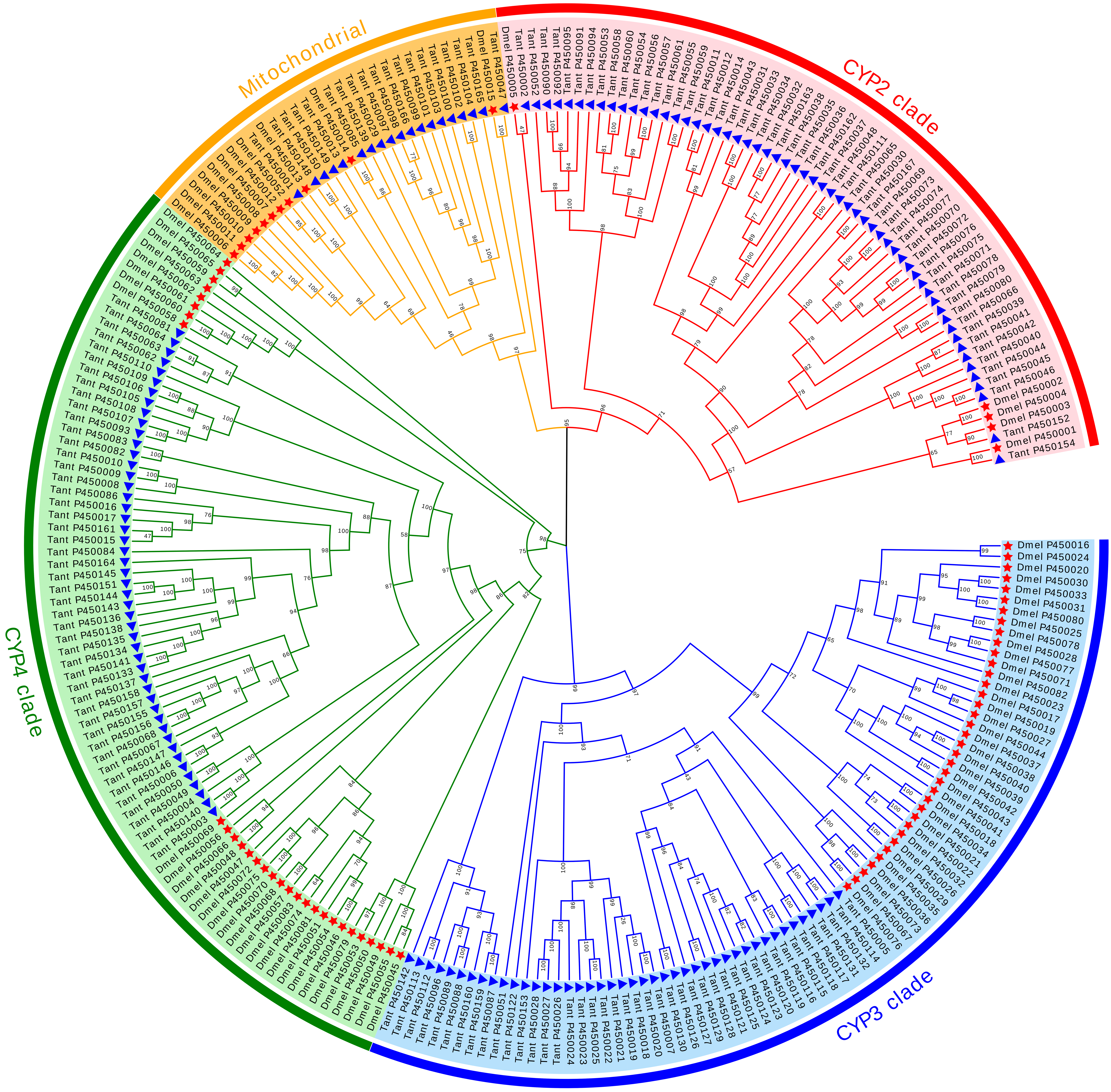

Figure6

Click here to access/download;Figure;Figure6.pdf

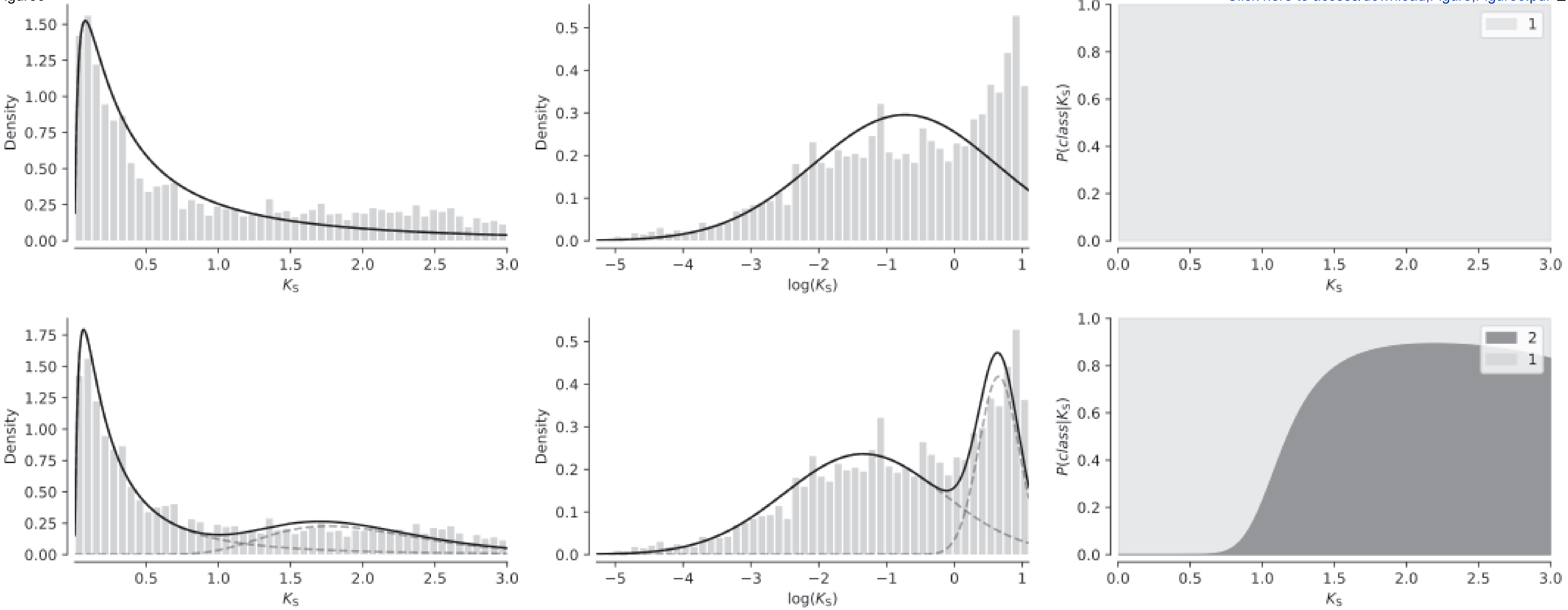

FigureS1

Click here to access/download;Figure;FigureS1.pdf

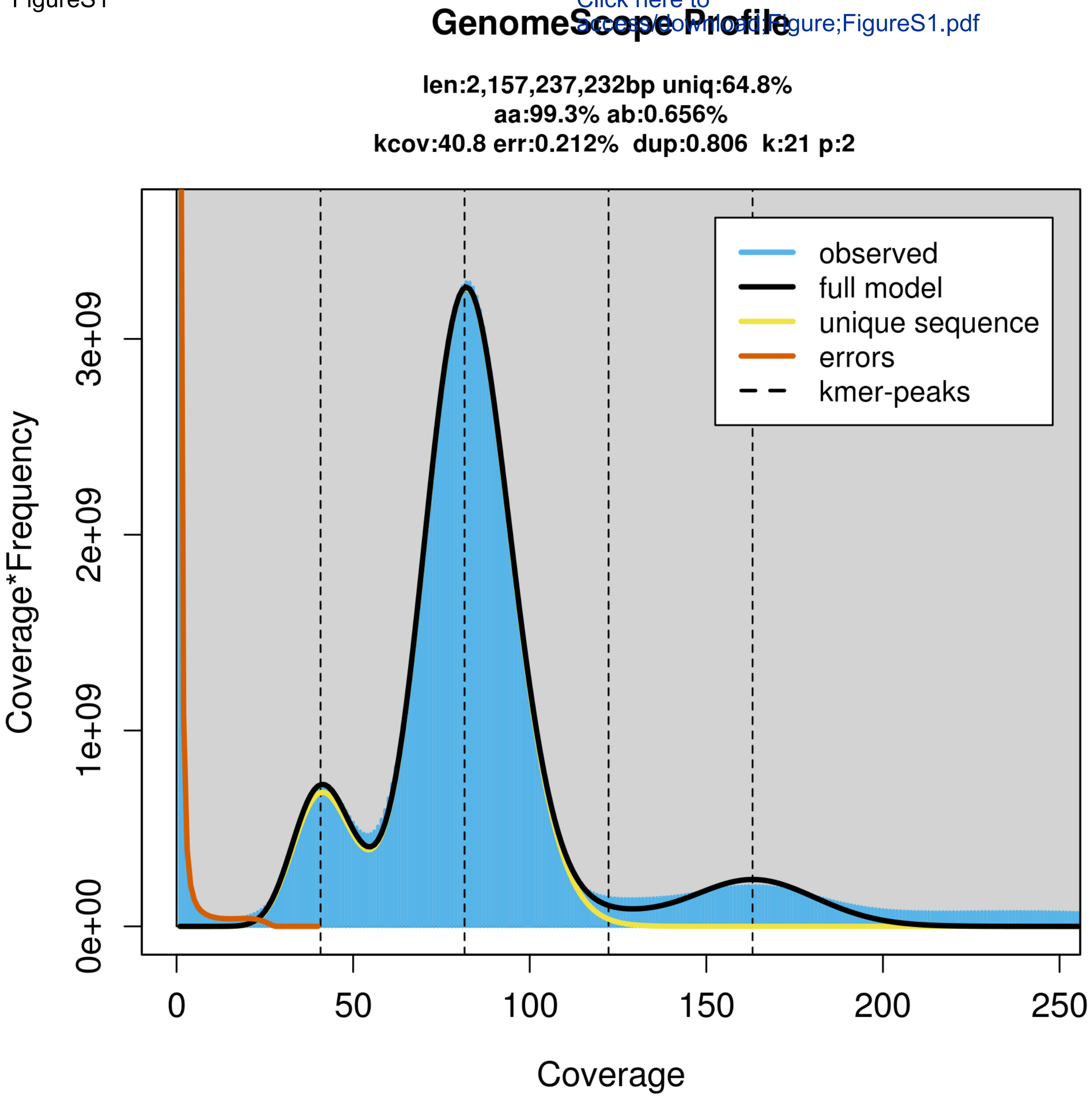

FigureS2
Click here to access/download;Figure;FigureS2.pdf

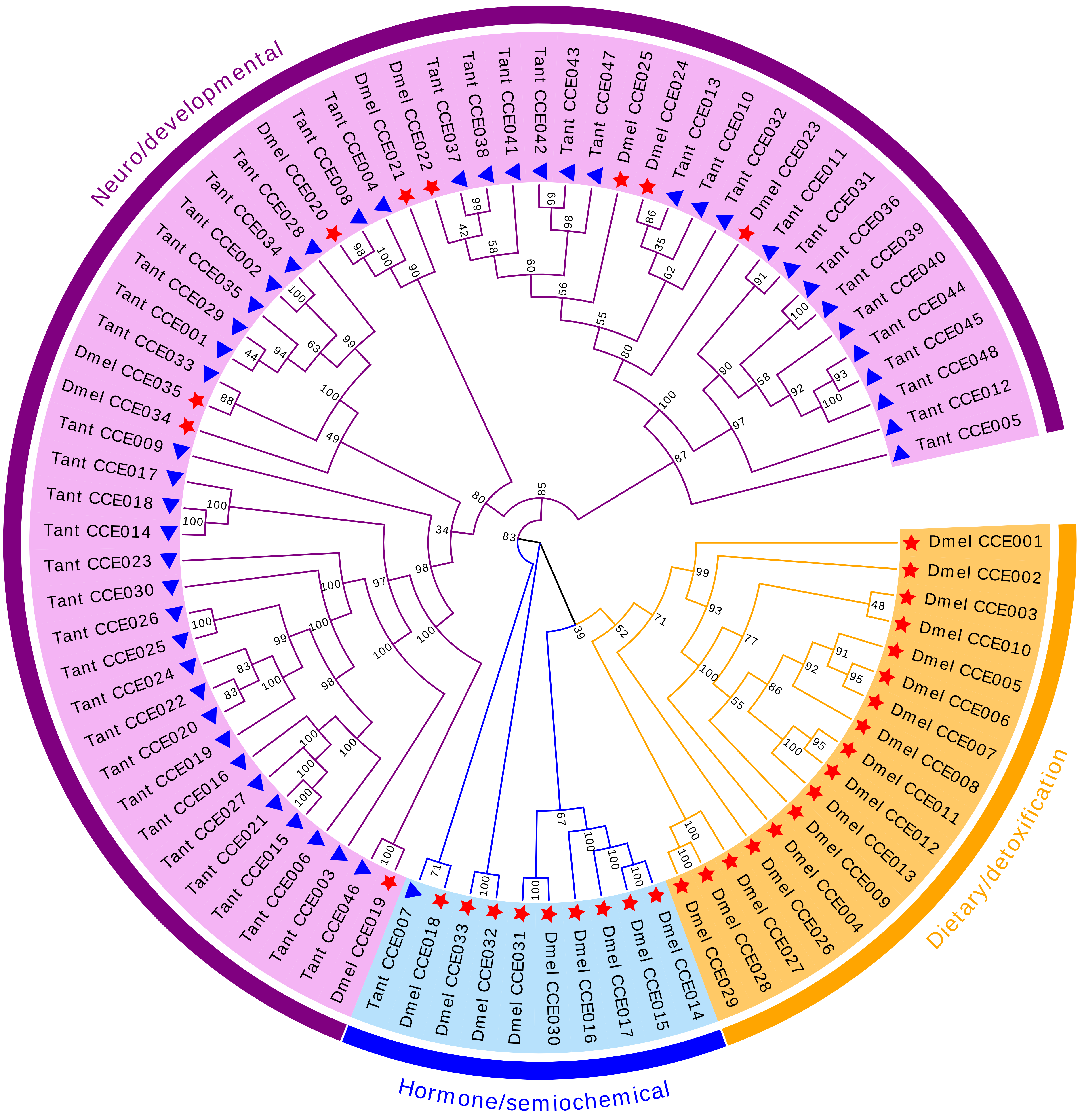

Click here to access/download;Figure;FigureS3.pdf

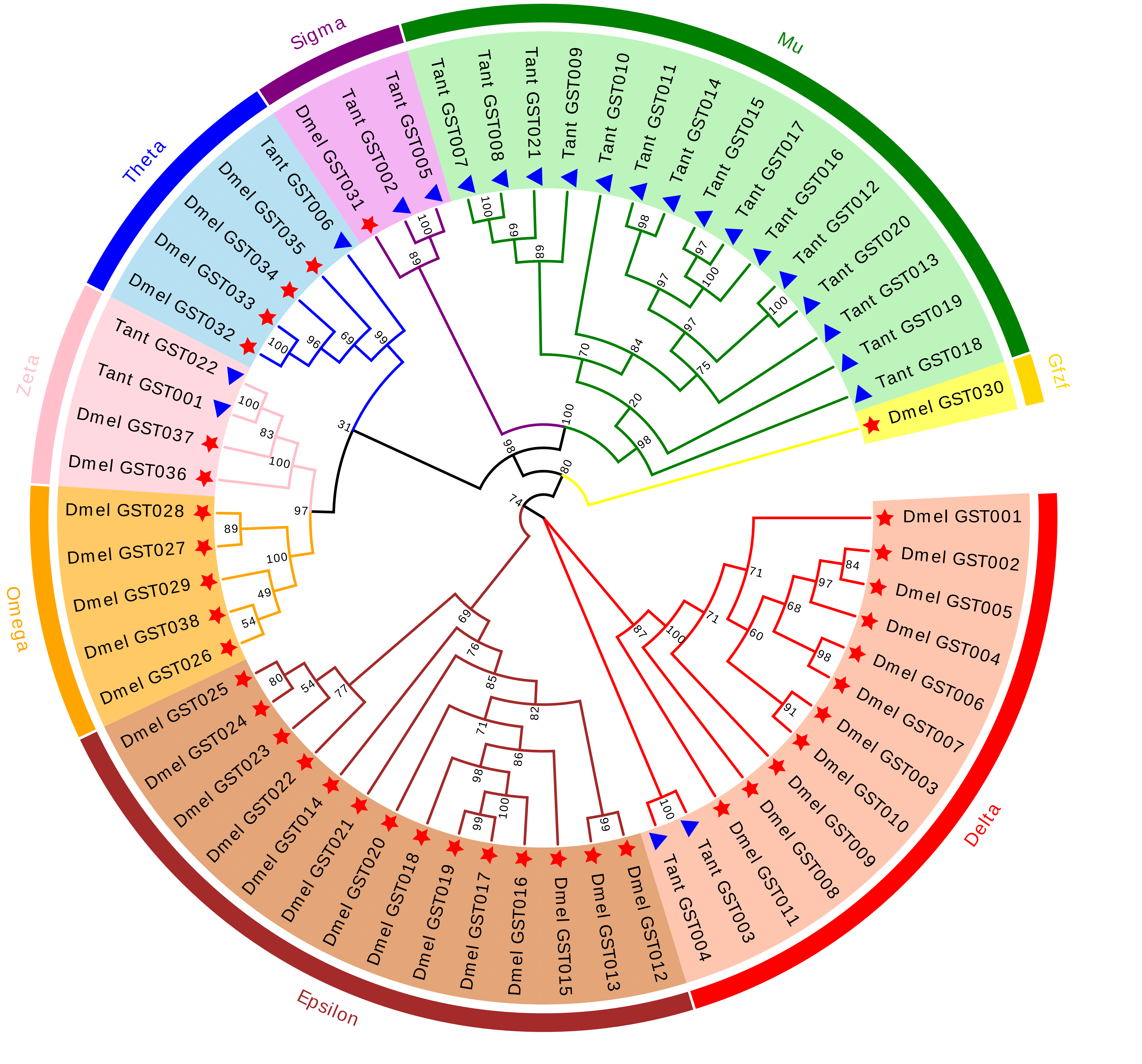

Click here to access/download;Figure;Figures4.pdf

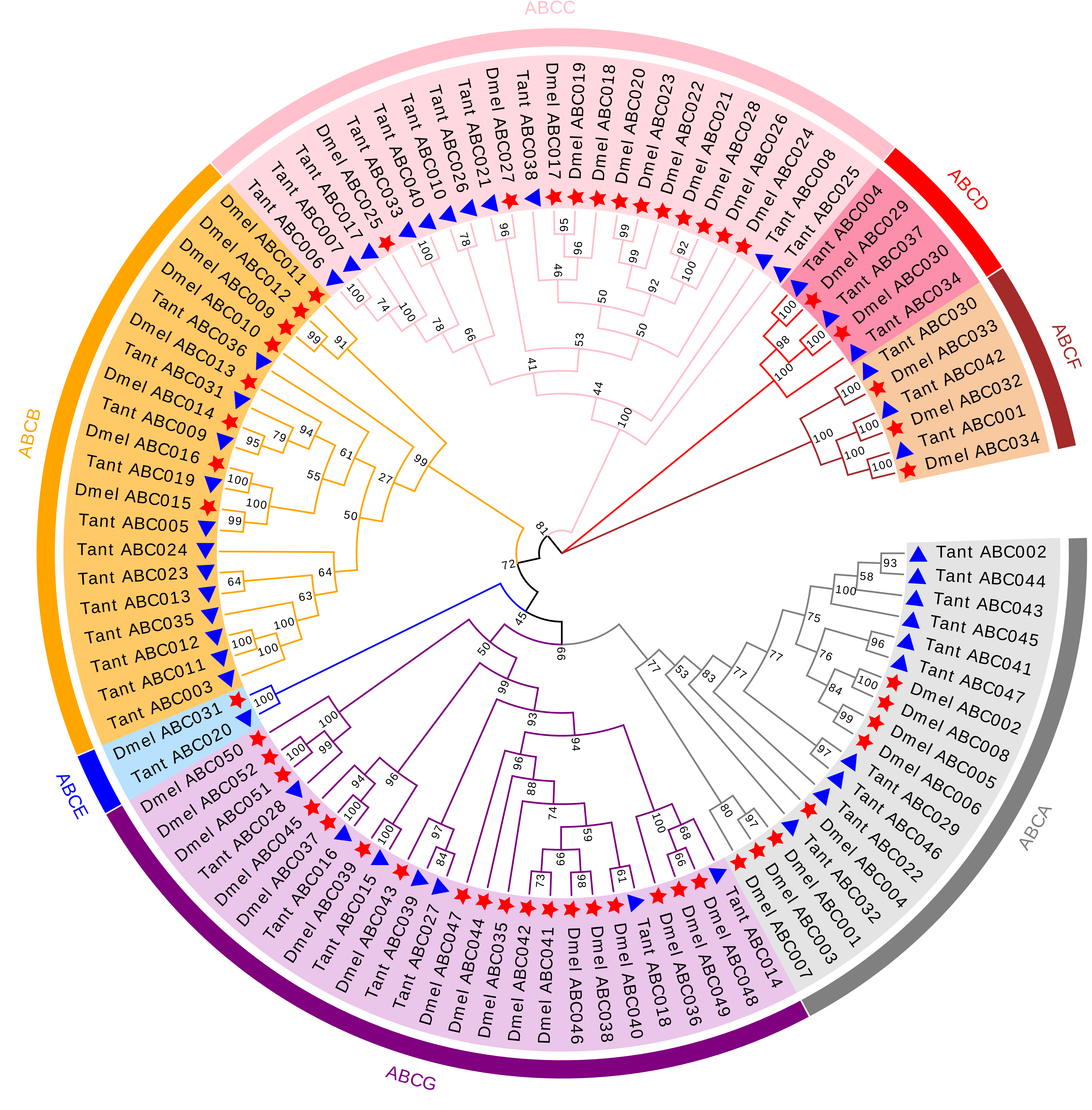

GST proteins of *Trichonephila antipodiana*

Click here to access/download
Supplementary Material
GST.Tan.fa

ABC proteins of Trichonephila antipodiana

Click here to access/download
Supplementary Material
ABC.Tant.fa

CCE proteins of Trichonephila antipodiana

Click here to access/download

Supplementary Material

CCE.Tan.fa

P450 proteins of Trichonephila antipodiana

Click here to access/download
Supplementary Material
P450.Tant.fa

Cover letter Click here to access/download;Personal Cover;cover letter.doc

Dear Editors,

We would like to submit the enclosed manuscript, entitled "**A chromosome- level genome of the spider *Trichonephila antipodiana* reveals the genetic basis of its polyphagy and evidence of an ancient whole-genome duplication event**" for your consideration of publication as an original research paper in "**GigaScience**".

Spiders (Araneae) have a worldwide distribution, have conquered virtually all ecological environments, and exhibit considerable species richness. A total of 48,683 spider species have been described to date, classified into 4,175 genera and 128 families. To date, the genomes of 11 species of spider have been published or are available in the NCBI database, which offer unprecedented insights into the unique biology of these arthropods. However, spider genomes tend to be difficult to sequence, assemble, and annotate owing to their large size and high heterozygosity and repeat content. To date, the genomes of only two species (*Dysdera silvatica* and *Argiope bruennichi*) have been sequenced based on long sequencing reads (PaciBio or Nanopore), only one of which was assembled to the chromosome level.

In this study, we sequenced the whole genome of the batik golden web spider, *Trichonephila antipodiana*. The spider *T. antipodiana* (Araneidae), commonly known as the batik golden web spider, preys on arthropods with body sizes ranging from approximately 2 mm in length to insects larger than itself (over 20–50 mm), indicating its polyphagy and strong dietary detoxification abilities. We present a chromosome- level *T. antipodiana* genome constructed based on PacBio and Hi-C sequencing. The assembled genome is 2.29 Gb in size with a scaffold N50 of 172.89 Mb, and we predicted 19,001 protein-coding genes. Analysis of the *T. antipodiana* genome also revealed the expansion and radiation of certain important detoxification-related gene families, including the P450s, CCEs, GSTs, and ABCs, which reflects the unique detoxification activities of this spider. Furthermore, we provide three pieces of evidence in support of the assumption that an ancient WGD event has occurred during the course of spider evolution.

We believe these findings will be of interest to a broad audience of evolutionary and developmental biologists, as well as to researchers working to understand the molecular basis

of the evolution and development in the *T. antipodiana*. In addition, these genomic resources provided an evidence of the ancient WGD event in spider.

As a premier international journal devoted to the rapid dissemination of significant biological findings, **GigaScience** represents the ideal platform for sharing these results with the international research community.

We thank you for your consideration of our manuscript and look forward to hearing from you at your earliest convenience.

Yours sincerely,

Zhisheng Zhang, Ph.D

School of Life Sciences, Southwest University, Chongqing 400700, China.

Email: zhangzs327@qq.com
