# Supplementary material for: A chromosome‐level genome of the spider Trichonephila antipodiana reveals the genetic basis of its polyphagy and evidence of an ancient whole-genome duplication event
Source: Gigascience. 2021 Mar 19;10(3):giab016. doi: 10.1093/gigascience/giab016 (PMC7976613; doi:10.1093/gigascience/giab016)
Supplement: giab016_GIGA-D-20-00316_Revision_1 [file giab016_giga-d-20-00316_revision_1.pdf]

GigaScience

A chromosome-level genome of the spider Trichonephila antipodiana reveals the genetic basis of its polyphagy and evidence of an ancient whole-genome duplication event

--Manuscript Draft--

Manuscript Number:	GIGA-D-20-00316R1
Full Title:	A chromosome-level genome of the spider Trichonephila antipodiana reveals the genetic basis of its polyphagy and evidence of an ancient whole-genome duplication event
Article Type:	Data Note
Funding Information:	the key Natural Science Foundation of Chongqing (cstc2019jcyj-zdxmX0006) — Mr Zhisheng Zhang<br>the Investigation Project of Basic Science and Technology (2018FY100305) — Not applicable
Abstract:	Background : The spider Trichonephila antipodiana (Araneidae), commonly known as the batik golden web spider, preys on arthropods with body sizes ranging from approximately 2 mm in length to insects larger than itself (over 20 – 50 mm), indicating its polyphagy and strong dietary detoxification abilities. Although it has been reported that an ancient whole-genome duplication event occurred in spiders, lack of a high-quality genome has limited characterization of this event.<br>Results: We present a chromosome-level T. antipodiana genome constructed based on PacBio and Hi-C sequencing. The assembled genome is 2.29 Gb in size with a scaffold N50 of 172.89 Mb. Hi-C scaffolding assigned 98.5% of the bases to 13 pseudo-chromosomes, and BUSCO completeness analysis revealed that the assembly included 94.8% of the complete arthropod universal single-copy orthologs (n=1,066). Repetitive elements account for 59.21% of the genome. We predicted 19,001 protein-coding genes, of which 96.78% were supported by transcriptome-based evidence and 96.32% matched protein records in the UniProt database. The genome also shows substantial expansions in several detoxification -associated gene families, including cytochrome P450 monooxygenases, carboxyl/cholinesterases, glutathione- S -transferases, and ATP-binding cassette transporters, reflecting the possible genomic basis of polyphagy. Further analysis of the T. antipodiana genome architecture reveals an ancient whole-genome duplication event, based on two lines of evidence: (1) large-scale duplications from inter-chromosome synteny analysis; (2) duplicated clusters of Hox genes.<br>Conclusions: The high-quality T. antipodiana genome represents a valuable resource for spider research and provides insights into this species' adaptation to the environment.
Corresponding Author:	Zhisheng Zhang, Ph.D<br>Southwest University<br>Chongqing, CHINA
Corresponding Author Secondary Information:	
Corresponding Author's Institution:	Southwest University
Corresponding Author's Secondary Institution:	
First Author:	Zheng Fan
First Author Secondary Information:	
Order of Authors:	Zheng Fan
	Tao Yuan
	Piao Liu

Powered by Editorial Manager® and ProduXion Manager® from Aries Systems Corporation

	Luyu Wang
	Jianfeng Jin
	Feng Zhang
	Zhisheng Zhang, Ph.D
**Order of Authors Secondary Information:**	
**Response to Reviewers:**	Response to editor:<br>In addition, please also include a picture of the sequenced spider species as a figure in the paper, if available.<br><br>Response:<br>Thanks for your suggestion. We added it, shown in Figure1.<br><br><br>Response to reviewer#1:<br>1. Molecular dating: The molecular dating should be redone with a more recent calibration because the PaleoDB database is outdated for Chelicerata. The split between scorpions and spiders should be assigned a minimum age of 435-439 Mya, based on the recently described fossils Eramoscorpius brucensis (Waddington et al. 2015) and Parioscorpio venator (Wendruff et al. 2020). The current split between these two groups in the dated tree has an implausibly young floor (423.79 Mya). This change will have little effect on the overall manuscript.<br><br>Response:<br>Thanks for your suggestions. We redone the divergence analysis with split between scorpions and spiders assigned a minimum age of 435-439 Mya and changed my manuscript.<br><br>2. Lines 198 and 361: What is the source of these 236 genes? How were they inferred to be single copy and why were these specific loci chosen?<br><br>Response:<br>The source of these 236 genes were obtained from the results of orthologous gene clusters analysis by the soft of OrthoFinder. Using OrthoFinder, we obtained a total of 203,348 genes among the 11 species, which were clustered into 20,785 orthogroups. The single-copy orthologs was identified when every species only included one gene. These specific loci were chosen because the single copy genes have one physical location in the genome and can have orthologs in different species.<br><br>3. The authors should avoid the use of Ks distributions to test this whole genome duplication. It is well known that this method is highly sensitive and fails for ancient (pre-Cretaceous) WGD events due to saturation. Given that this duplication must be older than 439 Myr, there is no chance that Ks distance distributions are going to be dispositive of a WGD event.<br><br>Response:<br>Accept. We deleted the distribution analysis of pairwise synonymous substitution rates (Ks).<br><br>4. In two places in the manuscript, the hypothesis of the arachnopulmonate WGD is referred to as an "assumption". This term is inappropriate in this context, given the large swath of evidence supporting this hypothesis. I would recommend the authors to refer to this idea as a "hypothesis" or an "inference", as well as include references to the following works:<br>a. Leite DJ, Ninova M, Hilbrant M, et al. 2016 Pervasive microRNA Duplication in Chelicerates: Insights from the Embryonic microRNA Repertoire of the Spider Parasteatoda tepidariorum. Genome Biol. Evol 8:2133-2144. (doi: 10.1093/gbe/evw143)<br>b. Leite DJ, et al. (2018) Homeobox gene duplication and divergence in arachnids. Mol. Biol. Evol. 35:2240-2253. (doi: 10.1093/molbev/msy125)<br>c. Nolan ED, Santibáñez-López CE, Sharma PP. 2020 Developmental gene expression as a phylogenetic data class: support for the monophyly of

Arachnopulmonata. Dev. Genes Evol. 230:137-153. (doi: 10.1007/s00427-019-00644-6)

Response:
Accept. We changed and added the references (90, 91, 92).

5. It is not explained in the methods how synteny analysis was performed. Please explain the pipeline or the software used to make these inferences.

Response:
The synteny analysis pipeline was shown in the methods of WGD analyses (line 278-283 and 292-293).

Minor comments:

1. Lines 40-41: "Spiders (Araneae) have a worldwide distribution, have conquered virtually all ecological environments". Please change this to read "virtually all terrestrial environments".

Response:
Accept. We changed it.

2. Line 62: "PacBio"

Response:
Accept. We changed it.

3. Line 254: "It has been reported that an ancient WGD event occurred in the spider lineage". Please replace this with "It has been reported that an ancient WGD event occurred in the common ancestor of spiders and scorpions".

Response:
Accept. We changed it.

4. Line 269: "In metazoans, the 10 highly conserved Hox genes play important roles…" The number of Hox genes is highly variable across Metazoa. It is inferred that the common ancestor of Panarthropoda bore 10 Hox genes, though this number varies across arthropod lineages as well. I suggest to change this to: "In arthropods, 10 highly conserved Hox genes that are inferred to occur in the common ancestor of Panarthropoda play important roles…"

Response:
Accept. We changed it.

5. Lines 459-460: On the WGD events in Xiphosura, please be sure to cite two recent papers (both Shingate et al. 2020) on the most recent and high-quality genomes of Carcinoscorpius and one of the Tachypleus species. There is additional evidence therein that the Xiphosura WGD events are independent of the Arachnopulmonata WGD event.

Response:
Accept. We cited the reference (88, 89).

Response to reviewer#2:
A chromosome-level genome of the spider Trichonephila antipodiana reveals the genetic basis of its polygaphy and evidence of an ancient whole-genome duplication event
The 'Background' section do not really build up to the idea of polyphagy and detoxification abilities. Consequently, I am, as not being an expert in this field, not convinced about the idea that this species should have high detoxification abilities due to polyphagy, and that the detoxification related gene families actually are detoxification related gene families. The 'Background' is mainly a patchwork of more or less specific information about spiders. Immune system, sexual dimorphism, how many spider genomes etc does not really provide much useful information for this study. It

would be nice if the authors could collect information from other studies about food ranges (polyphagy), how that can be toxic, and what mechanisms/genes exist to overcome toxicity. If possible even couple it with genome duplication. That may enable them to come up with some hypotheses about what to find in this genome.
If the authors could provide an analysis including a number of other arthropods, that could demonstrate a correlation between food niche width and evolution of the gene families assumed to be involved in detoxification, it would strengthen their main conclusion a lot.

The whole-genome duplication has been shown before, so I believe that making this a major point of this study (even putting it in the title) is too much. Fair enough to provide data that confirms previous findings and extent the analyses with better data (chromosome level assembly).

Response:
Thank you for your suggestions. All of your suggestions have important guiding significance for us.
1) We changed the 'Background' and 'Conclusion' section.
2) We added some evidence about the association about polyphagy and detoxification. Such as the counts of P450 genes expressed in the whole body according to the RNA-data (Figure 7), the size of these gene families in some polyphagous, monophagous and oligophagous species (Table 5).
3) We reduced the part of whole-genome duplication analysis, and deleted the distribution analysis of pairwise synonymous substitution rates (Ks).

-line 43: production of silk - they cite 'Recent Advances in Development of Functional Spider Silk-Based Hybrid Materials'

Response:
Accept. We cite a new reference (2).

-line 45: whole genome duplication - they cite a paper (that is not yet peer-reviewed) that do not show the whole-genome duplication event, while a 2017 paper showed just that (https://bmcbiol.biomedcentral.com/articles/10.1186/s12915-017-0399-x?utm_campaign=BMC40864B&utm_medium=BMCemail&utm_source=Teradata)

Response:
Accept. We changed the reference (7).

Line 52: + Stegodyphus mimosarum and Acanthoscurria geniculate (https://onlinelibrary.wiley.com/doi/full/10.1111/jeb.12780)

Response:
Accept. We changed and added the reference (13).

Line 61-62: Also Stegodyphus dumicola (https://www.mdpi.com/2073-4425/10/2/137)

Response:
Accept. We changed and added the reference (11).

Line 101: what muscle tissue? From a leg?

Response:
Yes, the muscle tissue obtained from the spider's leg.

Line 254: not in the spider lineage, but in the ancestor of spiders and scorpions.

Response:
Accept. We changed.

Line 302: I think Table 4 is wrong here.

Response:
Accept. We changed.

Powered by Editorial Manager® and ProduXion Manager® from Aries Systems Corporation

	Line 317: I only see 4 tables in the manuscript.<br><br>Response:<br>Accept. We changed.<br><br>Line 331: numbers of exons and introns per gene seem wrong.<br><br>Response:<br>Accept. We changed (line 346).<br><br>Line 378: I am not sure the ref12 demonstrate a powerful spider immune response?<br><br>Response:<br>Accept. We changed and add the new reference (13).<br><br>Line 460: right references?<br><br>Response:<br>Thanks for your suggestion. We cite the reference with two recent papers on the most recent and high-quality genomes of Carcinoscorpius and one of the Tachypleus species (94, 95).<br><br>Line 464: ref should be 77.<br><br>Response:<br>Accept. We changed and cited the right reference.
Additional Information:	
Question	Response
Are you submitting this manuscript to a special series or article collection?	No
**Experimental design and statistics**<br><br>Full details of the experimental design and statistical methods used should be given in the Methods section, as detailed in our Minimum Standards Reporting Checklist. Information essential to interpreting the data presented should be made available in the figure legends.<br><br>Have you included all the information requested in your manuscript?	Yes
**Resources**<br><br>A description of all resources used, including antibodies, cell lines, animals and software tools, with enough information to allow them to be uniquely identified, should be included in the	Yes

Methods section. Authors are strongly encouraged to cite Research Resource Identifiers (RRIDs) for antibodies, model organisms and tools, where possible.<br><br>Have you included the information requested as detailed in our Minimum Standards Reporting Checklist?	
**Availability of data and materials**<br><br>All datasets and code on which the conclusions of the paper rely must be either included in your submission or deposited in publicly available repositories (where available and ethically appropriate), referencing such data using a unique identifier in the references and in the “Availability of Data and Materials” section of your manuscript.<br><br>Have you have met the above requirement as detailed in our Minimum Standards Reporting Checklist?	Yes

Manuscript

Click here to access/download;Manuscript;Trichonephila_for_production.doc

A chromosome- level genome of the spider *Trichonephila antipodiana* reveals the genetic basis of its polyphagy and evidence of an ancient whole-genome duplication event

Zheng Fan[1], Tao Yuan[1], Piao Liu[1], Luyu Wang[1], Jianfeng Jin[2], Feng Zhang[2], Zhisheng Zhang[1*]

1. School of Life Sciences, Southwest University, Chongqing 400700, China.

2. Department of Entomology, College of Plant Protection, Nanjing Agricultural University, Nanjing, Jiangsu 210000, China

* Correspondence should be addressed to Z.Z. (zhangzs327@qq.com),
ORCID: Zhisheng Zhang 0000-0002-9304-1789

Abstract

Background: The spider *Trichonephila antipodiana* (Araneidae), commonly known as the batik golden web spider, preys on arthropods with body sizes ranging from approximately 2 mm in length to insects larger than itself (over 20–50 mm), indicating its polyphagy and strong dietary detoxification abilities. Although it has been reported that an ancient whole-genome duplication event occurred in spiders, lack of a high-quality genome has limited characterization of this event.

Results: We present a chromosome- level *T. antipodiana* genome constructed based on PacBio and Hi-C sequencing. The assembled genome is 2.29 Gb in size with a scaffold N50 of 172.89 Mb. Hi- C scaffolding assigned 98.5% of the bases to 13 pseudo-chromosomes, and BUSCO completeness analysis revealed that the assembly included 94.8% of the complete arthropod universal single-copy orthologs (n=1,066). Repetitive elements account for 59.21% of the genome. We predicted 19,001 protein-coding genes, of which 96.78% were supported by

transcriptome-based evidence and 96.32% matched protein records in the UniProt database. The genome also shows substantial expansions in several detoxification-associated gene families, including cytochrome P450 monooxygenases, carboxyl/cholinesterases, glutathione-*S*-transferases, and ATP-binding cassette transporters, reflecting the possible genomic basis of polyphagy. Further analysis of the *T. antipodiana* genome architecture reveals an ancient whole-genome duplication event, based on two lines of evidence: (1) large-scale duplications from inter-chromosome synteny analysis; (2) duplicated clusters of Hox genes.

Conclusions: The high-quality *T. antipodiana* genome represents a valuable resource for spider research and provides insights into this species' adaptation to the environment.

Keywords Hi-C, high- quality genome, whole-genome duplication, gene family analysis, cytochrome P450, ABC, CCE, GST, Hox

Data Description

Background

Spiders (Araneae) have a worldwide distribution, have conquered virtually all terrestrial environments, and exhibit considerable species richness. A total of 48,683 spider species have been described to date, classified into 4,175 genera and 128 families [1]. Spiders are notable with respect to their numerous distinctive characteristics, including the production of silk [2] and venom [3], prolonged milk provisioning [4], foraging behavior [5], sexual size dimorphism [6], and whole-genome duplications (WGDs) [7].

To date, the genomes of 11 species of spider have been published or are available in the NCBI database (Table 1), which offer unprecedented insights into the unique biology of these arthropods. For example, complex sets of venom and silk genes have been identified in the genomes of *Stegodyphus mimosarum*, *Acanthoscurria geniculata*, and *Trichonephila clavipes* (formerly *Nephila clavipes*) [8-10]. The role of DNA methylation in spider gene regulation has been demonstrated in the genome of *Stegodyphus dumicola* [11]. And components of the spider immune system were initially characterized with reference to the genome of *Parasteatoda tepidariorum*, *S. mimosarum* and *A. geniculata* [12,13].

The spider genomes tend to be difficult to sequence, assemble, and annotate owing to their large size, high heterozygosity and repeat content. To date, the genomes of only three species (*Stegodyphus dumicola*, *Dysdera silvatica* and *Argiope bruennichi*) have been sequenced based on long sequencing reads (PacBio or Nanopore), only one of which was assembled to the chromosome level [11,14,15]. Lacking of high quality genome data has severely hampered the spider deeply research. In this study, we combined Pacific Biosciences (PacBio) and high-throughput chromosome conformation capture (Hi-C) sequencing to produce a high-quality, chromosome-level reference genome for *Trichonephila antipodiana*, and describe the salient features of the *T. antipodiana* genome, focusing on genome assembly, annotation, and evolutionary analyses.

The batik golden web spider, *T. antipodiana* (Figure 1), one of the typical Nephilinae species in the family Araneidae, which is recorded from a number of countries, including Australia (Queensland), the Solomon Islands, New Guinea, The Philippines, and China (Hainan Island) [1, 16]. Recently, in addition to many taxonomic papers that have provided a clear

outline of species in the Nephilinae, numerous studies on this subfamily have focused on their silk characteristics and sexual size dimorphism [6, 17, 18]. The webs constructed by *T. antipodiana* are approximately 1.0 m in diameter and can deal with a large size range of any suitable prey, including various species of Araneae, Crustacea, Formicidae, Isoptera, Orthoptera, Diptera, Coleoptera, Lepidoptera, Hymenoptera, Odonata, and even small birds, which thereby indicates their polyphagy and strong detoxification abilities [16]. Furthermore, it has been reported that when recycling their orb webs, these spiders may also feed on adhering pollen grains or fungal spores via extraoral digestion [19].

The enzymic detoxification of xenobiotics in cell prefer to converse of a lipophilic, non-polar xenobiotic into a more water-soluble and therefore less toxic metabolite, which can then be eliminated more easily from the cell. Cytochrome P450 represents a superfamily of enzymes responsible for the phase 1 metabolism of drugs and foreign compounds, which involve in catalyze the monooxygenation of a diverse array of xenobiotic and endogenous compounds [20]. Carboxyl/cholinesterase (CCE) superfamily is composed of functionally diverse proteins that hydrolyze carboxylic esters, also play an important role in detoxification of exogenous compounds in the diet or in the environment [21]. Glutathione S-transferase (GST) involved in catalyze the conjugation of activated xenobiotics to an endogenous water-soluble substrate, such as reduced glutathione (GSH), UDP-glucuronic acid or glycine [22]. ATP-binding cassette transporters (ABC) protein family is one of the largest transporter families and the toxic metabolites can be transported out of the cell via the action of ABC transporters [23]. In insects, the size of xenobiotic detoxification gene families may be associated with the complexity of their diets [24]. For example, in Hymenoptera species, relatively few members of these families

in the honeybee *Apis mellifera* genome compared with the *Nasonia vitripennis*, which was thought to encounter a wider range of potentially toxic xenobiotics in their diet and habitat [25, 26]. To investigate the polyphagy and detoxification of this spider, we analyzed a selection of detoxification-associated gene families, including P450 monooxygenases, CCE, GST, and ABC.

WGD is a process of genome doubling that supplies raw genetic material and increases genome complexity. It can provide new genetic material that enables paralogous genes to undergo sub- or neo-functionalization, which can contribute to the rewiring of gene regulatory networks, morphological innovations, and, ultimately, organismal diversification. It has been reported that an ancient WGD event occurred in the common ancestor of spiders and scorpions. In spiders, the first evidence of a duplication event was detected in the genome of the house spider *P. tepidariorum*, as indicated by a high number of duplicated genes, including two clusters of Hox genes [7]. In view of the importance of the WGD event in spider, to gain more evidence in support of the WGD event, we performed synteny and Hox gene analyses in *T. antipodiana*.

The *T. antipodiana* reference genome described herein will lay a foundation for further research on the unique characteristics and functions of spiders.

Methods

Sample collection and sequencing

The female specimen of *T. antipodiana* used in this experiment was obtained from Beihai (Guangxi Province, China), which was stored at -80°C prior to DNA extraction. The spider, excluding the abdomen, was prepared for PacBio and Illumina whole-genome sequencing, and leg muscle tissue was used for Illumina transcriptome sequencing.

Genome sequencing was performed by Berry Genomics (Beijing, China). Genome DNA for PacBio and Illumina sequencing was isolated using a Qiagen Blood & Cell Culture DNA Mini Kit. PacBio Sequel II libraries for PacBio sequencing were constructed with insert sizes of 20 kb using a SMRTbell™ Template Prep Kit 1.0-SPv3. Two short paired-end insert libraries containing 350-bp sequences were constructed for survey analysis using a Truseq DNA PCR-free kit and sequenced using the NovaSeq 6000 platform.

For the purposes of Hi-C sequencing, the muscle tissues of the single female specimen were fixed with formaldehyde and lysed, and the cross-linked DNA was subsequently digested overnight with *Mbo*I. Sticky ends were biotinylated and proximity-ligated to form chimeric junctions that were enriched for and then physically sheared to a size of 350 bp. Chimeric fragments representing the original cross-linked long-distance physical interactions were then processed into paired-end sequencing libraries, and 150-bp paired-end reads were generated using the Illumina HiSeq PE150 platform.

Muscle RNA was extracted using TRIzol (Invitrogen) according to the manufacturer's instructions.

Genome survey and assembly

Quality control of the raw Illumina data was performed using BBTools suite v38.67 (Bestus

Bioinformaticus Tools, RRID:SCR_016968) [27]. The duplicates were removed using "clumpify.sh," and then "bbduk.sh" was used to trim the reads ends to Q20 with reads shorter than 15 bp or with >5 Ns. The poly-A/G/C tails of at last 10 bp were trimmed, and the overlapping paired reads were corrected using "bbduk.sh." All filtered reads were used to estimate genome size and other characteristics. In addition, a 21-mer was selected for k-mer analysis and the k-mer distribution was estimated using "khist.sh" (BBTool). The 21-mer depth frequency distribution was calculated using GenomeScope v1.0.0 (GenomeScope, RRID:SCR_017014) [28], and the maximum k-mer coverage cutoff was set to 10,000.

For the long reads generated using the PacBio Sequel platform, contig assembly of the *T. antipodiana* genome was conducted using Flye v2.5 (Flye, RRID:SCR_017016) [29] with a single round of polishing and the minimum overlap between reads was set to 3,000. Heterozygous regions of the assembly were removed using Purge Haplotigs v1.1.0 [30], with a 50% cutoff for identifying contigs as haplotigs. Illumina reads were used to polish the assembly using NextPolish v1.0.5 [31] over two rounds. During all the Flye and NextPolish polishing steps, Minimap2 v2.12 (Minimap2, RRID:SCR_018550) [32] was used as the read aligner.

The Hi-C reads were used to generate a chromosome-level assembly of the genome, and three software packages were used for analysis. The reads were initially subjected to quality control to removing the duplicates and then aligned to the genome using Juicer v1.6.2 (Juicer, RRID:SCR_017226) [33]. The resulting alignment BAM file was then transformed to a BED format and fed to SALSA v2.2 [34] to correct the obvious misjoin errors between contigs. The alignment BAM file was also mapped to the cleaned assembly data using Minimap2. Finally, the data were fed to Allhic v0.9.13 [35] to anchor contigs to chromosomes.

Potential contaminant sequences were inspected using HS-BLASTN [36] and BLAST+ (blastn) v2.7.1 [37] against the NCBI nucleotide (nt) and UniVec databases.

Genome completeness was assessed using the Benchmarking Universal Single-Copy Orthologs (BUSCO) v3.0.2 pipeline (BUSCO v3.0, RRID: SCR_015008) [38] against an arthropod reference gene set using the arthropoda_odb 9 database of the genome (n = 1,066). To evaluate the mapping rate, the clean reads of the Illumina or PacBio sequences were mapped to the reference genome using Minimap2.

Genome annotation

Genome annotation essentially encompasses four aspects: repeat, protein-coding gene, non-coding RNA, and gene function annotations.

We searched for repetitive elements in the assembled genome by a combination of ab-initio and homology-based searching. Initially, we constructed a specific repeat database using RepeatModeler v2.0.1 (RepeatModeler, RRID:SCR_015027)) [39], and thereafter combined the an-initio database and known repeat library (Repbase) [40] as the reference repeat database. To identify repetitive elements, we used RepeatMasker (RepeatMasker, RRID:SCR_012954) [41] to search against the reference repeat database. Non-coding RNAs were identified using Infernal v1.1.2 (Infernal, RRID:SCR_011809) [42] and tRNAscan-SE v2.0.6 (tRNAscan-SE, RRID:SCR_010835) [43], and tRNAs of high confidence were confirmed using the tRNAscan-SE script “EukHighConfidenceFilter”.

Using the repeat-masked genome, we used Maker v2.31.10 (Maker, RRID:SCR_005309) for genome annotation by integrating ab initio, transcriptome-based, and protein homology-

based evidence [44]. Augustus v3.3.2 (AUGUSTUS, RRID:SCR_008417) [45] and GeneMark-ES/ET/EP v4.48_3.60_lic [46] were used for ab-initio gene prediction. To accurately model the sequence properties, both gene finders were initially trained using the BRAKER v2.1.5 pipeline (BRAKER, RRID:SCR_018964) [47], which makes use of the mapped transcriptome sequence data. Previously, RNA-seq data were mapped to our genome assembly using HISAT2 v 2.2.0 (HiSat2, RRID:SCR_015530) [48]. BRAKER was then run with default parameters. The RNA-seq data were further assembled into transcripts using Stringtie v2.1.3 [49], with the assembled genome used as a reference. The resulting transcripts were provided as input for Maker via the "est" option. The protein sequences of *Drosophila melanogaster* (GCA_000001215.4), *Ixodes scapularis* (GCA_002892825.2), *Stegodyphus mimosarum* (GCA_000611955.2), *Trichonephila clavipes* (GCA_002102615.1), *Parasteatoda tepidariorum* (GCA_000365465.3), *Strigamia maritima* (GCA_000239455.1), and *Daphnia pulex* (GCA_900092285.2) were downloaded from the NCBI database as protein homology-based evidence required by Maker.

The functions of the predicted protein sequences were assigned against the UniProtKB/Swissprot database using Diamond v0.9.24 (Diamond, RRID:SCR_016071) [50] with a more sensitive mode, one maximum number of target sequences, to report alignments with an e-value threshold of 1e-5.

Annotation of the protein domains was based on Gene Ontology (GO) and Reactome pathways of the predicted protein-coding genes, with InterProScan v5.41-78.0 (InterProScan, RRID: SCR_005829) [51] being used to screen proteins against the following five databases: Pfam [52], Panther [53], Gene3D [54], Superfamily [55], and CDD [56].

Using eggNOG-mapper v2.0 [57], the eggNOG v5.0 database [58] was used for GO, EC (expression coherence), KEGG (Kyoto Encyclopedia of Genes and Genomes) pathways, KEGG orthologous groups (KOs), and COG (clusters of orthologous groups) functional category annotation of the predicted protein-coding genes.

To assess the completeness of the *T. antipodiana* protein annotation, we used the protein mode of the BUSCO v3.0.2 ((RRID:SCR_015008) pipeline and the arthropod reference set of arthropoda_odb 9 (n = 1,066) [38].

Phylogenetic analyses and GO/KEGG enrichment analyses

Orthologous gene clusters were classified using OrthoFinder v2.3.8 (OrthoFinder, RRID:SCR 017118) [59] across the well-annotated and well-assembled genomes of 10 species covering representative Chelicerata lineages along with *T. antipodiana*: one Scorpiones (*Centruroides sculpturatus*, GCA_000671375.2); five Acari (*Dermatophagoides pteronyssinus*, GCA_001901225.2; *Galendromus occidentalis*, GCA_000255335.1; *Tetranychus urticae*, GCA_000239435.1; *Varroa destructor* GCA_002443255.1; *I. scapularis*, GCA_002892825.2); three Araneae (*Parasteatoda tepidariorum*, GCA_000365465.3; *Stegodyphus mimosarum*, GCA_000611955.2; *T. clavipes* GCA_002102615.1); and one Xiphosura (*Tachypleus tridentatus*). With the exception of *T. tridentatus* (doi:10.5061/dryad.68pk1rv), most protein sequences were downloaded from the NCBI database.

To infer the phylogeny of these species, the protein sequences of 236 single-copy genes were separately aligned using MAFFT v7.394 (MAFFT, RRID:SCR_011811) [60] based on the L-INS-I strategy. The resulting alignments were trimmed using trimAl v1.4.1 (trimAl,

RRID:SCR_017334) [61] to remove sites of unclear homology using the heuristic method "automated1." The resulting alignments were concatenated using FASconCAT-G v1.04 [62]. Genes that violated the models were removed prior to tree inference. Finally, ML reconstructions were performed using IQ-TREE v2.0.7 (IQ-TREE, RRID:SCR_017254) [63] with extended model selection followed by tree inference, model set by LG, with the number of partition pairs for the rcluster algorithm, replicates for ultrafast bootstrap, and SH approximate likelihood ratio tests being 1000, 10, and 1000, respectively.

The divergence time was estimated with MCMCTree within the package PAML v4.9j (PAML, RRID:SCR_014932) [64] using parameters with independent clock rates; BDparas-related birth, death, and sampling rates of 1, 10, and 0.1, respectively; kappa_gamma of 62; alpha_gamma of 11; rgene_gamma of 2201; and sigma2_gamma of 1101. Fossil records were derived from the paleobiodb database (https://paleobiodb.org/) and the recently described fossils *Eramoscorpius brucensis* [65] and *Parioscorpio venator* [66], with Chelicerata (genus *Paleomerus*, 516–541 Mya), Parasitiformes (*Deinocroton draculin*, 93.5–145.5 Mya) and Arachnopulmonata (*E. brucensis* and *P. venator*, 435–439 Mya).

Café v4.2.1 (CAFÉ, RRID:SCR_005983) [67] was employed to identify the likelihood of gene family expansion and contraction using the single birth–death parameter lambda and a P-value threshold of 0.01. GO and KEGG functional enrichment of the significantly expanded families was assessed using Tbtools v1.045 [68].

Annotation of dietary detoxification-related gene families

To manually annotate the genes of detoxification-related enzymes (P450s, CCEs, GSTs, and

ABCs), we initially downloaded the amino acid sequences of the P450s, CCEs, GSTs, and ABCs predicted from the *D. melanogaster*, *Bombyx mori*, and *T. urticae* sequences obtained from NCBI.

For cytochrome P450 proteins, we performed a blastp-like search using MMsesqs2 v11 [69] with four rounds of iteration, as the identity between two proteins can be as low as 25%. Using the Pfam database, Interproscan v5.41-78.0 (Interproscan, RRID:SCR_005829) [70] was used to confirm specific conserved domains of the P450 sequences. And every P450 protein was checked the struction including four-helix bundles (D, E, I and L), helices J and K, two sets of β sheets, and a coil referred to as the "meander". The regions comprise a heme-binding loop, a strictly conserved Glu-X-X-Arg motif in helix K, and a consensus sequence (Ala/Gly-Gly-X-Asp/Glu-Thr-Thr/Ser) in the central part of helix I [71]. We deleted the invalid matches of the proteins using MMsesqs2 with a tblatn-like search, and each protein was also examined to identify intron/exon boundaries.

Members of the other three detoxification enzyme gene families (CCEs, GSTs, and ABCs) of *T. antipodiana* were identified using MMsesqs2 v11 using a blastp-like search with four rounds of iteration and an e-value of 0.001. Interproscan v5.41-78.0 (Interproscan, RRID:SCR_005829) was used to confirm the specific conserved domains of genes using the Pfam database. Classification and functional categories of the resulting HMMER-Pfam bellow were further checked using an online NCBI BLASTP of the non-redundant (nr) GenBank protein database. Each protein was assessed for intron/exon boundaries, and extremely short or long sequences were removed. Finally, the multi-hits were reduced to the same gene region and we deleted the invalid matches of the proteins using MMsesqs2 with a tblatn-like search.

We also conducted an analysis of the sequence evolution of the specific gene families such as cytochrome P450, CCE, GST, and ABC. Initially, the proteins were aligned using MAFFT v7.450 with common parameters, after which the resulting alignments were trimmed using trimAl v1.4.1 to remove the sites with unclear homology based on the heuristic method "automated1." Finally, gene trees were constructed using IQ-TREE v2.0.7 with an LG model and 1000 ultrafast bootstrap replicates.

In order to obtain the P450 gene expression in the whole body of *T. antipodiana*, we count the number of P450 gene from the RNA data by the software of FeatureCounts [72]. RNA-seq data were mapped to our genome assembly using HISAT2 v 2.2.0 previously.

WGD analyses

It has been reported that an ancient WGD event occurred in the common ancestor of spiders and scorpions, and in an attempt to confirm the occurrence of this event, we examined two possible lines of evidence.

We conducted an intra-specific analysis of the synteny between *T. antipodiana* chromosomes. *T. antipodiana* proteins were searched against themselves with MMsesqs2 v11 using a blastp-like search with three rounds of iteration and an e-value of 0.001. The blast results and gene annotation GFF3 file were fed to MCScanX [73] with an e-value threshold of 1e-8. A collinear block was defined by a homologous region shared by four or more gene sequences with no rearrangements.

In arthropods, 10 highly conserved Hox genes that are inferred to occur in the common ancestor of Panarthropoda play important roles [74]. In the present study, we manually annotated the Hox

genes of *T. antipodiana*, using the Hox protein amino acid sequences predicted for *Daphnia magna*, *Parasteatoda tepidariorum*, *Centruroides sculpturatus*, *I. scapularis*, and *D. melanogaster* downloaded from the NCBI database. MMsesqs2 v11 was used to perform a blastp-like search for four rounds of iteration with an e-value of 0.001. The Hox gene clusters classification and functional categories of the resulting BLAST bellow were further assessed using the HomeoDB database [75].

The locations of the Hox genes were further confirmed based on genome annotation, and Hox gene clusters and synteny blocks were plotted across chromosomes using Tbtools.

Results and Discussion

A high-quality genome among Araneae

In this study, we constructed a chromosome- level *T. antipodiana* genome based on PacBio and Hi-C sequencing.

Sequencing yielded 767.07 Gb of clean data, comprising 305.96 Gb Illumina (133×), 235.79 Gb PacBio (103×), 215.05 Gb Hi-C (94×), and 10.27 Gb transcriptome reads. The long PacBio subreads had mean N50 lengths of 14.81 kb and 21.19 kb, respectively. The detailed sequencing data are summarized in Table 2.

K-mer analysis indicated that the number of unique k-mers peaked at 21 and predicted a genome assembly size of 2.15 Gb (Figure S1), which is in general agreement with the recent draft genome of *T. clavipes* (2.44 Gb).

Using the Flye assembler, we obtained an initial 2.38 Gb genome assembly with a contig

N50 of 1.17 Mb. To enhance the draft assemblies, haplotigs and contig overlaps were removed from the genome. The total length of the assembly was 2.31 Gb, with a contig N50 of 1.23 Mb. Finally, Hi-C data were used for genome scaffolding with a mapping rate of 89.16%, and a high-quality chromosome-level genome assembly of *T. antipodiana* was accordingly obtained with a total length of 2.29 Gb, a contig N50 of 1.14 Mb, and a scaffold N50 of 172.89 Mb (Table 3). The genome of *T. antipodiana* is one of the two chromosome-level genomes obtained for spiders to date, the other being that of *A. bruennichi* [15]. A comparison of the genome assembly obtained in the present study with that of the congeneric species *T. clavipes*, indicated the superior quality of the *T. antipodiana* assembly, with a scaffold N50 of 172 Mb compared with that of 62.96 kb obtained for *T. clavipes* (Table 1).

BUSCO is a tool used to assess the completeness of genome/transcriptome assemblies and annotated proteins based on single-copy orthologs, and the BUSCO results obtained in the present study indicated that 967 (94.8%) of the 1,066 orthologs in a reference arthropod data set (arthropoda_odb9) were labeled as complete in our assembly, which is similar to the value obtained for *T. clavipes* (94.85%). The results of BUSCO analysis at all steps in the assembly of the *T. antipodiana* are shown in Table 3.

The mapping rate, which is defined as the proportion of high-throughput sequencing reads that are uniquely mapped to a reference genome, reflects the accuracy of the assembly, and in the present study, we obtained mapping rates of 96.78%, 97.23%, and 97.61% for the RNA-seq, Illumina, and PacBio reads, respectively.

Gene annotation

The *T. antipodiana* genome comprises 59.21% repetitive elements, including 57.12% transposable elements (TEs), 0.72% small RNAs, 0.13% satellites, 1.08% simple repeats, and 0.19% low-complexity regions (Table 4). The TEs are predominantly represented by five categories of abundant repeats, unclassified (22.08%), DNA transposon elements (22.42%), long interspersed elements (LINEs, 3.61%), long terminal repeats (LTRs, 3.45%), and short interspersed elements (SINEs, 1.10%). An analysis of the distribution of repetitive elements in the *T. antipodiana* genome, revealed that DNA transposon elements are highly distributed in the genome regions (Figure 2), with TcMar-Tc1 and hAT-Charlie being identified as the most common DNA transposons elements, accounting for 7.18% and 6.19%, respectively. We found that the percentage of DNA transposons elements in *T. antipodiana* is higher than that in some other species of spider, including *Argiope bruennichi* (6.27%), *Trichonephila clavipes* (13.71%), *Araneus ventricosus* (14.45%), *Dysdera silvatica* (19.58%), *Stegodyphus dumicola* (16.17%), *Stegodyphus mimosarum* (18.77%), *Pardosa pseudoannulata* (16.55%), *Loxosceles reclusa* (10.23%), *Anelosimus studiosus* (7.94%), *Latrodectus hesperus* (7.03%), and *Parasteatoda tepidariorum* (6.9%) [15].

Using the MAKER2 genome annotation tool, we identified 19,001 protein-coding genes in the *T. antipodiana* genome, with a mean number of 7.24 exons and 6.12 introns per gene, and mean exon and intron lengths of 247.46 bp and 3.73 kb, respectively. On the basis of BUSCO analysis, we identified 1,027 (96.3%) complete, 60 (5.6%) duplicated, 14 (1.3%) fragmented, and 25 (2.4%) missing orthologs. Furthermore, we found that a total of 18,303 (96.33%) genes had at least one record in the SwissProt or TrEMBL databases. InterProScan and EggOG analyses identified the protein domains for 14,705 (77.39%) genes, 12,226 GO

terms, 9,465 KEGG ko terms, 5,788 KEGG pathways, 14,325 COG categories, and 3,183 Enzyme Codes. Comparatively, 22,689 protein-coding genes have been identified in the *T. clavipes* genome, which approximately comparable to the number in *T. antipodiana* (Figure 3a).

We identified 4,452 ncRNA-associated loci in the squid sequencing data, and found that all the essential and well-conserved metazoan ncRNAs are also present in the *T. antipodiana* genome: 3,653 tRNAs, 160 ribosomal RNAs (rRNAs) (5S, 5.8S, SSU, and LSU), 2 RNase P, 1 RNase MRP, 22 SRP, 216 major spliceosomal snRNAs (U1, U2, U4, U5, U6), 26 minor spliceosomal snRNAs (U11, U12, U4atac, and U6atac), and 6 CD-boxes.

Gene orthology and comparative analysis with other genomes

Identifying homologous relationships among the sequences of different species plays a pivotal role in enhancing our understanding of evolution and diversity. In this regard, we compared the protein-coding genes of *T. antipodiana* with those of 10 representative Arachnida species, including three species of spider (*Parasteatoda tepidariorum*, *Stegodyphus mimosarum*, and *T. clavipes*), one Scorpiones (*Centruroides sculpturatus*), and five Acari (*Dermatophagoides pteronyssinus*, *Galendromus occidentalis*, *Tetranychus urticae*, *Varroa destructor*, and *Ixodes scapularis*) to identify orthologous groups, with *Tachypleus tridentatus* being used as an outgroup. Using OrthoFinder, we obtained a total of 203,348 genes among the 11 species, which were clustered into 20,785 orthogroups. We also count the genes of single-copy and multi-copy orthologs, common genes unique to Araneae, species-specific genes, and other unassigned orthologous genes among the 11 species (Figure 3a). Gene family analysis also revealed that among these species, 152 gene families and 590 genes were unique to *T. antipodiana*.

To gain an understanding of Arachnida genomic evolution, we reconstructed a phylogenomic tree of the 11 assessed species based on 236 single-copy orthologous genes, which were calibrated using four fossil records. The phylogenomic tree obtained indicated that Scorpiones (*C. sculpturatus*) show a close relationship with spiders, and we estimated that *T. antipodiana* and *T. clavipes* diverged approximately 16.15 to 19.62 Mya (Figure 3a).

Gene family evolution and GO/KEGG enrichment analyses

Within the *T. antipodiana* genome, we identified 1,186 expanded and 2,480 contracted gene families ($p \leq 0.01$), among which 300 and 143 families have undergone significant expansions and contractions ($p < 0.001$), respectively (Figure 3a). In Figure 3b, we show the 20 families that have undergone the largest expansions.

Among the gene families showing varying degrees of expansion, there are a number that play vital roles in spiders' survival, including those related to immunity, dietary digestion, and detoxification. The expansion of immunity-related gene families, such as putative peptidases, immunoglobulin I-set domain, and retroviral aspartyl proteases, reflects the powerful innate immune response of spiders [12, 13], whereas certain digestion- and detoxification-related gene families, such as cytochrome P450s, peptidases, and proteases, may reflect mechanisms underlying the wide dietary repertoire of the spider *T. antipodiana*. For example, members of the cytochrome P450 family play important roles in digestion and detoxification by contributing to xenobiotic metabolism and insecticide resistance [68]. Given its large webs and diverse range of prey items, it is essential for *T. antipodiana* to have effective digestion and detoxification systems, and gene ontology (GO) and KEGG pathway enrichment analyses of these expanded

genes further confirmed this hypothesis.

Among the GO enrichment results, we noted certain important functions associated with the regulation of hormone levels, oxidoreductase activity, structural constituent of the cuticle, and metabolic and catabolic processes (including hormone, steroid, isoprenoid, and ecdysteroid metabolic processes). The enrichment of these metabolic and catabolic processes is again consistent with the strong detoxification ability of *T. antipodiana* (Figure 4).

Among the KEGG enrichment results (Figure 5), we identified a number of important functions, including cell proliferation and differentiation (such as cancer-related, hedgehog signaling, and notch signaling pathways), biosynthesis, and metabolism (such as linoleic, arachidonic, and drugs) that are consistent with the GO enrichment results. We also detected strong enrichment of drug and xenobiotic metabolism by cytochrome P450.

Analysis of detoxification-related gene families in *T. antipodiana*

Numerous families of genes, including P450s, GSTs, ABCs, and CCEs, play roles in the detoxification of toxic compounds, and these genes have most likely evolved in relation to polyphagous species (Table 5). In the polyphagous species (such as spider mite *T. urticae*, *Spodoptera frugiperda*, *Tribolium castaneum*, *Spodoptera litura*, *Helicoverpa armigera*, *Trialeurodes vaporariorum*), the number of these genes showed a great expansion [77–83]. While in monophagous or oligophagous species (such as *Bombyx mori*, *Pediculus humanus humanus*), the expansions of these gene families are rarely observed [77, 83–85]. For further analysis of the detoxification ability of *T. antipodiana*, we manually annotated the genes of detoxification-related enzymes (P450s, CCEs, GSTs, and ABCs).

From the perspective of xenobiotic metabolism, P450s are the most important superfamily of enzymes in arthropods [86]. In the genome of *T. antipodiana*, we identified 167 CYP genes, comprising four major classes: CYP2 (57 genes), mitochondrial P450 (19), CYP3 (43), and CYP4 (48). Among insects, the numbers of P450 genes to some extent reflect the adaptation and pesticide resistance (Table 5). For example, in some polyphagous species such as the red flour beetle, *Tribolium castaneum* (Coleoptera) and three moths, *Spodoptera litura*, *S. frugiperda* and *Helicoverpa armigera* (Lepidoptera), the number of P450 genes shows a great expansion with 143, 138, 425 and 114 genes identified, respectively. While in some monophagous or oligophagous species, these expansions are rarely observed, such as Lepidoptera (*Bombyx mori*) for the number of P450 genes with 46 and 81.

Compared with other arthropods, the number of genes of every class in commonly used model species, such as *D. melanogaster*, show varying degrees of increase (Figure 6). We can see the CYP genes of *T. antipodiana*, CYP2 clade genes showed a remarkable expansion. CYP2 enzymes are associated with detoxification and/or bioactivation of certain foreign chemicals [86]. Similar results have been obtained for polyphagous specie *T. urticae*, revealing 81 CYP genes with a notable lineage-specific expansion of duplicated intron-less CYP2 clade genes [78]. With regards to *T. antipodiana*, it is conceivable that the expansion of the CYP2 clade may be associated with its polyphagous habit.

In these polyphagous species of Coleoptera and Lepidoptera, the CYP3 and CYP4 clade genes of P450 showed expansion (Table 5). And the number of genes in CYP3 and CYP4 clade in *T. antipodiana* also showed a great expansion. The CYP3 clade genes have been found to be associated with xenobiotic metabolism and insecticide resistance when induced by

phenobarbital, pesticides, or natural products, whereas certain clade CYP4 genes, the least studied among the insect CYP genes, can be induced by xenobiotics as metabolizers, and other are linked to odorant or pheromone metabolism. In insects, it has been reported that the mitochondrial P450 clade is associated with insecticide resistance [86]; for example, the CYP12A1 gene of the housefly been shown to play a role in the metabolism of xenobiotics, although not insect ecdysteroids. Moreover, it has been reported that exposure to cadmium toxicity increases expression of cytochrome P450-encoding genes in the wolf spider *Pirata subpiraticus* [87].

In addition, the inducing changes in gene expression of the detoxification-related genes provide polyphagous arthropods greater fitness on a specific host. For example, if changed the *T. urticae* from the optimal host (bean) to a challenging host (tomato), transcriptional responses increased with whole-scale changes [76]. We also analyzed the P450 genes expression in the female *T. antipodiana* by RNA-Seq, and the expression genes was shown in Figure 7.

The CCE superfamily comprises a functionally diverse group of proteins that hydrolyze carboxylicesters [21]. CCEs not only regulate endogenous compounds (such as hormones, pheromones, and acetylcholine) but also detoxify exogenous compounds derived from dietary or environmental sources. These genes have been categorized into three main phylogenetic classes, namely, hormone/semiochemical processing, dietary/detoxification, and neuro/developmental functions. Within the *T. antipodiana* genome, we identified 48 CCE genes, among which, the overwhelming majority (47) belong to neuro/developmental class, with the single remaining gene belonging to the hormone/semiochemical class (Figure S2). Notably, whereas in the fruit fly *D. melanogaster,* the number of CCEs in the neuro/developmental class

is relatively conserved, we detected a clear expansion in the *T. antipodiana* genome (Figure S2), thereby reflecting the difference between spiders and insects.

GSTs play roles in cellular detoxification by catalyzing nucleophilic attack of the tripeptide glutathione (GSH) in the electrophilic centers of xenobiotic and endobiotic compounds [88]. Within the *T. antipodiana* genome, we identified 22 GST genes, and phylogenetic analyses of the cytosolic *T. antipodiana* GSTs revealed five different classes of these genes (Figure S3), namely, Delta/Epsilon (2 genes), Mu (15), Theta (1), Sigma (2), and Zeta (2), among which the Mu class is the largest and shows considerable expansion in *T. antipodiana*. Functionally, the Mu GSTs have been reported to participate in the oxidative stress response associated pesticide resistance in *T. urticae* [89].

The ABCs can act directly on toxicants as primary-active transporters, thereby protecting cells or organisms [23]. The genome of *T. antipodiana* was found to contain 48 ABC genes belonging to seven different classes (Figure S4): ABCA (11 genes), ABCB (12), ABCC (11), ABCD (3), ABCE (1), ABCF (3), ABCG (6) and ABCH (1). Among the annotated genomes of arthropod species that have been studies in detail, that of *T. urticae* has been found to contain the largest number of ABC genes (103), followed by that of *T. castaneum* (73) and *D. pulex* (65), whereas the genome of *A. mellifera* has only 41 ABC genes.

Analysis of the *T. antipodiana* genome provides evidence in supports of a WGD event

On the basis of our analysis of the *T. antipodiana* genome, we provide two line of evidence in support of the hypothesis that an ancient WGD probably occurred after the divergence of the common ancestor of spiders and scorpions from other arachnid lineages (mites, ticks, and

harvestmen) prior to 430 Mya [90–92], which occurred independently of the apparent WGD that is evident in all extant *horseshoe crabs* [93, 94].

First, synteny analysis revealed the occurrence of certain segmental duplications, the signatures of which are suggestive of a WGD. These signatures were observed in multiple chromosomes, such as chromosomes 2, 3, 9 and 10 (Figure 1). These results are comparable with the findings of a similar analysis of the *P. tepidariorum* genome [95]. The conservation of synteny within the genome of *T. antipodiana* supports the hypothesis of a WGD event.

And, we detected two clusters of Hox genes. Variation in the number of Hox gene clusters is considered to be consistent the occurrence of WGD events during the course of evolution [66]. In the present study, we identified Hox genes of the following classes in the *T. antipodiana* genome: *lab*, *pb*, *Hox3*, *Dfd*, *Scr*, *ftz*, *Antp*, *Ubx*, *abdA*, and *AbdB*. One complete HOX cluster copy was identified on chromosome 12, whereas a further HOX cluster detected on in chromosome was found to be lacking copies of *Hox3*, *ftz*, *ubx*, and *Abd-a* genes (Figure 2). Notably, however, we detected two copies of nearly all the Hox genes in the *T. antipodiana* genome, thereby indicating that entire Hox clusters have been duplicated. The results are consistent with those obtained in a previous study on the house spider *P. tepidariorum* [7].

Conclusion

A high-quality chromosome-level genome for the spider *Trichonephila antipodiana* was assembled, which is the second chromosome-level spider genome. The polyphagy of this species is highly related with P450s gene families. The large-scale inter-chromosomal duplications and duplicated clusters of Hox genes highlights the WGD event during the

evolution of spiders. The high-quality genome assembled here provides more useful data for studies on the evolutionary adaptations of spiders and species-specific functions.

Figure Legends

Figure 1. Habitus of *Trichonephila antipodiana,* female.

Figure 2. Schematic representation of the genomic characteristics of *Trichonephila antipodiana*. The inner ring of the circle is based on the findings of inter-chromosome synteny analysis; The outer ring of the circle represents the distribution of genes, GC content, DNA elements, long interspersed elements (LINEs), long terminal repeats (LTRs), short interspersed elements (SINEs), and chromosomes. The location of Hox genes is marked on the outer ring of the chromosome circle.

Figure 3. Phylogenetic and comparative gene family analyses of *Trichonephila antipodiana* and other Arachnida species. The estimated species divergence times (millions of years ago; MYA) are indicated at each branch point. Node values indicate gene families showing expansion (red), contraction (green), and rapid evolution (black in bracket). The bar chart indicates the number of genes classified into six groups (single-copy, multi-copy, species-specific, unassigned, other, and common genes unique to Araneae).

Figure 4. GO annotation of the top 20 expanded gene families.

Figure 5. KEGG annotation of the top 20 expanded gene families.

Figure 6. Expansion of the P450 gene family in *Trichonephila. antipodiana*. The phylogenetic tree shows the orthologous and paralogous relationships of all P450 genes from *T. antipodiana* and *Drosophila melanogaster*. Bootstrap values are indicated on the nodes.

Figure 7. The heatmap of P450 genes expression in the female *T. antipodiana* by RNA-Seq.

Availability of Supporting Data and Materials

All raw sequencing data and the genome assembly of *T. antipodiana* are available at the National Center for Biotechnology Information (NCBI) under the Bioproject ID PRJNA627506. Other data supporting this work are openly available in the *GigaScience* repository, GigaDB [96].

Additional Files

Figure S1. k-mer distribution of the *Trichonephila antipodiana* genome.

Figure S2. Expansion of the CCE gene family in *Trichonephila antipodiana*. The phylogenetic tree shows the orthologous and paralogous relationships of all CCE genes from *T. antipodiana* and *Drosophila melanogaster*. Bootstrap values are indicated on the nodes.

Figure S3. Expansion of the GST gene family in *Trichonephila antipodiana*. The phylogenetic tree shows the orthologous and paralogous relationships of all GST genes from *T. antipodiana* and *Drosophila melanogaster*. Bootstrap values are indicated on the nodes.

Figure S4. Expansion of the ABC gene family in *Trichonephila antipodiana*. The phylogenetic tree shows the orthologous and paralogous relationships of all ABC genes from *T. antipodiana* and *D. melanogaster*. Bootstrap values are indicated on the nodes.

Abbreviations

WGD: whole genome duplications; BUSCO: Benchmarking Universal Single-Copy Orthologs; Hi-C: High-throughput chromosome conformation capture; PacBio: Pacific Biosciences; P450s:

P450 monooxygenases; CCE: carboxyl/cholinesterases; GST: glutathione-*S*-transferases; ABC: ATP-binding cassette transporters; TEs: transposable elements; LINEs: long interspersed elements; LTRs: long terminal repeats; SINEs: short interspersed elements; KEGG: : Kyoto Encyclopedia of Genes and Genomes; GO: Gene Ontology; ECs: expression coherence; Kos: KEGG orthologous groups; NCBI: National Center for Biotechnology Information; KDEs: kernel density estimates; Ks: pairwise synonymous substitution rates; Hox genes: homeotic genes.

Competing Interests

The authors declare that they have no competing interests.

Authors' Contributions

Z.F. performed the major part of data analysis and drafted the manuscript. L.W. T.Y. and P.L. contributed to sample collection. J.J. and F.Z. contributed to data analysis and edits to the manuscript. Z.Z. contributed to research design and final edits to the manuscript. All authors read and approved the final manuscript.

Acknowledgements

This research is funded by the Natural Science Foundation of Chongqing (No. cstc2019jcyj-zdxmX0006), the Investigation Project of Basic Science and Technology (No. 2018FY100305).

Reference

1 World Spider Catalog (2020). World Spider Catalog. Version 21.5. Natural History Museum Bern, online at http://wsc.nmbe.ch, accessed on {July 26, 2020}. doi: 10.24436/2.

2 Kluge JA, Rabotyagova U, Leisk GG, Kaplan DL. Spider silks and their applications. Trends in Biotechnology. 2008; **26**(5): 244–251.

3 Saez NJ, Herzig V. Versatile spider venom peptides and their medical and agricultural applications. Toxicon. 2019; 158: 109–26.

4 Chen ZQ, Corlett RT, Jiao XG, et al. Prolonged milk provisioning in a jumping spider. Science. 2018; 362:1052–5.

5 Welch KD, Haynes KF, and Harwood JD. Prey-specific foraging tactics in a web-building spider. Agricultural and Forest Entomology. 2013; **15**(4): 375–381.

6 Kuntner M, Coddington JA. Sexual Size Dimorphism: Evolution and Perils of Extreme Phenotypes in Spiders. Annu Rev Entomol. 2020; 65: 57–80.

7 Schwager EE, Sharma PP, Clarke T, et al. The house spider genome reveals an ancient whole-genome duplication during arachnid evolution. BMC Biology. 2017; 15: 62.

8 Gendreau KL, Haney RA, Schwager EE, et al. House spider genome uncovers evolutionary shifts in the diversity and expression of black widow venom proteins associated with extreme toxicity. BMC Genomics. 2017; 18: 178.

9 Sanggaard KW, Bechsgaard JS, Fang X, et al. Spider genomes provide insight into composition and evolution of venom and silk. Nature communications. 2014; 5: 3765.

10 Babb PL, Lahens NF, Correa-Garhwal SM, et al. The *Nephila clavipes* genome highlights the diversity of spider silk genes and their complex expression. Nature genetics. 2017;

49(6): 895–903.

11 Liu S, Aageaard A, Bechsgaard J, Bilde T. DNA Methylation Patterns in the Social Spider, *Stegodyphus dumicola*. Genes (Basel). 2019; **10**(2): 137.

12 Palmer WJ and Jiggins FM. Comparative Genomics Reveals the Origins and Diversity of Arthropod Immune Systems. Molecular biology and evolution. 2015; **32**(8): 2111–2129.

13 Bechsgaard J, Vanthournout B, Funch P, et al. Comparative genomic study of arachnid immune systems indicates loss of beta-1,3-glucanase-related proteins and theimmune deficiency pathway. Journal of Evolutionary Biology. 2016; **29**(2): 277–291.

14 Sanchez Herrero JF, Frias Lopez C, Escuer P, et al. The draft genome sequence of the spider *Dysdera silvatica* (Araneae, Dysderidae): A valuable resource for functional and evolutionary genomic studies in chelicerates. GigaScience. 2019; **8**(8): giz099.

15 Sheffer MM, Hoppe A, Krehenwinkel H, et al. Chromosome-level reference genome of the European wasp spider *Argiope bruennichi*: a resource for studies on range expansion and evolutionary adaptation. Gigascience. 2021; **10**(1): giaa148.

16 Harvey MS, Austin AD, Adams M. The systematics and biology of the spider genus Nephila (Araneae: Nephilidae) in the Australasian region. Invertebrate Systematics. 2007; **21**(5): 407–451.

17 Hawes, TC. A spider that decorates its web perpendicular to the web plane. Tropical zoology. 2019; **32**(4): 202–211.

18 Kuntner M, Hamilton CA, Cheng RC, et al. Golden Orbweavers Ignore Biological Rules: Phylogenomic and Comparative Analyses Unravel a Complex Evolution of Sexual Size Dimorphism. Syst Biol. 2019; **68**(4): 555–572.

19 Eggs B, Sander D. Herbivory in Spiders: The Importance of Pollen for OrbWeavers. Plos One. 2013; **8**(11): e82637.

20 Reed JR, Backes WL. Formation of P450-P450 complexes and their effect on P450 function. Pharmacology & Therapeutics. 2012; **133**(3): 299–310.

21 Tsubota T, Shiotsuki T. Genomic and phylogenetic analysis of insect carboxyl/cholinesterase genes. Journal of Pesticide Science. 2010; **35**(3): 310–314.

22 Sheehan D, Meade G, Foley VM, et al. Structure, function and evolution of glutathione transferases: implications for classification of non-mammalian members of an ancient enzyme superfamily. Biochemical Journal. 2001; 360: 1–16.

23 Dermauwa W and Leeuwen TV. The ABC gene family in arthropods: Comparative genomics and role in insecticide transport and resistance. Insect Biochemistry and Molecular Biology. 2014; 45: 89–110.

24 Rane RV, Walsh TK, Pearce SL, et al. Are feeding preferences and insecticide resistance associated with the size of detoxifying enzyme families in insect herbivores? Current Opinion in Insect Science. 2016; 13: 70–76.

25 Oakeshott JG, Johnson RM, Berenbaum MR, et al. Metabolic enzymes associated with xenobiotic and chemosensory responses in *Nasonia vitripennis*. 2010; 19: 147–163.

26 Claudianos C, Ranson H, Johnson RM, et al. A deficit of detoxification enzymes: pesticide sensitivity and environmental response in the honeybee. Insect Molecular Biology. 2006; **15**(5):615–636.

27 Bushnell, B. BBtools. 2014. Retrieved from https://sourceforge.net/projects/bbmap/.

28 Vurture GW, Sedlazeck FJ, Nattestad M, et al. GenomeScope: fast reference-free genome

profling from short reads. Bioinformatics, 2017; **33**(14): 2202–2204.

29 Kolmogorov M, Yuan J, Lin Y, et al. Assembly of Long Error-Prone Reads Using Repeat Graphs. Nature Biotechnology. 2018; **37**(5): 540.

30 Roach MJ, Schmidt SA, Borneman AR. Purge Haplotigs: allelic contig reassignment for third-gen diploid genome assemblies. BMC Bioinformatics. 2018; **19**(1): 460.

31 Hu J, Fan J, Sun Z, et al. NextPolish: a fast and efficient genome polishing tool for long-read assembly. Bioinformatics. 2020; **36**(7): 2253–2255.

32 Li H. Minimap2: pairwise alignment for nucleotide sequences. Bioinformatics. 2018; **34**(18): 3094–3100.

33 Durand NC, Shamim MS, Machol I, et al. Juicer provides a one–click system for analyzing loop-resolution Hi-C experiments. Cell Systems. 2016; **3**(1): P95–98.

34 Ghurye J, Pop M, Koren S, et al. Scaffolding of long read assemblies using long range contact information. BMC genomics. 2017; 18: 527.

35 Zhang X, Zhang S, Zhao Q, et al. Assembly of allele-aware, chromosomal-scale autopolyploid genomes based on Hi-C data. Nat Plants. 2019; **5**(8): 833–845.

36 Chen Y, Ye W, Zhang Y, et al. High speed BLASTN: an accelerated MegaBLAST search tool. Nucleic Acids Research. 2015; **43**(16): 7762–7768.

37 Camacho C, George C, Vahram A, et al. 2009. BLAST+: architecture and applications. BMC Bioinformatics. 2009; 10: 421.

38 Waterhouse RM, Seppey M, Simao FA, et al. BUSCO applications from quality assessments to gene prediction and phylogenomics. Molecular Biology and Evolution. 2018; **35**(3): 543–548.

39 Flynn PM, Hubley P, Goubert P, et al. RepeatModeler2 for automated genomic discovery of transposable element families. PNAS. 2020; **117**(17): 9451–9457.

40 Bao W, Kojima KK and Kohany O. Repbase Update, a database of repetitive elements in eukaryotic genomes. Mobile DNA. 2015; 6: 11.

41 Smit AFA, Hubley R, Green P. RepeatMasker Open-4.0. 2013–2015. Retrieved from http://www.repeatmasker.org.

42 Nawrocki EP and Eddy SR. Infernal 1.1: 100-fold faster RNA homology searches. Bioinformatics. 2013; **29**(22): 2933–2935.

43 Chan PP and Lowe TM. tRNAscan-SE: Searching for tRNA Genes in Genomic Sequences. Methods in Molecular Biology. 2019; 1962: 1–14.

44 Holt C and Yandell M. MAKER2: an annotation pipeline and genome-database management tool for second-generation genome projects. BMC Bioinformatics. 2011; 12: 491.

45 Stanke M, Steinkamp R, Waack S, et al. AUGUSTUS: a web server for gene finding in eukaryotes. Nucleic Acids Research. 2004; 32: W309–W312.

46 Brůna T, Lomsadze A and Borodovsky M. GeneMark-EP+: eukaryotic gene prediction with self-training in the space of genes and proteins. NAR Genom Bioinform. 2020; **2**(2): lqaa026.

47 Hoff KJ, Lange S, Lomsadze A, et al. BRAKER1: Unsupervised RNA-Seq-Based Genome Annotation with GeneMark-ET and AUGUSTUS. Bioinformatics. 2016; 32: 767–769.

48 Kim D, Landmead B, Salzberg SL. HISAT: a fast spliced aligner with low memory

requirements. Nature Methods. 2015; **12**(4): 357–U121.

49 Kovaka S, Zimin AV, Pertea GM, et al. Transcriptome assembly from long-read RNA-seq alignments with StringTie2. Genome Biology. 2019; **20**(1):278.

50 Buchfink B, Xie C, and Huson DH. Fast and sensitive protein alignment using DIAMOND. Nature Methods. 2015; **12**(1):59–60.

51 Finn RD, Attwood TK., Babbitt PC, et al. InterPro in 2017-beyond protein family and domain annotations. Nucleic Acids Research. 2017; 45: D190–D199.

52 El-Gebali S, Mistry J, Bateman A, et al. The Pfam protein families database in 2019. Nucleic Acids Research. 2019; 47: D427–D432.

53 Mi HY and Thomas P. PANTHER Pathway: an ontology-based pathway database coupled with data analysis tools. Methods Mol Biology. 2009; 563: 123–140.

54 Lewis TE, Sillitoe I, Dawson N, et al. Gene3D: extensive prediction of globular domains in proteins. Nucleic Acids Research. 2018; 46: D435–D439.

55 Wilson D, Pethica R, Zhou Y, et al. SUPERFAMILY—sophisticated comparative genomics, data mining, visualization and phylogeny. Nucleic Acids Research. 2009; 37: D380–D386.

56 Marchler-Bauer A, Bo Y, Han L, et al. CDD/SPARCLE: functional classification of proteins via subfamily domain architectures. Nucleic Acids Research. 2017; 45: D200–D203.

57 Huerta-Cepas J, Forslund K, Coelho PL, et al. Fast genome-wide functional annotation through orthology assignment by eggNOG-mapper. Molecular Biology and Evolution. 2017; **34**(8): 2115–2122.

58 Huerta-Cepas J, Szklarczyk D, Heller D, et al. eggNOG 5.0: a hierarchical, functionally and phylogenetically annotated orthology resource based on 5090 organisms and 2502 viruses. Nucleic Acids Research. 2019; 47: D309–D314.

59 Emms DM, Kelly S. OrthoFinder: phylogenetic orthology inference for comparative genomics. Genome Biology. 2019; **20**(1): 238.

60 Katoh K, and Standley DM. MAFFT multiple sequence alignment software version 7: Improvements in performance and usability. Molecular Biology and Evolution. 2013; 30: 772–780.

61 Capella Gutierrez S, Silla Martinez JM, Gabaldon T. TrimAl: a tool for automated alignment trimming in large-scale phylogenetic analyses. Bioinformatics. 2009; **25**(15): 1972–1973.

62 Kück P, and Longo GC. FASconCAT-G: extensive functions for multiple sequence alignment preparations concerning phylogenetic studies. Frontiers in Zoology. 2014; **11**(1):81.

63 Minh BQ, Schmidt HA, Chernomor O. IQ-TREE 2: New Models and Efficient Methods for Phylogenetic Inference in the Genomic Era. Molecular Biology and Evolution. 2020; **37**(5): 1530–1534.

64 Yang Z. PAML 4: phylogenetic analysis by maximum likelihood. Molecular Biology and Evolution. 2007; **24**(8): 1586–1591.

65 Waddington J, Rudkin DM, Dunlop JA. A new mid-Silurian aquatic scorpion-one step closer to land? Biology Letters. 2015; **11**(1): 20140815.

66 Wendruff AJ, Babcock LE, Wirkner CS, et al. A Silurian ancestral scorpion with fossilised

internal anatomy illustrating a pathway to arachnid terrestrialisation. Scientific Reports. 2020; **10**(1): 14.

67 Han MV, Thomas GW, and Lugo-Martinez J. Estimating gene gain and loss rates in the presence of error in genome assembly and annotation using CAFE 3. Molecular Biology and Evolution. 2013; **30**(8):1987–1997.

68 Chen CJ, Chen H, Zhang Y, et al. TBtools: An Integrative Toolkit Developed for Interactive Analyses of Big Biological Data. Molecular Plant. 2020; **13**(8): 1194–1202.

69 Steinegger M, Soding J. MMseqs2 enables sensitive protein sequence searching for the analysis of massive data sets. Nature biotechnology. 2017; **35**(11): 1026–1028.

70 Mulder N, Apweiler R. InterPro and InterProScan: tools for protein sequence classification and comparison. Methods Molecular Biology. 2007; 396: 59–70.

71 Werck-Reichhart D. and Feyereisen R. Cytochromes P450: a success story. Genome Biology. 2000; **1**(6): REVIEWS3003.

72 Liao Y, Smyth GK and Shi W. FeatureCounts: an efficient general-purpose program for assigning sequence reads to genomic features. Bioinformatics. 2014; **30**(7):923–930.

73 Wang YP, Tang HB, Jeremy DD, et al. MCScanX: a toolkit for detection and evolutionary analysis of gene synteny and collinearity. Nucleic Acids Resarch. 2012; **40**(7): e49.

74 Pace RM, Grbic M, and Nagy LM. Composition and genomic organization of arthropod Hox clusters. Evodevo. 2016; 7: 11.

75 Zhong YF, Holland PW. HomeoDB2: functional expansion of a comparative homeobox gene database for evolutionary developmental biology. Evolution & Development. 2011; **13**(6): 567–568.

76 Wybouw N, Zhurov V, Martel C, et al. Adaptation of a polyphagous herbivore to a novel host plant extensively shapes the transcriptome of herbivore and host. Molecular Ecology. 2015; **24**(18):4647–4663

77 Dermauw W, Wybouw N, Rombauts S, et al. A link between host plant adaptation and pesticide resistance in the polyphagous spider mite *Tetranychus urticae*. PNAS. 2013; **110**(2): E113–E122.

78 Grbi´c M, Van LT, Clark RM, et al. 2011. The genome of *Tetranychus urticae* reveals herbivorous pest adaptations. Nature. 2011; **479**(7374): 487–492.

79 Van Leeuwen T, Dermauw W. The Molecular Evolution of Xenobiotic Metabolism and Resistance in Chelicerate Mites. Annual Review of Entomology. 2016; 61: 475–498.

80 Gui FR, Lan TM, Zhao Y, et al. Genomic and transcriptomic analysis unveils population evolution and development of pesticide resistance in fall armyworm *Spodoptera frugiperda*. Protein & Cell. 2020; DOI: 10.1007/s13238-020-00795-7.

81 Pym A, Singh KS, Nordgren A, et al. Host plant adaptation in the polyphagous whitefly, *Trialeurodes vaporariorum*, is associated with transcriptional plasticity and altered sensitivity to insecticides. BMC Genomics. 2019; **20**(1): 996.

82 Cheng TC, Wu JQ, Wu Y, et al. Genomic adaptation to polyphagy and insecticides in a major East Asian noctuid pest. 2017; **1**(11): 1747–1756.

83 Pearce SL, Clarke DF, East PD, et al. Genomic innovations, transcriptional plasticity and gene loss underlying the evolution and divergence of two highly polyphagous and invasive Helicoverpa pest species. 2017; 15: 63.

84 Tsubota T, Shiotsuki T. Genomic analysis of carboxyl/cholinesterase genes in the

silkworm *Bombyx mori*. BMC Genomics. 2010; 11: 377.

85 Lee SH, Kang JS, Min JS, et al. Decreased detoxification genes and genome size make the human body louse an efficient model to study xenobiotic metabolism. Insect Molecular Biology. 2010; **19**(5): 599–615.

86 Feyereisen R. Evolution of insect P450. Biochemical Society Transactions. 2006; **34**(6): 1252–1255.

87 Lv B, Wang J, Zhuo JZ, Yang HL, Yang SF, Wang Z, Song QS. Transcriptome sequencing reveals the effects of cadmium toxicity on the cold tolerance of the wolf spider Pirata subpiraticus. Chemosphere. 2020; 254: 126802.

88 Fang SM. Insect glutathione S-transferase: a review of comparative genomic studies and response to xenobiotics. Bulletin of Insectology. 2012; **65**(2): 265–271.

89 Pavlidi N, Tseliou V, Riga M, et al. Functional characterization of glutathione S-transferases associated with insecticide resistance in *Tetranychus urticae*. Pestic Biochem Physiol. 2015; 121: 53–60.

90 Leite DJ, Ninova M, Hilbrant M, et al. Pervasive microRNA Duplication in Chelicerates: Insights from the Embryonic microRNA Repertoire of the Spider *Parasteatoda tepidariorum*. Genome Biology and Evolution. 2016; **8**(7): 2133–2144.

91 Leite DJ, Baudouin-Gonzalez L, Iwasaki-Yokozawa S, et al. Homeobox gene duplication and divergence in arachnids. Molecular Biology and Evolution. 2018; **35**(9): 2240–2253.

92 Nolan ED, Santibáñez-López CE, Sharma PP. Developmental gene expression as a phylogenetic data class: support for the monophyly of Arachnopulmonata. Development Genes and Evolution. 2020; **230**(2): 137–153.

93 Shingate P, Ravi V, Prasad A, et al. Chromosome-level assembly of the horseshoe crab genome provides insights into its genome evolution. Nature Communications.2020; **11**(1): 2322.

94 Kenny NJ, Chan KW, Nong W, et al. Ancestral whole-genome duplication in the marine chelicerate horseshoe crabs. Heredity. 2016. **116**(2): 190–199.

95 Aury JM, Jaillon O, Duret L, et al. Global trends of whole-genome duplications revealed by the ciliate *Paramecium tetraurelia*. Nature. 2006; **444**(7116): 171–178.

96 Fan Z, Yuan T, Liu P, Wang LY, Jin JF et al. A chromosome- level genome of the spider *Trichonephila antipodiana*. *GigaScience* Database 2021. http://dx.doi.org/10.5524/100868

Table 1. Comparison of the quality of the *Trichonephila antipodiana* genome with that of other published spider genomes

Species	Genome size (Gb)	Scaffold N50 (kbp)	Contig N50 (kbp)	Accession number
Stegodyphus dumicola	2.55	254.13	254.13	GCA_010614865.1
Anelosimus studiosus	2.03	4.79	1.13	GCA_008297655.1
Pardosa pseudoannulata	4.21	711.40	23.23	GCA_008065355.1
Latrodectus hesperus	1.23	39.47	15.96	GCA_000697925.2
Dysdera silvatica	1.36	38.02	25.71	GCA_006491805.1
Loxosceles reclusa	3.26	63.24	1.83	GCA_001188405.1
Trichonephila clavipes	2.44	62.96	7.99	GCA_002102615.1
Parasteatoda tepidariorum	1.45	4,055.36	10.15	GCA_000365465.3
Stegodyphus mimosarum	2.74	480.64	40.15	GCA_000611955.2
Araneus ventricosus	3.65	59.62	-	BGPR01000001-BGPR01300721 (DDBJ)
Argiope bruennichi	1.67	124,236.00	288.40	unpublicized
Trichonephila antipodiana	2.29	172,892.00	1,138.00	-

Table 2. Statistics of the DNA sequence data used for genome assembly

Pair-end libraries	Clean data (Gb)	Sequencing coverage (×)	Insert sizes
Illumina reads	305.96	133	300 bp
PacBio reads	235.79	103	20 Kb
Hi-C	215.05	94	300 bp
RNA	10.27	-	300 bp
Total	767.07	-	-

Table 3. Summary of each step in construction of the *Trichonephila antipodiana* genome assembly

Assembly	Total length (Gb)	No. scaffolds (chromosome)	N50 length (Mb)	Longest scaffold (MB)	GC (%)	BUSCO (n = 1066) (%)			
						C	D	F	M

Flye	2.38	16,680	1.21	11.071	31.8	95.2	5.2	0.9	3.9
Purge Dups	2.31	10,670	1.26	11.071	31.8	95.3	4.0	0.8	3.9
Pilon	2.31	10,670	1.26	11.082	31.7	95.3	4.3	0.7	4.0
Hi-C	2.29	377(13)	137.66	230.27	31.7	94.8	4.1	1.0	4.2
Final genome assembly	2.29	377(13)	137.66	230.17	31.7	94.8	4.1	1.0	4.2
Transcript assembly	69.29 Mb	30,586	3.43 Kb	43.99 Kb	34.3	97.2	33.4	1.1	1.7

Table 4. Statistics of the repetitive sequences identified in *Trichonephila antipodiana*

Type	Number	Length (bp)	% of genome
**SINEs:**	**106,507**	**25,417,898**	**1.11**
tRNA-Deu	44,262	10,710,146	0.47
MIR	28,198	6,417,754	0.28
tRNA-Core	19,575	5,140,899	0.22
tRNA	3,964	507,398	0.02
**LINEs**	**197,390**	**83,281,087**	**3.63**
Penelope	49,982	30,623,444	1.33
I	56,156	19,510,142	0.85
I-Jockey	27,196	13,846,368	0.60
R1	14,033	5,652,471	0.25
**LTR elements**	**101,690**	**79,698,444**	**3.47**
Gypsy	53,122	53,035,139	2.31
Pao	26,368	19,084,383	0.83
Copia	15,295	6,965,923	0.30
ERV1	4,052	178,070	0.01
**DNA elements**	**1,393,742**	**518,114,026**	**22.58**
TcMar-Tc1	332,152	164,809,170	7.18
hAT-Charlie	399,282	142,099,848	6.19
TcMar-Mariner	89,418	39,370,728	1.72
Kolobok-Hydra	37,030	30,581,663	1.33
**Unclassified**	**1,961,792**	**508,599,211**	**22.17**
**Total interspersed repeats**		**1,215,110,666**	**52.96**
**Small RNA**	**72,066**	**16,577,914**	**0.72**
**Satellites**	**7,513**	**2,910,802**	**0.13**
**Simple repeats**	**450,644**	**24,805,223**	**1.08**
**Low complexity**	**84,430**	**4,336,839**	**0.19**

Table 5 counts of protein associated with Detoxification Enzymes in *Trichonephila antipodiana* and other Arthropods

species	Type	P450s	ABCs	CCEs	GSTs	reference
Trichonephila antipodiana	polyphagous	167	48	48	22	–
Spodoptera frugiperda	polyphagous	425	58	–	29	[80]
Tribolium castaneum	polyphagous	128	73	60	35	[81]
Spodoptera litura	polyphagous	138	54	–	47	[82]
Helicoverpa armigera	polyphagous	114	54	97	42	[83]
Tetranycbus urticae	polyphagous	81	103	71	31	[77,78]
Trialeurodes vaporariorum	polyphagous	80	46	31	26	[81]
Manduca sexta	oligophagous	103	54	96	31	[83]
Bombyx mori	monophagous	83	51	69	26	[84,77]
Pediculus humanus humanus	monophagous	37	40	–	13	[85]

Note: "–" means lack of data or no reference

Figure 1

Click here to access/download;Figure;Figure1.jpg

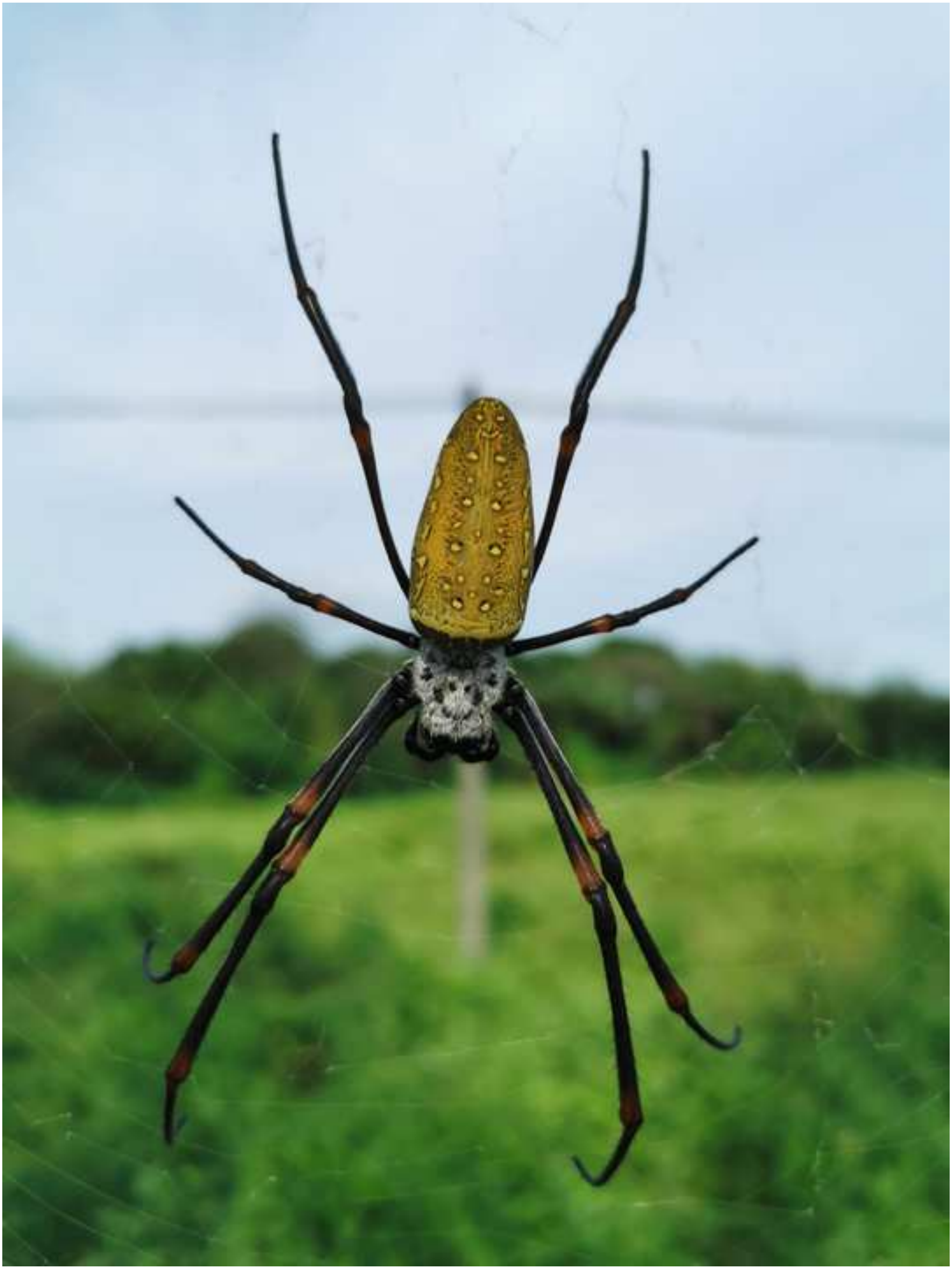

Figure 2

Click here to access/download;Figure;Figure2.pdf

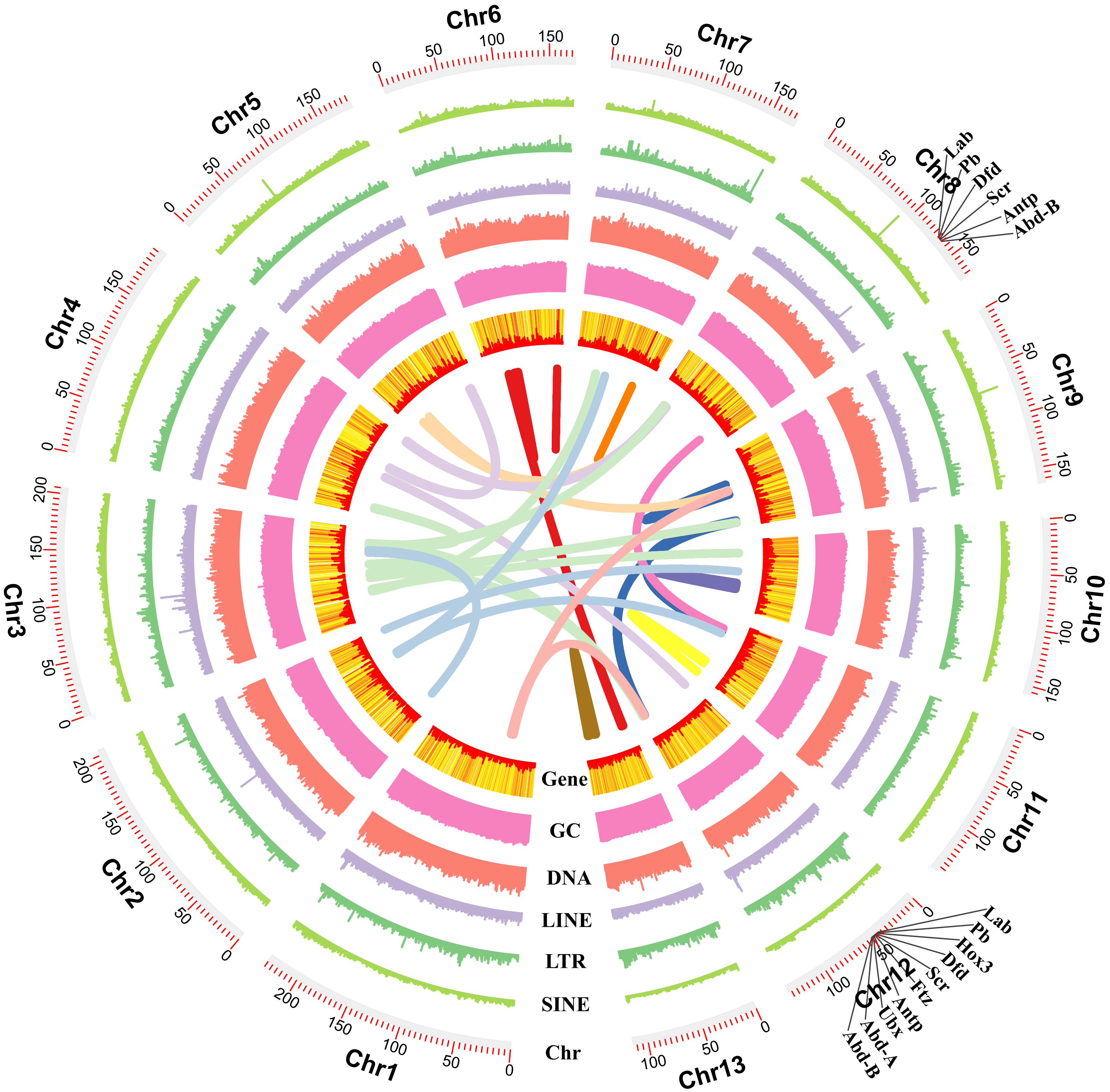

Click here to access/download;Figure;Figure3.pdf

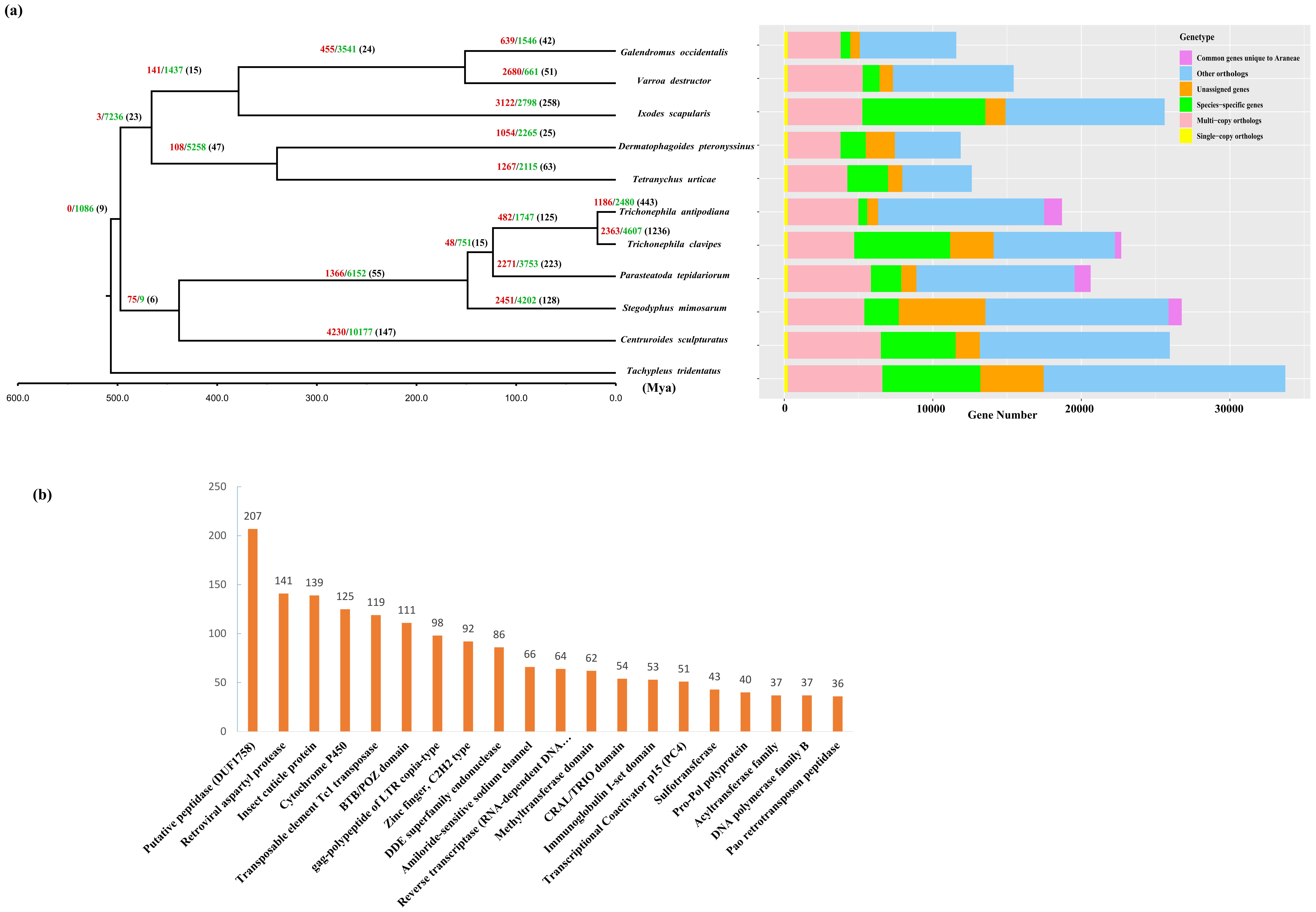

Figure 4

Click here to access/download;Figure;Figure4.pdf

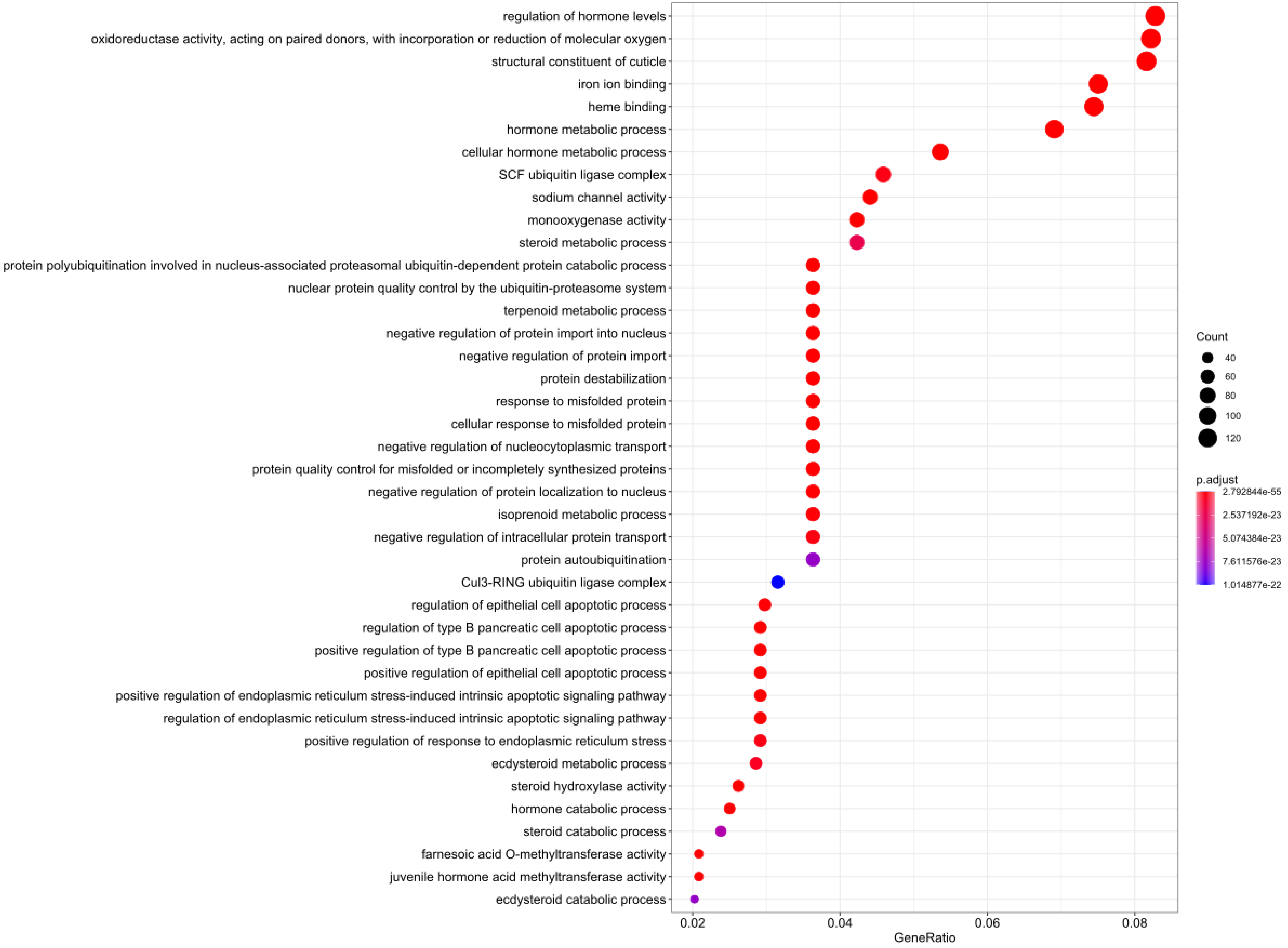

Figure 5

Click here to access/download;Figure;Figure5.pdf

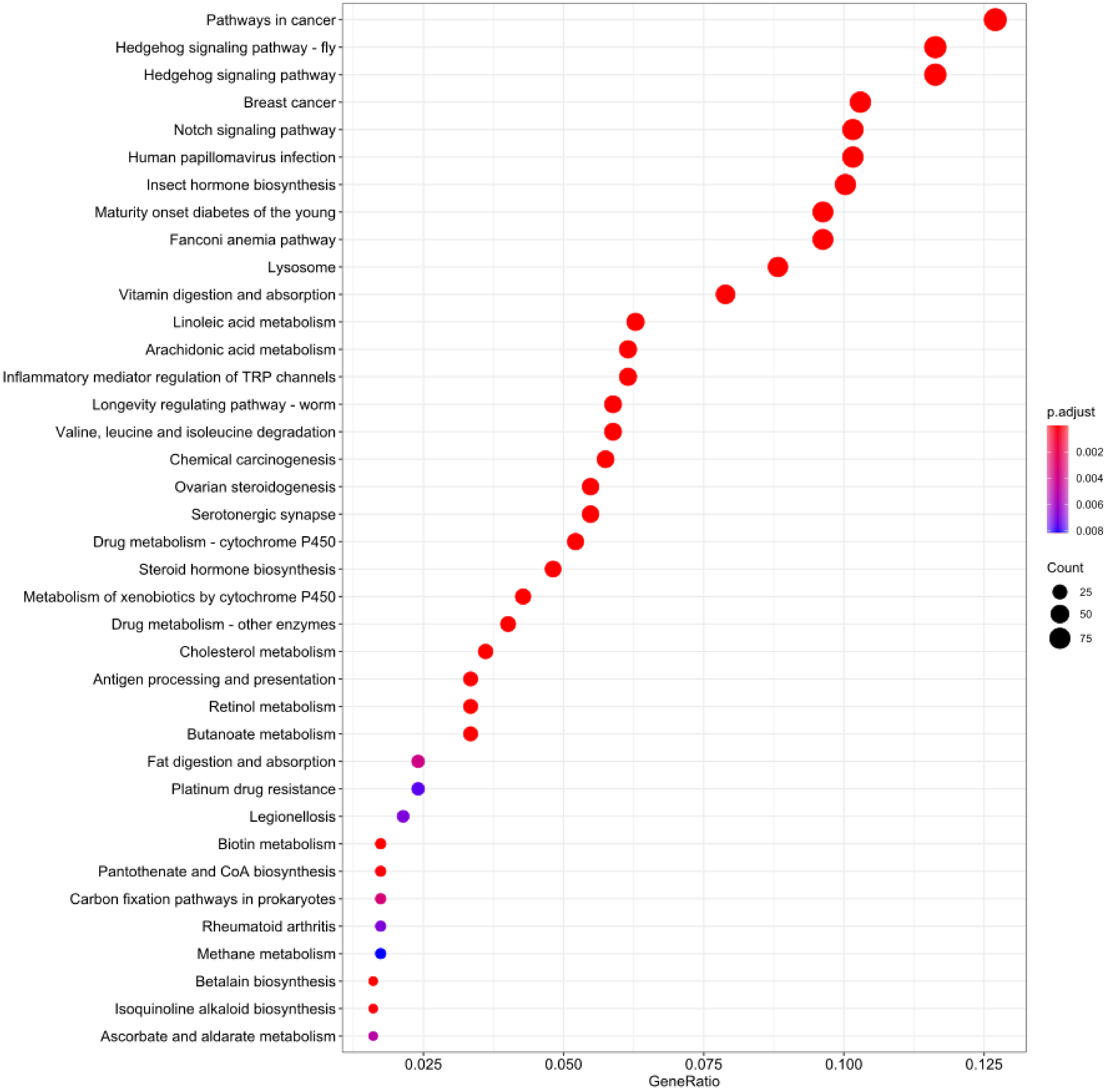

Figure 6

Click here to access/download;Figure;Figure6.pdf

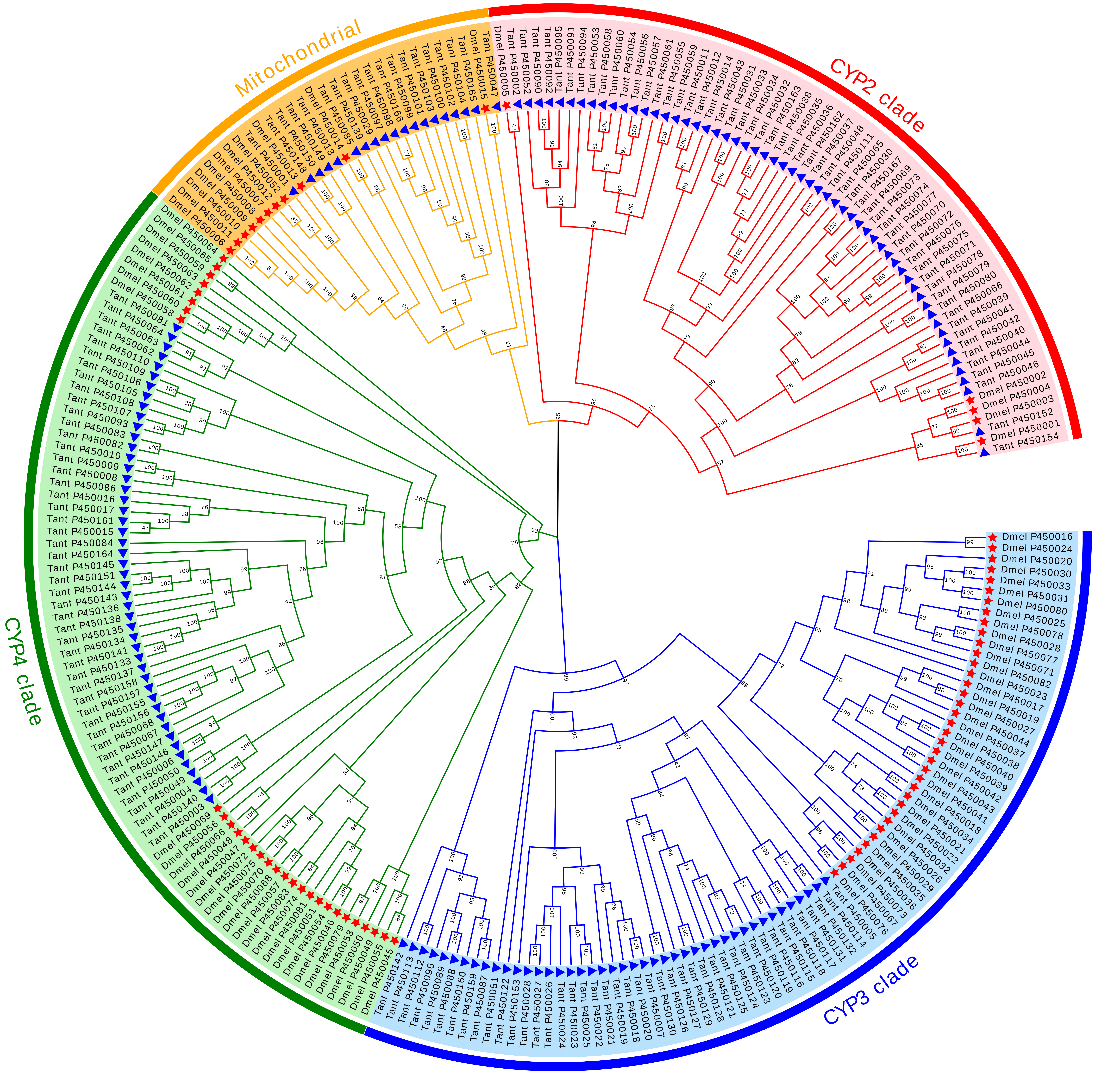

Figure 7

Click here to access/download;Figure;Figure7.pdf

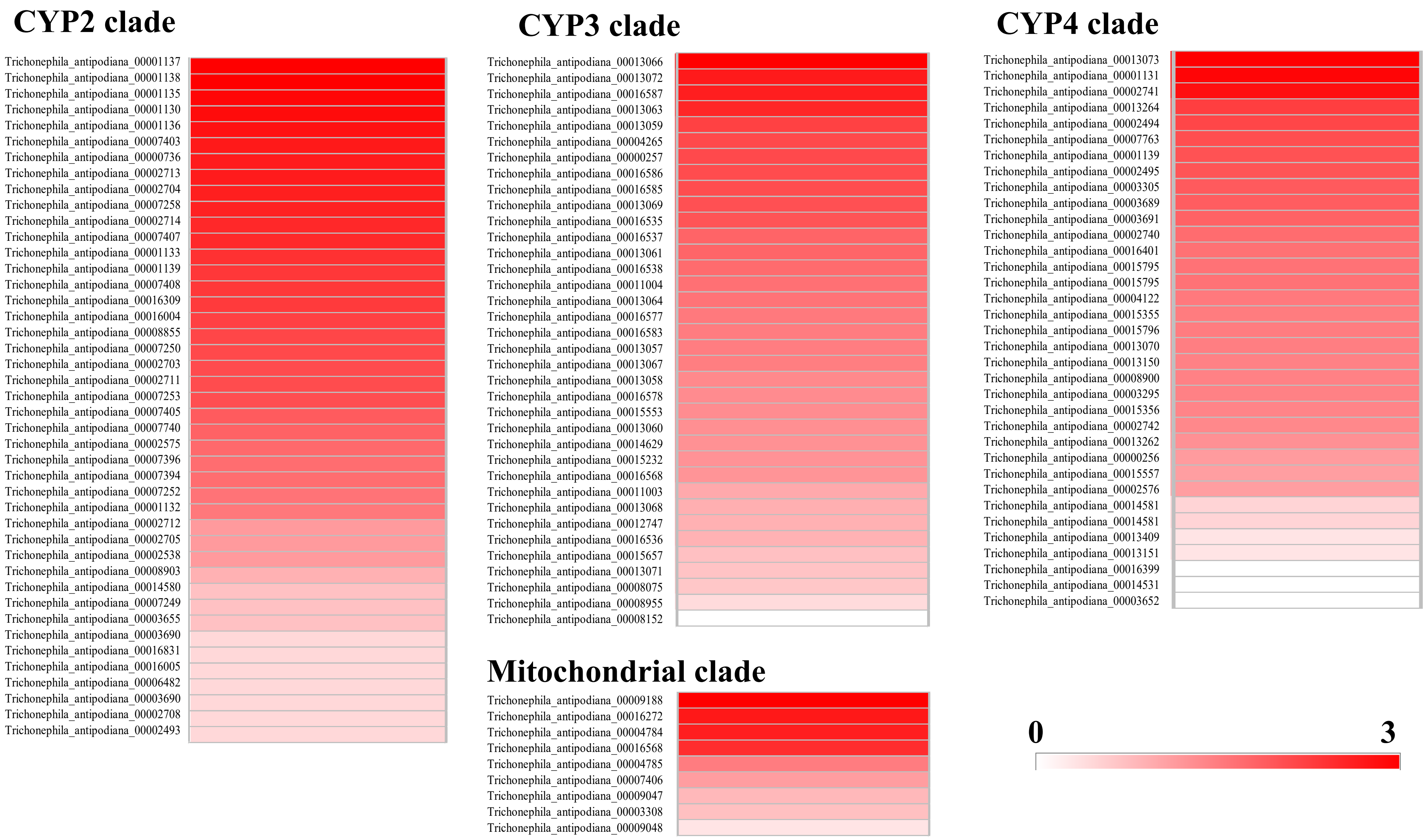

FigureS1

Click here to access/download;Figure;FigureS1.pdf

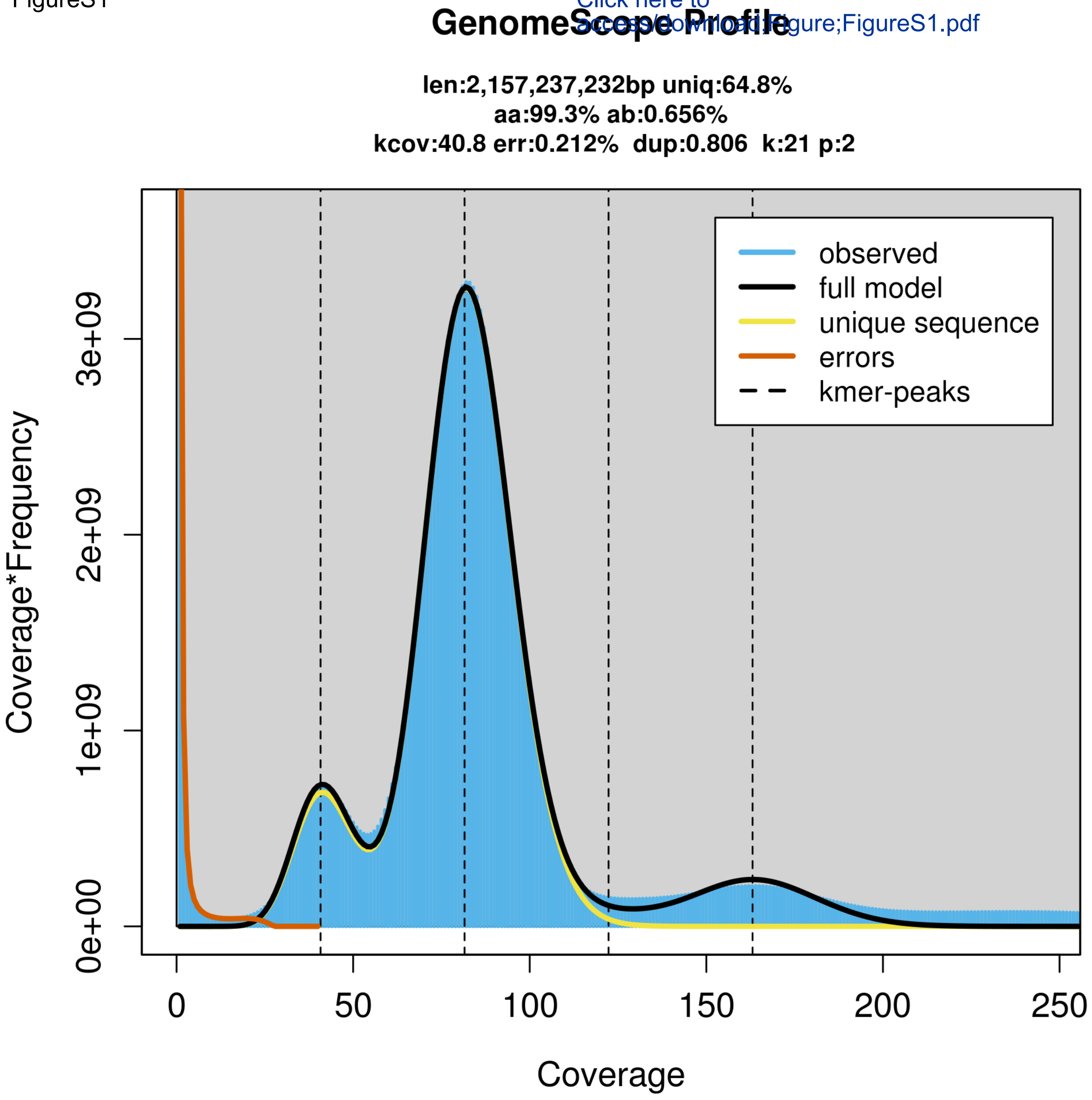

FigureS2
Click here to access/download;Figure;FigureS2.pdf

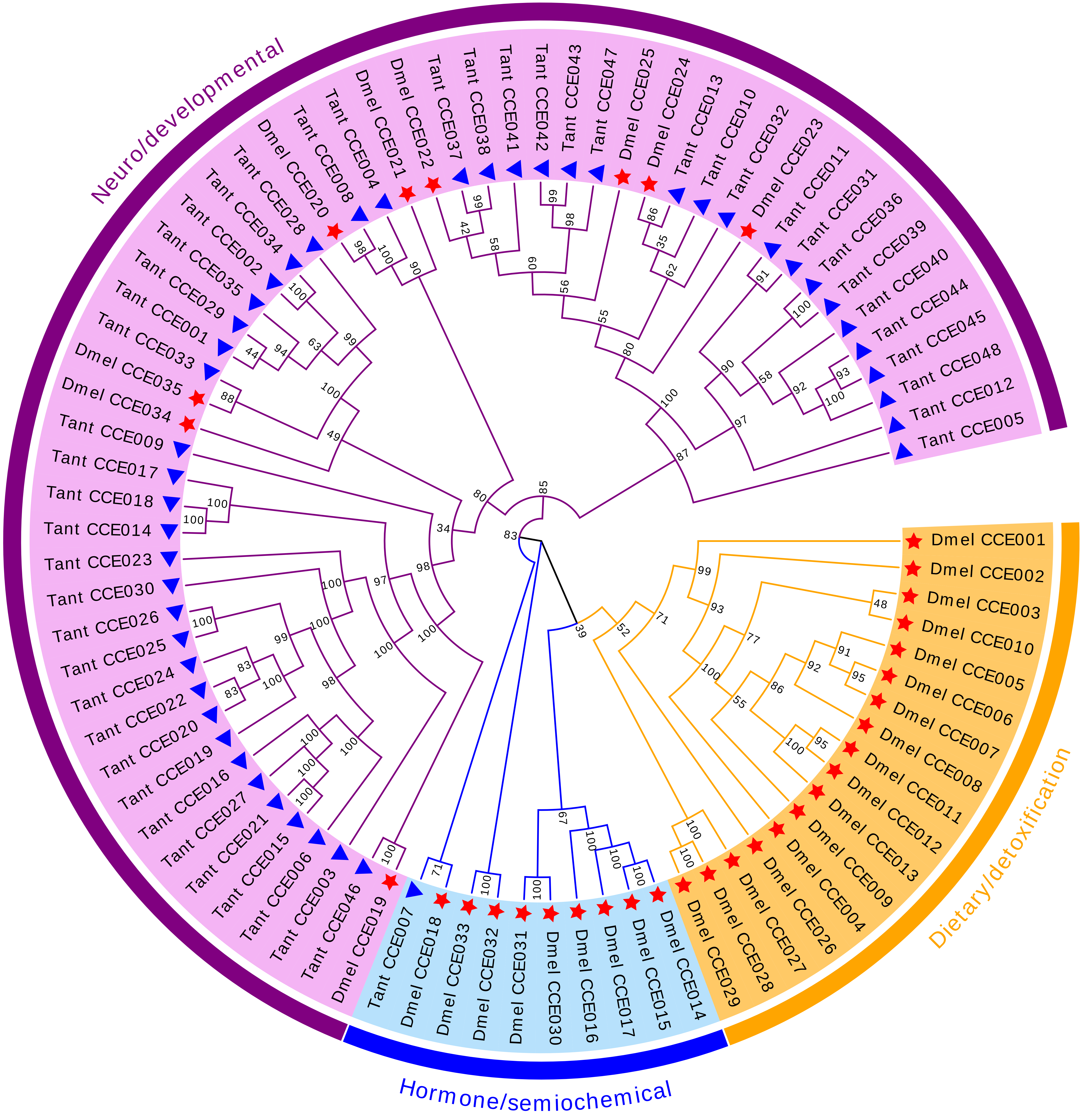

Click here to access/download;Figure;FigureS3.pdf

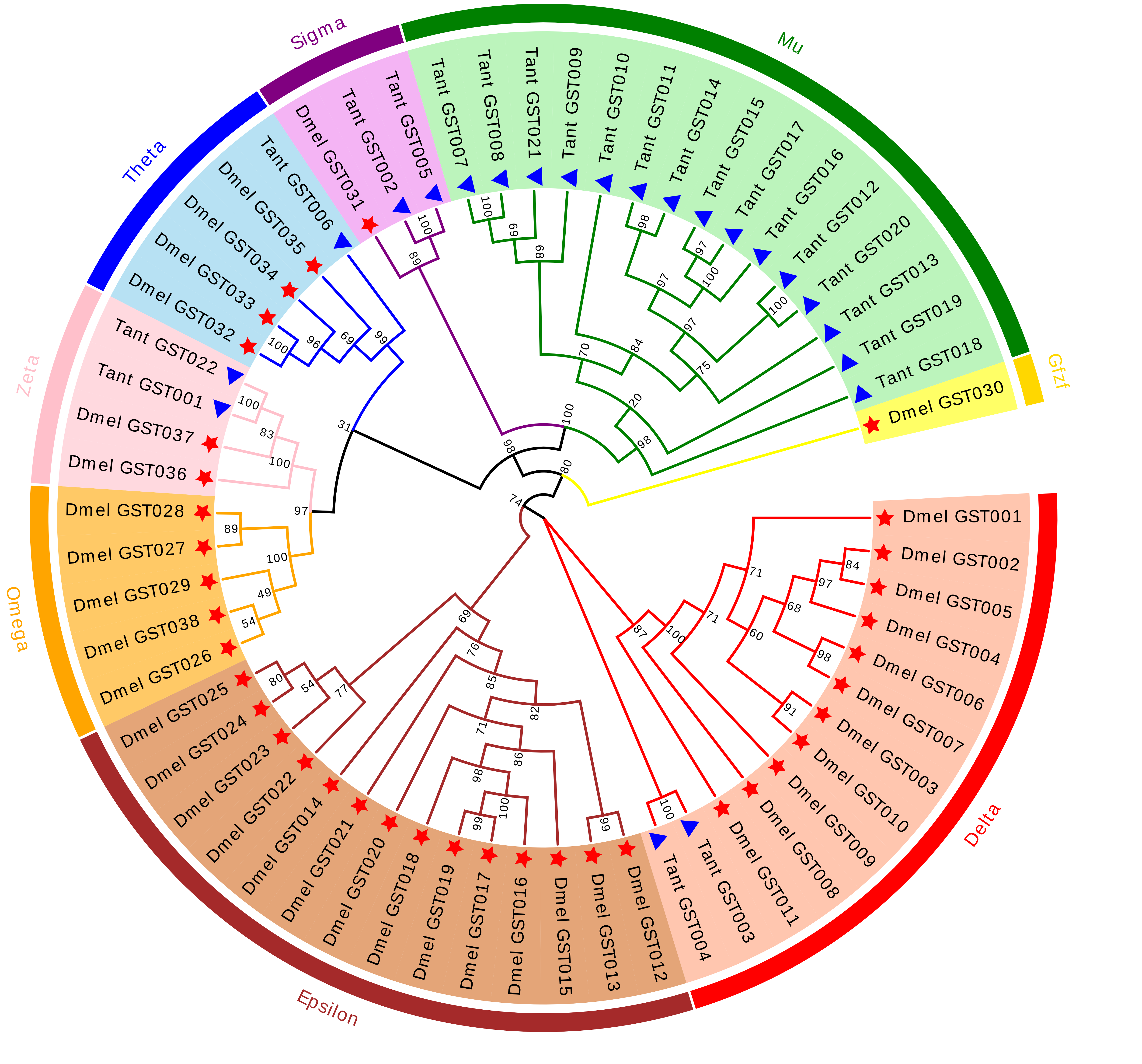

Click here to access/download;Figure;FigureS4.pdf

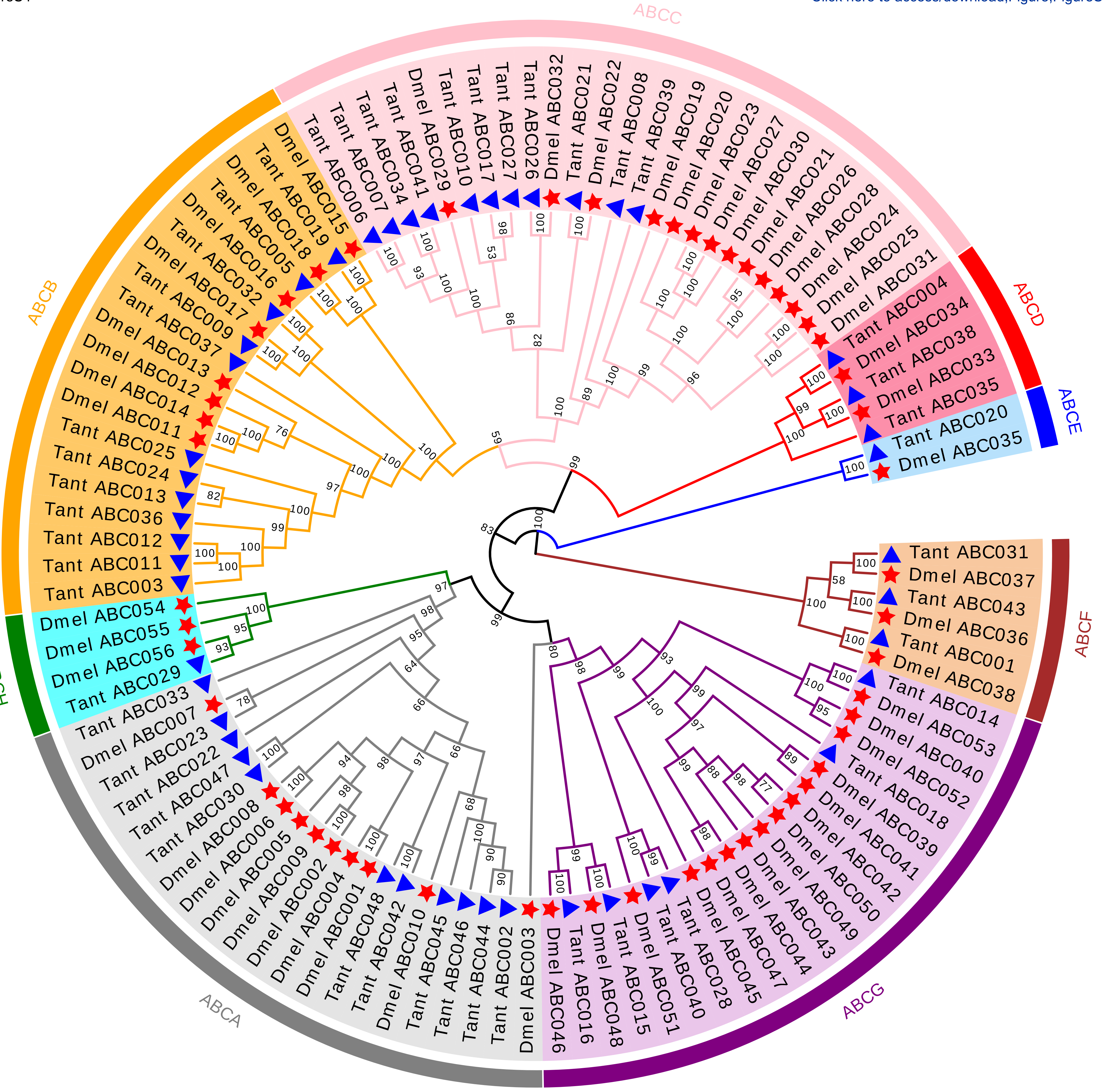

GST proteins of Trichonephila antipodiana

Click here to access/download
Supplementary Material
GST.Tan.fa

ABC proteins of Trichonephila antipodiana

Click here to access/download
Supplementary Material
ABC.Tant.fa

CCE proteins of Trichonephila antipodiana

Click here to access/download
Supplementary Material
CCE.Tan.fa

P450 proteins of Trichonephila antipodiana

Click here to access/download
Supplementary Material
P450.Tant.fa
