# Supplementary material for: A chromosome‐level genome of the spider Trichonephila antipodiana reveals the genetic basis of its polyphagy and evidence of an ancient whole-genome duplication event
Source: Gigascience. 2021 Mar 19;10(3):giab016. doi: 10.1093/gigascience/giab016 (PMC7976613; doi:10.1093/gigascience/giab016)
Supplement: giab016_Response_to_Reviewer_Comments_Original_Submission [file giab016_response_to_reviewer_comments_original_submission.pdf]

Author's Response To Reviewer Comments

Close

Response to editor:
In addition, please also include a picture of the sequenced spider species as a figure in the paper, if available.

Response:
Thanks for your suggestion. We added it, shown in Figure1.

Response to reviewer#1:
1. Molecular dating: The molecular dating should be redone with a more recent calibration because the PaleoDB database is outdated for Chelicerata. The split between scorpions and spiders should be assigned a minimum age of 435-439 Mya, based on the recently described fossils Eramoscorpius brucensis (Waddington et al. 2015) and Parioscorpio venator (Wendruff et al. 2020). The current split between these two groups in the dated tree has an implausibly young floor (423.79 Mya). This change will have little effect on the overall manuscript.

Response:
Thanks for your suggestions. We redone the divergence analysis with split between scorpions and spiders assigned a minimum age of 435-439 Mya and changed my manuscript.

2. Lines 198 and 361: What is the source of these 236 genes? How were they inferred to be single copy and why were these specific loci chosen?

Response:
The source of these 236 genes were obtained from the results of orthologous gene clusters analysis by the soft of OrthoFinder. Using OrthoFinder, we obtained a total of 203,348 genes among the 11 species, which were clustered into 20,785 orthogroups. The single-copy orthologs was identified when every species only included one gene. These specific loci were chosen because the single copy genes have one physical location in the genome and can have orthologs in different species.

3. The authors should avoid the use of Ks distributions to test this whole genome duplication. It is well known that this method is highly sensitive and fails for ancient (pre-Cretaceous) WGD events due to saturation. Given that this duplication must be older than 439 Myr, there is no chance that Ks distance distributions are going to be dispositive of a WGD event.

Response:
Accept. We deleted the distribution analysis of pairwise synonymous substitution rates (Ks).

4. In two places in the manuscript, the hypothesis of the arachnopulmonate WGD is referred to as an "assumption". This term is inappropriate in this context, given the large swath of evidence supporting this hypothesis. I would recommend the authors to refer to this idea as a "hypothesis" or an "inference", as well as include references to the following works:
a. Leite DJ, Ninova M, Hilbrant M, et al. 2016 Pervasive microRNA Duplication in Chelicerates: Insights from the Embryonic microRNA Repertoire of the Spider Parasteatoda tepidariorum. Genome Biol. Evol 8:2133-2144. (doi: 10.1093/gbe/evw143)
b. Leite DJ, et al. (2018) Homeobox gene duplication and divergence in arachnids. Mol. Biol. Evol. 35:2240-2253. (doi: 10.1093/molbev/msy125)
c. Nolan ED, Santibáñez-López CE, Sharma PP. 2020 Developmental gene expression as a phylogenetic data class: support for the monophyly of Arachnopulmonata. Dev. Genes Evol. 230:137-153. (doi: 10.1007/s00427-019-00644-6)

Response:
Accept. We changed and added the references (90, 91, 92).

5. It is not explained in the methods how synteny analysis was performed. Please explain the pipeline or the software used to make these inferences.

Response:
The synteny analysis pipeline was shown in the methods of WGD analyses (line 278-283 and 292-293).

Minor comments:

1. Lines 40-41: "Spiders (Araneae) have a worldwide distribution, have conquered virtually all ecological environments". Please change this to read "virtually all terrestrial environments".

Response:
Accept. We changed it.

2. Line 62: "PacBio"

Response:
Accept. We changed it.

3. Line 254: "It has been reported that an ancient WGD event occurred in the spider lineage". Please replace this with "It has been reported that an ancient WGD event occurred in the common ancestor of spiders and scorpions".

Response:
Accept. We changed it.

4. Line 269: "In metazoans, the 10 highly conserved Hox genes play important roles…" The number of Hox genes is highly variable across Metazoa. It is inferred that the common ancestor of Panarthropoda bore 10 Hox genes, though this number varies across arthropod lineages as well. I suggest to change this to: "In arthropods, 10 highly conserved Hox genes that are inferred to occur in the common ancestor of Panarthropoda play important roles…"

Response:
Accept. We changed it.

5. Lines 459-460: On the WGD events in Xiphosura, please be sure to cite two recent papers (both Shingate et al. 2020) on the most recent and high-quality genomes of Carcinoscorpius and one of the Tachypleus species. There is additional evidence therein that the Xiphosura WGD events are independent of the Arachnopulmonata WGD event.

Response:
Accept. We cited the reference (88, 89).

Response to reviewer#2:
A chromosome-level genome of the spider Trichonephila antipodiana reveals the genetic basis of its polygaphy and evidence of an ancient whole-genome duplication event
The 'Background' section do not really build up to the idea of polyphagy and detoxification abilities. Consequently, I am, as not being an expert in this field, not convinced about the idea that this species should have high detoxification abilities due to polyphagy, and that the detoxification related gene families actually are detoxification related gene families. The 'Background' is mainly a patchwork of more or less specific information about spiders. Immune system, sexual dimorphism, how many spider genomes etc does not really provide much useful information for this study. It would be nice if the authors could collect information from other studies about food ranges (polyphagy), how that can be toxic, and what mechanisms/genes exist to overcome toxicity. If possible even couple it with genome duplication. That may enable them to come up with some hypotheses about what to find in this genome. If the authors could provide an analysis including a number of other arthropods, that could demonstrate a correlation between food niche width and evolution of the gene families assumed to be involved in detoxification, it would strengthen their main conclusion a lot.

The whole-genome duplication has been shown before, so I believe that making this a major point of this study (even putting it in the title) is too much. Fair enough to provide data that confirms previous

findings and extent the analyses with better data (chromosome level assembly).

Response:
Thank you for your suggestions. All of your suggestions have important guiding significance for us.
1）We changed the 'Background' and 'Conclusion' section.
2）We added some evidence about the association about polyphagy and detoxification. Such as the counts of P450 genes expressed in the whole body according to the RNA-data (Figure 7), the size of these gene families in some polyphagous, monophagous and oligophagous species (Table 5).
3）We reduced the part of whole-genome duplication analysis, and deleted the distribution analysis of pairwise synonymous substitution rates (Ks).

-line 43: production of silk - they cite 'Recent Advances in Development of Functional Spider Silk-Based Hybrid Materials'

Response:
Accept. We cite a new reference (2).

-line 45: whole genome duplication - they cite a paper (that is not yet peer-reviewed) that do not show the whole-genome duplication event, while a 2017 paper showed just that (https://bmcbiol.biomedcentral.com/articles/10.1186/s12915-017-0399-x?utm_campaign=BMC40864B&utm_medium=BMCemail&utm_source=Teradata)

Response:
Accept. We changed the reference (7).

Line 52: + Stegodyphus mimosarum and Acanthoscurria geniculate (https://onlinelibrary.wiley.com/doi/full/10.1111/jeb.12780)

Response:
Accept. We changed and added the reference (13).

Line 61-62: Also Stegodyphus dumicola (https://www.mdpi.com/2073-4425/10/2/137)

Response:
Accept. We changed and added the reference (11).

Line 101: what muscle tissue? From a leg?

Response:
Yes, the muscle tissue obtained from the spider’s leg.

Line 254: not in the spider lineage, but in the ancestor of spiders and scorpions.

Response:
Accept. We changed.

Line 302: I think Table 4 is wrong here.

Response:
Accept. We changed.

Line 317: I only see 4 tables in the manuscript.

Response:
Accept. We changed.

Line 331: numbers of exons and introns per gene seem wrong.

Response:
Accept. We changed (line 346).

Line 378: I am not sure the ref12 demonstrate a powerful spider immune response?

Response:
Accept. We changed and add the new reference (13).

Line 460: right references?

Response:
Thanks for your suggestion. We cite the reference with two recent papers on the most recent and high-quality genomes of Carcinoscorpius and one of the Tachypleus species (94, 95).

Line 464: ref should be 77.

Response:
Accept. We changed and cited the right reference.

Close
