# Supplementary material for: A chromosome‐level genome of the spider Trichonephila antipodiana reveals the genetic basis of its polyphagy and evidence of an ancient whole-genome duplication event
Source: Gigascience. 2021 Mar 19;10(3):giab016. doi: 10.1093/gigascience/giab016 (PMC7976613; doi:10.1093/gigascience/giab016)
Supplement: giab016_Reviewer_1_Report_Original_Submission — Prashant Sharma -- 12/7/2020 Reviewed [file giab016_reviewer_1_report_original_submission.pdf]

Reviewer Report

Title: A chromosomeâ€level genome of the spider Trichonephila antipodiana reveals the genetic basis of its polyphagy and evidence of an ancient whole-genome duplication event

Version: Original Submission **Date:** 12/7/2020

Reviewer name: Prashant Sharma

Reviewer Comments to Author:

Fan et al. (manuscript GIGA-D-20-00316) present a high-quality chromosome-level assembly for the Australasian species Trichonephila antipodiana. The authors have sequenced this spider's genome with high coverage and with an array of cutting-edge methods. The genome is highly complete, as inferred by comparison to the BUSCO-Ar gene set. The authors use this genome to explore lineage-specific phenomena (e.g., genome expansions in detoxification genes) and test the hypothesis of a genome duplication in Arachnopulmonata (the common ancestor of spiders and scorpions). The authors' results nicely confirm previous comparative genomic studies of chelicerate evolution.

The work is well written, clearly understandable, and mostly complete. I have minor suggestions for improvement and some requests for clarification.

1. Molecular dating: The molecular dating should be redone with a more recent calibration because the PaleoDB database is outdated for Chelicerata. The split between scorpions and spiders should be assigned a minimum age of 435-439 Mya, based on the recently described fossils Eramoscorpius brucensis (Waddington et al. 2015) and Parioscorpio venator (Wendruff et al. 2020). The current split between these two groups in the dated tree has an implausibly young floor (423.79 Mya). This change will have little effect on the overall manuscript.
2. Lines 198 and 361: What is the source of these 236 genes? How were they inferred to be single copy and why were these specific loci chosen?
3. The authors should avoid the use of Ks distributions to test this whole genome duplication. It is well known that this method is highly sensitive and fails for ancient (pre-Cretaceous) WGD events due to saturation. Given that this duplication must be older than 439 Myr, there is no chance that Ks distance distributions are going to be dispositive of a WGD event.
4. In two places in the manuscript, the hypothesis of the arachnopulmonate WGD is referred to as an "assumption". This term is inappropriate in this context, given the large swath of evidence supporting this hypothesis. I would recommend the authors to refer to this idea as a "hypothesis" or an "inference", as well as include references to the following works:

a. Leite DJ, Ninova M, Hilbrant M, et al. 2016 Pervasive microRNA Duplication in Chelicerates: Insights from the Embryonic microRNA Repertoire of the Spider Parasteatoda tepidariorum. Genome Biol. Evol 8:2133-2144. (doi: 10.1093/gbe/evw143)

b. Leite DJ, et al. (2018) Homeobox gene duplication and divergence in arachnids. Mol. Biol. Evol. 35:2240-2253. (doi: 10.1093/molbev/msy125)

c. Nolan ED, SantibÃ¡Ã±ez-LÃ³pez CE, Sharma PP. 2020 Developmental gene expression as a phylogenetic data class: support for the monophyly of Arachnopulmonata. Dev. Genes Evol. 230:137-

153. (doi: 10.1007/s00427-019-00644-6)
5. It is not explained in the methods how synteny analysis was performed. Please explain the pipeline or the software used to make these inferences.
Minor comments:
1. Lines 40-41: "Spiders (Araneae) have a worldwide distribution, have conquered virtually all ecological environments". Please change this to read "virtually all terrestrial environments".
2. Line 62: "PacBio"
3. Line 254: "It has been reported that an ancient WGD event occurred in the spider lineage". Please replace this with "It has been reported that an ancient WGD event occurred in the common ancestor of spiders and scorpions".
4. Line 269: "In metazoans, the 10 highly conserved Hox genes play important roles…" The number of Hox genes is highly variable across Metazoa. It is inferred that the common ancestor of Panarthropoda bore 10 Hox genes, though this number varies across arthropod lineages as well. I suggest to change this to: "In arthropods, 10 highly conserved Hox genes that are inferred to occur in the common ancestor of Panarthropoda play important roles…"
5. Lines 459-460: On the WGD events in Xiphosura, please be sure to cite two recent papers (both Shingate et al. 2020) on the most recent and high-quality genomes of Carcinoscorpius and one of the Tachypleus species. There is additional evidence therein that the Xiphosura WGD events are independent of the Arachnopulmonata WGD event.
In summary, I support publication of this work after minor revision.
Prashant P. Sharma

Level of Interest

Please indicate how interesting you found the manuscript: Choose an item.

Quality of Written English

Please indicate the quality of language in the manuscript: Choose an item.

Declaration of Competing Interests

Please complete a declaration of competing interests, considering the following questions:

- Have you in the past five years received reimbursements, fees, funding, or salary from an organisation that may in any way gain or lose financially from the publication of this manuscript, either now or in the future?
- Do you hold any stocks or shares in an organisation that may in any way gain or lose financially from the publication of this manuscript, either now or in the future?
- Do you hold or are you currently applying for any patents relating to the content of the manuscript?

- Have you received reimbursements, fees, funding, or salary from an organization that holds or has applied for patents relating to the content of the manuscript?
- Do you have any other financial competing interests?
- Do you have any non-financial competing interests in relation to this paper?

If you can answer no to all of the above, write 'I declare that I have no competing interests' below. If your reply is yes to any, please give details below.

I declare that I have no competing interests.

I agree to the open peer review policy of the journal. I understand that my name will be included on my report to the authors and, if the manuscript is accepted for publication, my named report including any attachments I upload will be posted on the website along with the authors' responses. I agree for my report to be made available under an Open Access Creative Commons CC-BY license (http://creativecommons.org/licenses/by/4.0/). I understand that any comments which I do not wish to be included in my named report can be included as confidential comments to the editors, which will not be published.

Choose an item.

To further support our reviewers, we have joined with Publons, where you can gain additional credit to further highlight your hard work (see: https://publons.com/journal/530/gigascience). On publication of this paper, your review will be automatically added to Publons, you can then choose whether or not to claim your Publons credit. I understand this statement.

Yes Choose an item.
