# Supplementary material for: A chromosome‐level genome of the spider Trichonephila antipodiana reveals the genetic basis of its polyphagy and evidence of an ancient whole-genome duplication event
Source: Gigascience. 2021 Mar 19;10(3):giab016. doi: 10.1093/gigascience/giab016 (PMC7976613; doi:10.1093/gigascience/giab016)
Supplement: giab016_Reviewer_2_Report_Original_Submission — Jesper Bechsgaard -- 12/10/2020 Reviewed [file giab016_reviewer_2_report_original_submission.pdf]

Reviewer Report

Title: A chromosomeâ€level genome of the spider Trichonephila antipodiana reveals the genetic basis of its polyphagy and evidence of an ancient whole-genome duplication event

Version: Original Submission **Date:** 12/10/2020

Reviewer name: Jesper Bechsgaard

Reviewer Comments to Author:

A chromosome-level genome of the spider Trichonephila antipodiana reveals the genetic basis of its polygaphy and evidence of an ancient whole-genome duplication event
The 'Background' section do not really build up to the idea of polyphagy and detoxification abilities. Consequently, I am, as not being an expert in this field, not convinced about the idea that this species should have high detoxification abilities due to polyphagy, and that the detoxification related gene families actually are detoxification related gene families. The 'Background' is mainly a patchwork of more or less specific information about spiders. Immune system, sexual dimorphism, how many spider genomes etc does not really provide much useful information for this study. It would be nice if the authors could collect information from other studies about food ranges (polyphagy), how that can be toxic, and what mechanisms/genes exist to overcome toxicity. If possible even couple it with genome duplication. That may enable them to come up with some hypotheses about what to find in this genome.
If the authors could provide an analysis including a number of other arthropods, that could demonstrate a correlation between food niche width and evolution of the gene families assumed to be involved in detoxification, it would strengthen their main conclusion a lot.
The whole-genome duplication has been shown before, so I believe that making this a major point of this study (even putting it in the title) is too much. Fair enough to provide data that confirms previous findings and extent the analyses with better data (chromosome level assembly).
The use of references are not always good. For example:
-line 43: production of silk - they cite 'Recent Advances in Development of Functional Spider Silk-Based Hybrid Materials'
-line 45: whole genome duplication - they cite a paper (that is not yet peer-reviewed) that do not show the whole-genome duplication event, while a 2017 paper showed just that (https://bmcbiol.biomedcentral.com/articles/10.1186/s12915-017-0399-x?utm_campaign=BMC40864B&utm_medium=BMCemail&utm_source=Teradata)
There are many more examples!
I am convinced that the suite of analyses performed is done well!
To me the 'Conclusion' is more a repetition of results than a real conclusion.
Minor comments:
Line 52: + Stegodyphus mimosarum and Acanthoscurria geniculate (https://onlinelibrary.wiley.com/doi/full/10.1111/jeb.12780)
Line 61-62: Also Stegodyphus dumicola (https://www.mdpi.com/2073-4425/10/2/137)

Line 101: what muscle tissue? From a leg?
Line 254: not in the spider lineage, but in the ancestor of spiders and scorpions.
Line 302: I think Table 4 is wrong here.
Line 317: I only see 4 tables in the manuscript.
Line 331: numbers of exons and introns per gene seem wrong.
Line 378: I am not sure the ref12 demonstrate a powerful spider immune response?
Line 460: right references?
Line 464: ref should be 77.

Level of Interest

Please indicate how interesting you found the manuscript: Choose an item.

Quality of Written English

Please indicate the quality of language in the manuscript: Choose an item.

Declaration of Competing Interests

Please complete a declaration of competing interests, considering the following questions:

- Have you in the past five years received reimbursements, fees, funding, or salary from an organisation that may in any way gain or lose financially from the publication of this manuscript, either now or in the future?
- Do you hold any stocks or shares in an organisation that may in any way gain or lose financially from the publication of this manuscript, either now or in the future?
- Do you hold or are you currently applying for any patents relating to the content of the manuscript?
- Have you received reimbursements, fees, funding, or salary from an organization that holds or has applied for patents relating to the content of the manuscript?
- Do you have any other financial competing interests?
- Do you have any non-financial competing interests in relation to this paper?

If you can answer no to all of the above, write 'I declare that I have no competing interests' below. If your reply is yes to any, please give details below.

I declare that I have no competing interests

I agree to the open peer review policy of the journal. I understand that my name will be included on my report to the authors and, if the manuscript is accepted for publication, my named report including any attachments I upload will be posted on the website along with the authors' responses. I agree for my

report to be made available under an Open Access Creative Commons CC-BY license (http://creativecommons.org/licenses/by/4.0/). I understand that any comments which I do not wish to be included in my named report can be included as confidential comments to the editors, which will not be published.

Choose an item.

To further support our reviewers, we have joined with Publons, where you can gain additional credit to further highlight your hard work (see: https://publons.com/journal/530/gigascience). On publication of this paper, your review will be automatically added to Publons, you can then choose whether or not to claim your Publons credit. I understand this statement.

Yes Choose an item.
